# Dual wavelength asymmetric photochemical synthesis with circularly polarized light†
†Electronic supplementary information (ESI) available: Full detailed methods used for the entire study; further discussion of the work not central to the main message of the paper; full derivation of the kinetics models used to predict the dual wavelength enantioselectivity; computational details and energy breakdown; more complete mechanism for the reaction. See DOI: 10.1039/c4sc03897e


**DOI:** 10.1039/c4sc03897e

**Published:** 2015-04-16

**Authors:** Robert D. Richardson, Matthias G. J. Baud, Claire E. Weston, Henry S. Rzepa, Marina K. Kuimova, Matthew J. Fuchter

**Affiliations:** a Department of Chemistry , Imperial College London , South Kensington Campus , London , SW7 2AZ , UK . Email: m.kuimova@imperial.ac.uk ; Email: m.fuchter.imperial.ac.uk

## Abstract

Asymmetric photochemical synthesis using circularly polarized (CP) light is theoretically attractive as a means of absolute asymmetric synthesis and postulated as an explanation for homochirality on Earth. Using an asymmetric photochemical synthesis of a dihydrohelicene as an example, we demonstrate the principle that two wavelengths of CP light can be used to control separate reactions. In doing so, a photostationary state (PSS) is set up in such a way that the enantiomeric induction intrinsic to each step can combine additively, significantly increasing the asymmetric induction possible in these reactions. Moreover, we show that the effects of this dual wavelength approach can be accurately determined by kinetic modelling of the PSS. Finally, by coupling a PSS to a thermal reaction to trap the photoproduct, we demonstrate that higher enantioselectivity can be achieved than that obtainable with single wavelength irradiation, without compromising the yield of the final product.

## Abstract 2

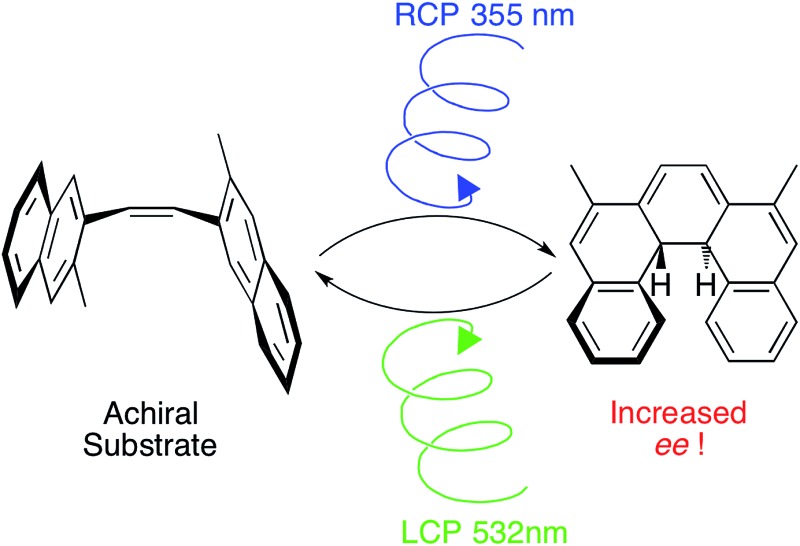
An asymmetric photchemical synthesis of a dihyrohelicene demonstrates two wavelengths of circularly polarized (CP) light can be used to ensure the enantiomeric induction intrinsic to each step can combine additively; significantly increasing the asymmetric induction possible over a single wavelength approach.

## Introduction

There has been considerable interest in the use of circularly polarized (CP) light, chiral electromagnetic radiation, to mediate asymmetric photochemistry.^1^,^2^ Indeed, the interaction of CP light with interstellar primordial molecules (ultimately combined with an amplification mechanism) is one hypothesis for the evolution of homochirality on Earth.1c,^3^ It has long been established that three distinct mechanisms exist for the induction of asymmetry using CP light: (1) preferential photodestruction,^4^ in which one of the enantiomers of a racemate is preferentially photodestroyed and the other remaining stereoisomer is therefore enriched; (2) photoresolution, namely a deracemization process of photochemically interconvertable enantiomers; (3) asymmetric photochemical synthesis,^5^ namely the enantioselective photochemical formation of an optically active compound from a prochiral starting material.^1^,^6^,^7^

The most common method for the preparation of enantioenriched compounds using CP light is by asymmetric photodestruction. Significant asymmetric induction however, often requires high conversion (>99%), leaving just 1% of an enantiomerically enriched substance.^6^,^8^ Nonetheless, this method continues to be of significant interest, with an example being the recent report by Meinert of the asymmetric kinetic resolution of amino acids using short-wavelength CP UV light;^9^ highly applicable to homochirality on Earth. By coupling an asymmetric photodestruction reaction to an amplifying step, high *ee* products have been obtained, recent examples either employing an autocatalytic *ee*-amplifying Soai reaction,^10^ or physical grinding processes.^11^

Alternatively, non-destructive photoresolution methods using CP light continue to be reported in a variety of applications, including in organic^12^ and inorganic^13^ chiroptical switches, or to obtain chiral coordination complexes^14^ and conjugated polymers.^15^ In such reactions, the *ee* could be amplified by supramolecular chirality^16^ or by using photoswitchable compounds that induced chirality in helical polymers,^17^,^18^ liquid crystals^19^ or that acted as seeds in a Soai reaction.^20^

Potentially, asymmetric photochemical synthesis is the most appealing method of those available because of its simplicity—only requiring a prochiral starting material—and the fact that it does not waste large quantities of material through photodestruction. To successfully induce asymmetry in photochemical synthesis however, the prochiral reactant must exist in rapidly (*i.e.*, faster than the rate of photon absorption) interconverting enantiomeric conformations, which can be selectively excited with CP light.^1^ Two enantiomeric Franck–Condon (FC) regions are thus occupied in an unequal ratio that is preserved on conversion to product, provided that the passage from the FC regions to the products is faster than the rate of excited state racemization.

**Scheme 1:**
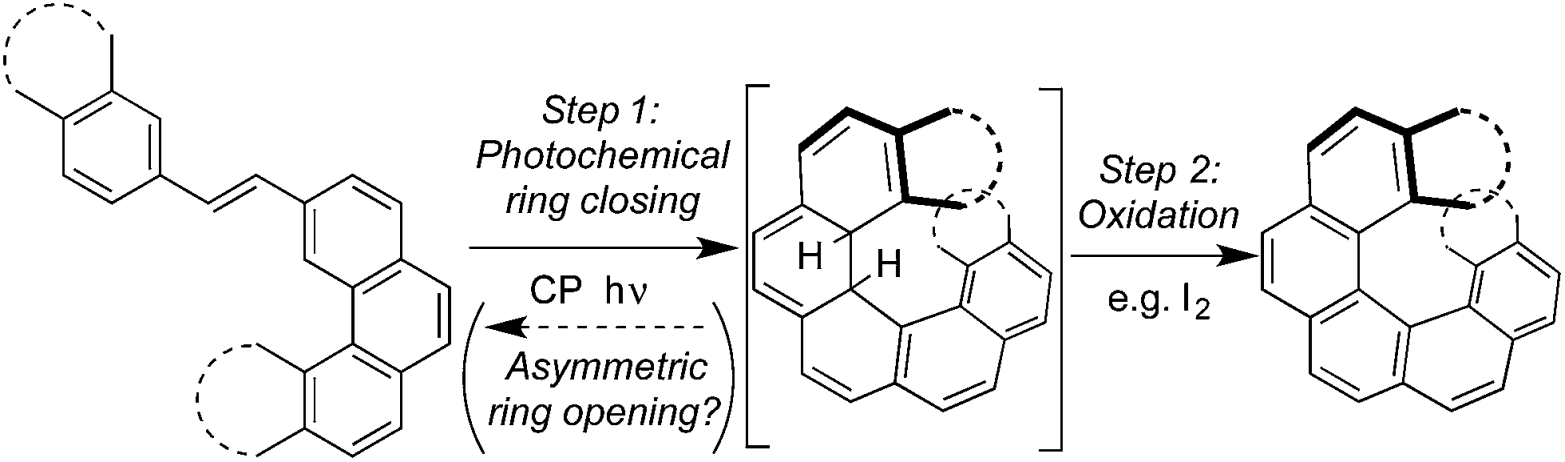
Asymmetric photochemical synthesis of helicenes using CP light.

To the best of our knowledge, the synthesis of optically enriched helicenes by one-pot oxidative photocyclization of diarylethylenes remained, for decades, the only known example of asymmetric photochemical synthesis using CP light.^1^ Extensive studies by the groups of both Kagan^6^–^8^ and Calvin^21^ have demonstrated the effective asymmetric synthesis of a range of helicenes by irradiating the diarylethylene substrates with CP light, in the presence of iodine as an oxidant (Scheme 1). The products of this reaction, helicenes, have recently found application in CP emissive^22^ and CP responsive organic electronic devices.^23^ Outside of small molecule chemistry however, there are recent efforts to use CP light to promote asymmetric photochemical synthesis of polymers.^24^

For all processes involving CP light, the enantioselectivity obtained is ultimately limited by the Kuhn anisotropy (*g*-) factors^25^ (*g* = Δ*ε*/*ε*, where Δ*ε* is the difference in extinction coefficients for left- (LCP) and right-handed (RCP) CP light for a single enantiomer or enantiomeric conformation). These are typically small, resulting in low selectivity between the excitation of each enantiomer (or enantiomeric conformation).^1^ The preparation of hexahelicene,^7^ for example, gave a product *ee*^26^ of 0.0035 (0.35%) and the photochemical synthesis of octahelicene^21^ gave an *ee*^26^ of 0.02 (2%); both are a result of the small *g*-factors for π–π* transitions in simple aromatic molecules (typically <0.01). The lower enantioselectivity achieved for hexahelicene in comparison to octahelicene likely reflects reduced *g*-factors in the enantiomeric conformations of key hexahelicene precursors (see ESI, Section S6.3† for further discussion).^27^

Mechanistically, the origin of stereocontrol in such reactions is believed to lie in the photochemical ring closing reaction (Scheme 1, Step 1, ‘forward’ reaction), with the subsequent *in situ* oxidation (Step 2) rapidly converting the dihydrohelicene intermediate to a photochemically stable product.^21^ It is well known, however, that photochemical electrocyclizations of this type are photochemically reversible.^28^,^29^ Therefore, in principle, the photochemical ring opening reaction (Step 1, ‘backward’ reaction), could provide a (currently unexploited) further opportunity for asymmetric induction.

**Scheme 2:**
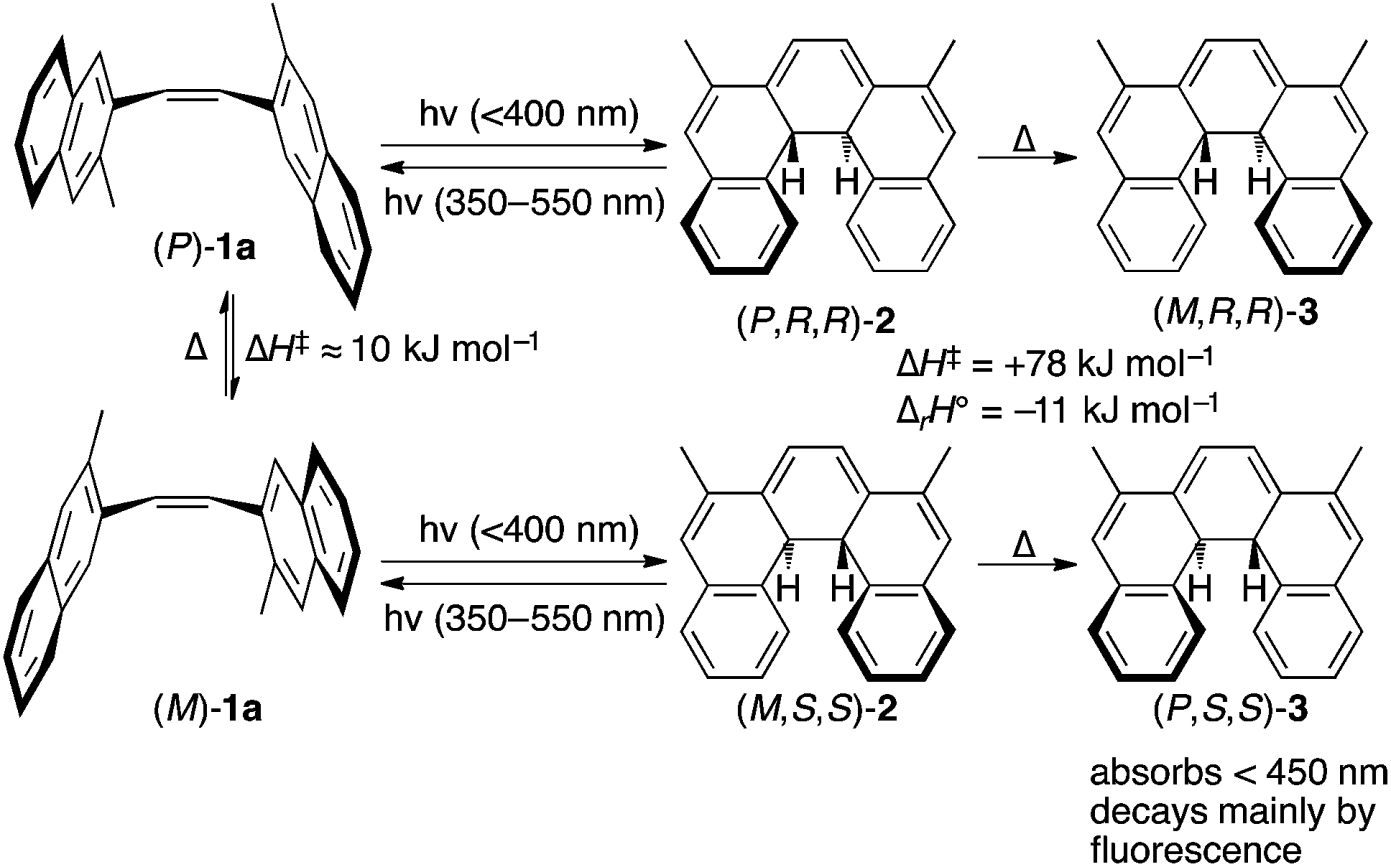
Mechanism and CP light opportunities for asymmetric induction in the photochemical synthesis of dihydrohelicenes. **1a** refers to the specific (chiral) reacting conformations of the *cis*-alkene whereas **1** is used to refer to the compound in all its conformations (see ESI, Fig. S2† for an expanded reaction scheme). Enthalpies cited come from DFT calculations (see ESI, Section S6.2†). In the absence of a stoichiometric oxidant, dihydrohelicene **3** should be the final product of the reaction, avoiding problems with competing reactions and product racemization.^28^,^29^

We set about exploring the ability to exploit multiple means of asymmetric induction with CP light, using *cis*-alkene **1**. As shown in Scheme 2, *cis*-dinaphthylethene^28^**1** exists in two enantiomeric conformations (*P*)-**1a** and (*M*)-**1a** (among others, see ESI, Section S3†) that can undergo the conrotatory ring closing to give enantiomeric dihydrohelicenes (*P*,*R*,*R*)-**2** and (*M*,*S*,*S*)-**2** respectively.^28^ Dihydrohelicene **2** can undergo a photochemical ring-opening reaction by light of a longer wavelength (450 ≤ *λ* ≤ 550 nm). Finally, enantiomeric dihydrohelicenes (*P*,*R*,*R*)-**2** and (*M*,*S*,*S*)-**2** undergo essentially irreversible thermal helix-inversion to give (*M*,*R*,*R*)-**3** and (*P*,*S*,*S*)-**3**.^28^,^29^ Inverted dihydrohelicene **3** is believed to be photochemically inactive and has been shown to be thermally stable at 300 K.^28^ In this paper, we present a combined theoretical and experimental study that demonstrates, for the first time, that dual wavelength CP photochemistry can be conducted, such that the enantioselectivity obtained from each photochemical step can be combined additively. In addition, we show that such an effect need not come at the cost of product yield, provided that a thermal reaction can be used to trap the enantioenriched photoproduct in the photostationary state (PSS) to a (photo)stable species. We believe the proof of concept approach reported here provides a theoretical framework for assessing the suitability of other systems towards two-wavelength asymmetric photochemical synthesis.

## Results and discussion

### Theory and predictions

As would be required for effective asymmetric photochemical synthesis, DFT calculations predicted rapid racemization of the enantiomeric conformations of **1a** (Δ*H*^‡^ ≈ 10 kJ mol^–1^, Scheme 2). Such a barrier should ensure the enantiomeric conformations of **1a** interconvert at a rate that exceeds the rate of photon absorption, under experimentally plausible temperatures and concentrations. Furthermore, TDDFT excited-state geometry optimizations starting from the DFT-optimized geometries of **1a** and **2** suggested that the passage from both Franck–Condon regions on the S_1_ surface to the ground state products should be barrierless. This is consistent with the predictions of Muszkat^28^ and should circumvent the problem of excited state racemization, which would reduce the enantioselectivity observed in the asymmetric photochemical synthesis. Finally, the thermal isomerization of intermediate **2** to product **3** was predicted to be essentially irreversible (Δ_r_*H*° = –11 kJ mol^–1^) occurring through a transition state reminiscent of the *C*_s_-symmetric transition state for the racemization of helicenes^30^ with an enthalpy of activation predicted to be +78 kJ mol^–1^, suggesting that readily measurable reactions rates for this process are likely to be observed around room temperature.

**Fig. 1:**
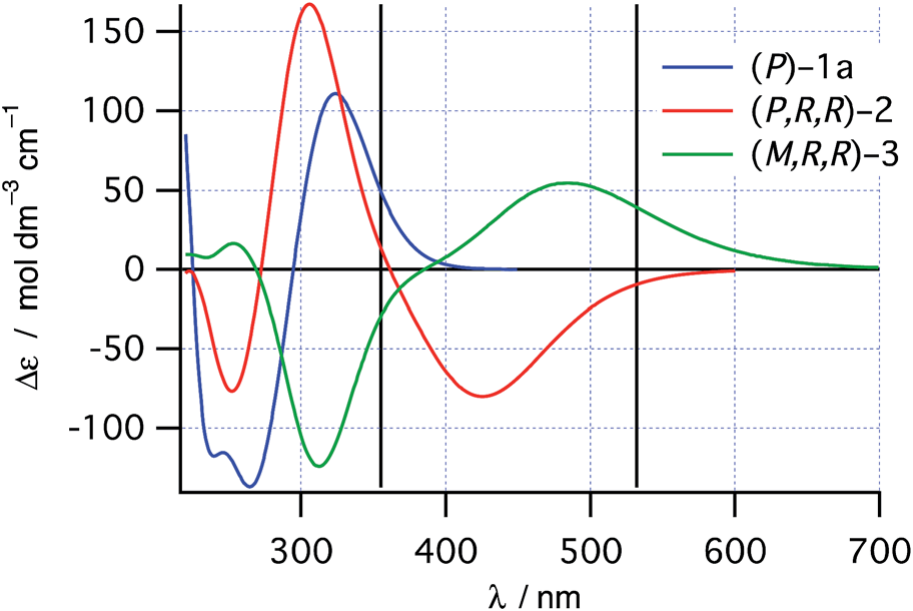
TDDFT predicted CD spectra for species in Scheme 2. Vertical lines mark the positions of the 355 nm and 532 nm Nd:YAG harmonics.

TDDFT single point calculations were used to predict the UV/vis and CD spectra for all relevant species depicted in Scheme 2. According to these spectra (Fig. 1), the Nd:YAG harmonics at 355 nm and 532 nm should selectively excite a single transition in **1a** and **2** respectively with predicted *g*-factors *g*355(*P*)-**1a** = +0.012 and *g*532(*P,R,R*)-**2** = +0.0077. Predicting *g*355(*P,R,R*)-**2** was difficult because two transitions with oppositely-signed *g*-factors overlap but, owing to this overlap, *g*355(*P,R,R*)-**2** is likely to be significantly less than the individual *g*-factors *g*355(*P*)-**1a** and *g*532(*P,R,R*)-**2**. Alkene **1** is predicted not to absorb light at 532 nm. Overall, these calculations suggested that the photochemistry of dinaphthylethene **1** is an (almost) ideal candidate for the proof-of-principle studies into the dual wavelength CP photochemistry, because the asymmetric induction in the ring closing and ring opening photochemical reactions should be controlled by balancing the handedness and power of the CP light at 355 nm and 532 nm respectively.

Based on the data generated in these DFT calculations and detailed kinetic modelling (see ESI, Sections S2 and S4†), the following predictions regarding the enantiocontrol under various experimental conditions were made:

1. Sole irradiation at 355 nm at low temperature (where no thermal inversion (**2** → **3**) occurs) should result in an *ee* of intermediate (*P*,*R*,*R*)-**2** of (*g*355(*P*)-**1a** – *g*355(*P,R,R*)-**2**)/2 in the PSS.^31^ The thermal inversion conducted subsequent to the photochemistry is stereospecific, so the *ee* of (*M*,*R*,*R*)-**3** obtained after inversion should equal that of (*P*,*R*,*R*)-**2** in the PSS.

2. At temperatures where the photochemical reactions and thermal inversion (**2** → **3**) occur simultaneously, the product *ee* obtained during sole irradiation at 355 nm should be temperature and power dependent: changing from *g*355(*P*)-**1a**/2 when the photochemical back reaction (**2** → **1**) is much slower than the thermal inversion (low power); to (*g*355(*P*)-**1a** – *g*355(*P,R,R*)-**2**)/2 when the photochemical ring opening (**2** → **1**) is much faster than the thermal inversion (high power). For such single wavelength irradiation at 355 nm, if *g*355(*P,R,R*)-**2** ≈ 0, the power will have no effect on the *ee*.

3. The *ee* of (*P*,*R*,*R*)-**2** in the PSS composition resulting from dual wavelength irradiation at 355 and 532 nm should depend on the relative powers and handedness of the 355 and 532 nm light. At a given 355 nm power, the *ee* obtained should change from (*g*355(*P*)-**1a** – *g*355(*P,R,R*)-**2**)/2 when 355 nm excitation dominates the ring opening reaction (**2** → **1**) (low 532 nm power), to (*g*355(*P*)-**1a** ∓ *g*532(*P,R,R*)-**2**)/2 when 532 nm excitation dominates the ring opening (high 532 nm power).^32^ The ∓ symbol indicates that the *g*-factors controlling the *ee* in the forward and backward reactions will either be additive or subtractive, depending on the relative sign of the *g*-factors for each transition, and the handedness of CP light employed at each wavelength. The selectivity in the ring opening changes from that induced at 355 nm to that induced at 532 nm as the relative flux through the reactions induced by those wavelengths changes (hence the relative laser power changes).

4. The use of plane polarized (PP) light at either wavelength in a dual-wavelength PSS effectively sets the *g*-factor at that wavelength to zero and the enantioselectivity should be solely determined by the remaining CP light wavelength. Hence, with CP 532 nm and plane polarized 355 nm in a dual wavelength PSS, the enantioselectivity should be *g*532(*P,R,R*)-**2**/2.

5. At temperatures where the dual wavelength photochemistry and thermal inversion occur simultaneously, the enantioselectivity obtained should change from *g*355(*P*)-**1a**/2 if the total rate of the photochemical back reaction (**2** → **1**) is much less than the rate of thermal inversion (low total power) to (*g*355(*P*)-**1a** ∓ *g*532(*P,R,R*)-**2**)/2 if the total rate of the photochemical back reaction (**2** → **1**) is much greater than the rate of thermal inversion (high total power).

### Photoswitching *cis*-**1** and **2**

**Fig. 2:**
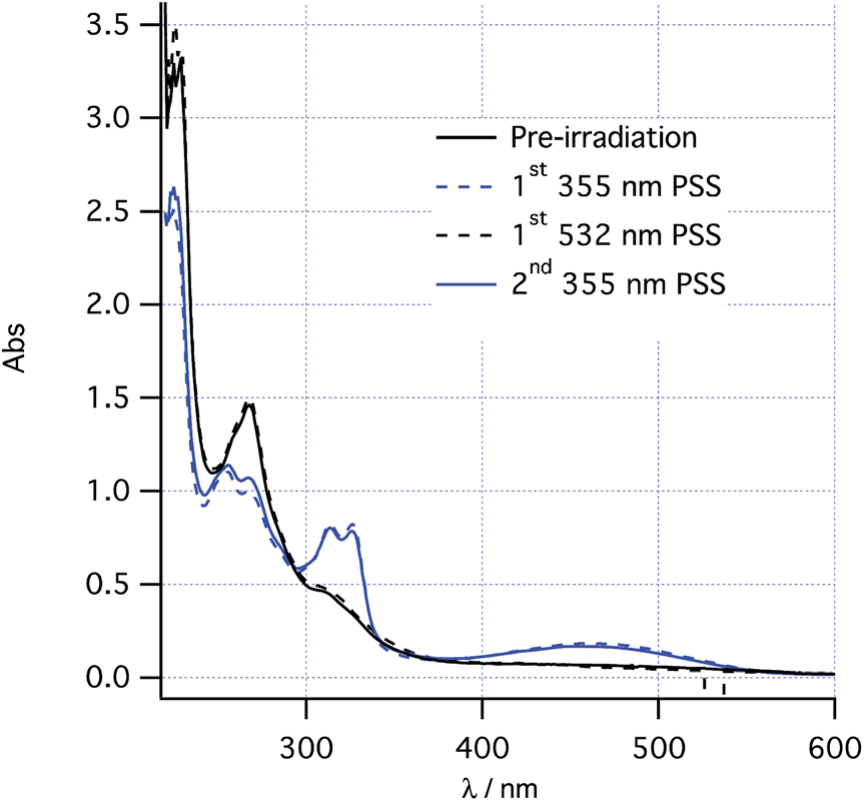
Photoswitching between *cis*-**1** and **2** at 260 K.

The proposed two-wavelength approach is dependent on the rates of various photochemical processes being carefully matched against the thermal processes. The kinetics of individual steps were therefore studied in greater detail in order to obtain a clear idea of the conditions required to maximize the probability of observing the predicted effects. At 260 K, the thermal helix inversion of photoproduct **2** to final product **3** is sufficiently slow that the system can be photoswitched using PP^33^ light between photostationary states consisting of pure *cis*-**1** and a 0.6 : 0.4 mixture of **2**/**1** (Fig. 2 and see “UV/vis spectra and PSS composition” section below). Such photoswitching occurs without formation of any other product by alternately irradiating at 532 nm and 355 nm at low power (<20 mW). At increased power, the absorbance around 350 nm is seen to increase upon 355 nm irradiation, tentatively assigned to a low quantum yield formation of *trans*-**1** from *cis*-**1** competing with the photochemical ring closing.

### Kinetics of thermal inversion of **2** to **3**

**Fig. 3:**
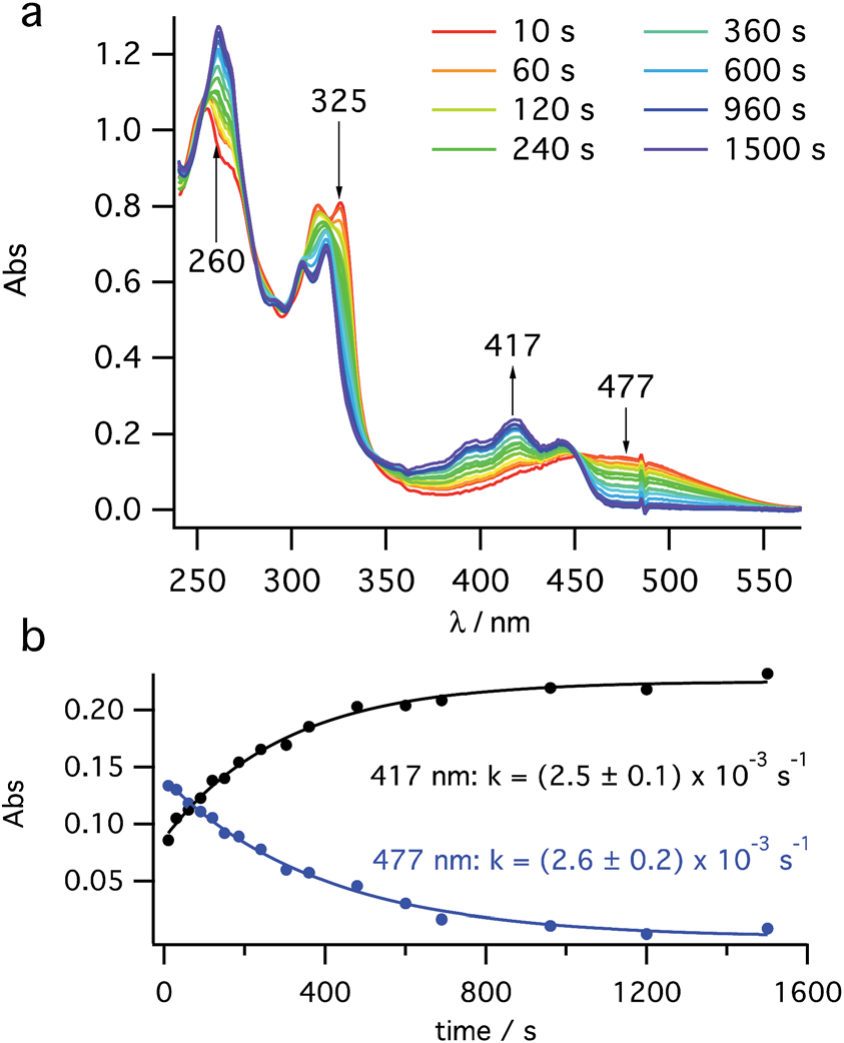
Kinetic study of thermal helix inversion (**2** → **3**) at 280 K. (a) UV/vis time course and (b) kinetics of the thermal inversion at two key wavelengths.

After a short burst (<10 s) of high power (100 mW) PP 355 nm light, to rapidly establish the PSS between *cis*-**1** and **2**, the kinetics of **2** → **3** helix inversion were measured. High power is defined as sufficient photon flux to reach the PSS within five seconds so that any thermal inversion occurring simultaneously to this is negligible. The data obtained show the decay of the UV signal (Fig. 3a) at 470 nm (assigned to **2**) with a simultaneous growth at 418 nm (assigned to **3**), consistent with the spectra reported by Muszkat^28^ for these species. This growth and decay occur with the same rate constant (Fig. 3b). It appears that the thermal inversion of **2** to **3** occurs cleanly and, essentially, quantitatively. Eyring analysis of the kinetics measured at six temperatures between 250 K and 300 K gives a straight line fit (see ESI, Fig. S11†) and activation parameters of Δ*H*^‡^ = +71.5 ± 2.2 kJ mol^–1^ and Δ*S*^‡^ = –38.7 ± 7.8 J K^–1^ mol^–1^. These values are consistent with Muszkat's work on related molecules^28^ and slightly lower than our DFT calculations. We note that the activation barriers for this helix inversion of dihydrohelicene **2** are lower than the calculated (see ESI, Section S7.1†) and experimental values for the racemisation of pentahelicene (Δ*H*^‡^ = +96 kJ mol^–1^ and Δ*S*^‡^ = –17 J K^–1^ mol^–1^ in iso-octane^34^).

### UV/vis spectra and PSS composition

**Fig. 4:**
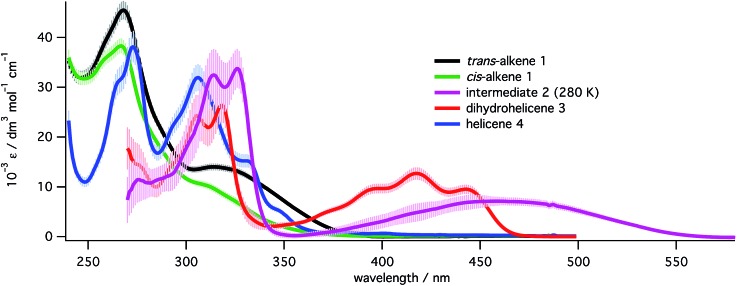
UV spectra for all species in the study at 293 K (except for **2**, measured at 280 K) in methylcyclohexane. Spectra for alkenes **1** and the corresponding [5]helicene **4** were recorded directly while spectra for **2** and **3** were extracted from mixtures (see ESI, Section S5.8†). Error bars represent the estimated uncertainty (±1 s.d.) in the extinction coefficients at that wavelength.

In order to quantify each species present in each photoreaction, UV spectra had to be obtained for all the species in Scheme 2. Spectra (Fig. 4) for *trans*-**1** and *cis*-**1** were obtained directly from pure materials^28^ and that of helicene **4** was obtained after an oxidative photochemical synthesis from a mixture of isomers of the starting alkene **1**, after purification and characterization (see ESI, Section S5.8†). Following PP 355 nm irradiation of a high concentration (approx. 3 mg mL^–1^) solution of **1** and partial purification from the excess starting materials (see ESI, Section S5.8.3†), a mixture of *cis*-**1**, dihydrohelicene **3** and helicene **4** was obtained whose concentrations in C_6_D_6_ could be determined by ^1^H NMR against an internal standard. Dilution of this sample into methylcyclohexane and deconvolution of the UV/vis spectrum of the mixture allowed determination of the UV/vis spectrum of **3**. The UV/vis spectrum of intermediate **2** was then determined from the spectra of the 355 nm photolysate before and after thermal inversion, assuming the inversion of **2** to **3** to be quantitative (see “Kinetics of thermal inversion of **2** to **3**”, above).

After high power 355 nm irradiation (100 mW), followed by thermal inversion the resulting photolysate was determined, using the UV spectra above, to contain only *cis*-**1** and dihydrohelicene **3** with a conversion to **3** of 0.59 ± 0.05. Assuming quantitative thermal inversion of **2** to **3**, this suggests that the 355 nm PSS composition contains a 0.59 ± 0.05 : 0.41 ± 0.05 mixture of **2** and *cis*-**1**.

### Ring opening of 2 at 532 nm

With the 355 nm photochemistry and thermal helix inversion studies in place, attention was turned to the effect of 532 nm light. Sole 532 nm irradiation of **1** does not form product, which is expected since **1** does not absorb light at 532 nm (Fig. 4). Alternatively, irradiating at 355 nm for a short time at high power to establish the PSS then monitoring the decay of absorbance at 470 nm (due to **2** alone) when irradiated with 532 nm laser at varying powers, allowed the rate of decay of **2** to be determined as a function of laser power. Fitting to a single exponential model shows the observed rate constant increases linearly with laser power (see ESI, Section S7.3†). Using the extinction coefficients from the deconvoluted spectra (Fig. 4) and the apparent first-order expression for the bulk photochemistry kinetics at low absorbance (see ESI, Section S2.2.4†), the quantum yield for the 532 nm ring opening back reaction was determined to be 0.31 ± 0.03 at 280 K.

### Asymmetric induction at CP 355 nm

**Fig. 5:**
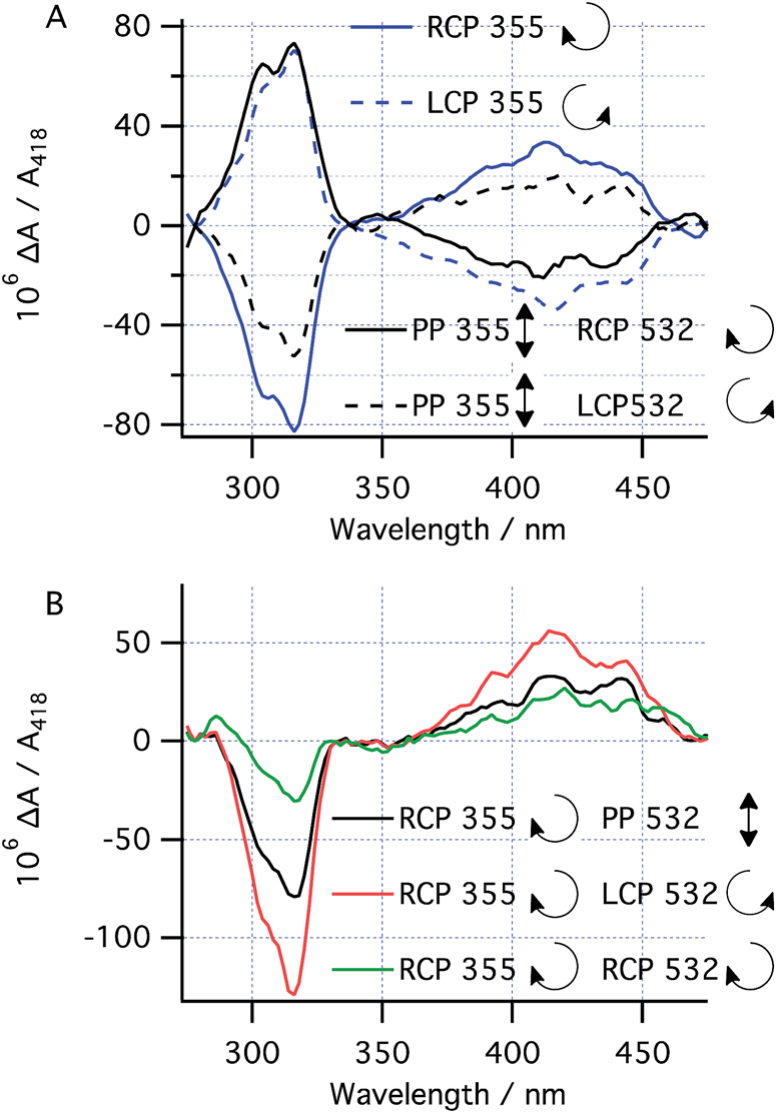
Concentration-corrected CD spectra of the helix-inverted product **3** using (A) CP 355 nm (100 mW) or plane polarized 355 nm (100 mW) + CP 532 nm (50 mW) or (B) combinations of CP 355 nm (100 mW) and CP 532 nm (50 mW) in short burst (10 s) irradiation to establish the PSS between **1** and **2** before allowing the thermal helix inversion. The magnitude of the CD peaks corresponds linearly to the enantiomeric excess of **3** in the sample and, hence, of that of **2** in the PSS. The arrows next to RCP/LCP in (A) indicate the direction of propagation of the electric vector of the CP light in time looking towards the source.

With the kinetic parameters of the various processes under study in hand, the asymmetric photochemistry of **1** using CP light was investigated. After high power, short duration (“burst”) irradiation (100 mW) of **1** using CP 355 nm light (to establish the PSS), followed by thermal helix inversion, CD spectra were obtained after irradiation with both LCP and RCP 355 nm (Fig. 5a). The CD peaks obtained closely matched the UV peaks for **3** (Fig. 4) and the CD spectrum obtained is cleanly inverted when the handedness of the 355 nm light is switched from left (LCP) to right (RCP) CP light. This result indicates that only the handedness of the CP 355 nm light is responsible for the chirality of the final product. Under these conditions, prediction 1 should hold and therefore the absolute magnitude of the asymmetry induced is expected to be determined by (*g*355(*P*)-**1a** – *g*355(*P,R,R*)-**2**)/2. As for the case with PP 355 nm light, the conversion to **3** after helix inversion was 0.59 ± 0.05.

### Asymmetric induction at 532 nm

We set out to establish the asymmetry induced by CP irradiation at 532 nm. As shown above, **1** does not absorb 532 nm light (Fig. 4). However, when CP 532 nm light is used in conjunction with PP 355 nm light, a dual wavelength PSS with enantioenriched **2** can be set up, where the enantioselectivity observed should be solely due to the *g*-factor associated with the photochemical ring opening driven by 532 nm light (*g*532(*P,R,R*)-**2** , see predictions 3 and 4). Laser powers of 100 mW PP 355 nm (to ensure the PSS should be reached within 5 s irradiation time) combined with 50 mW CP 532 nm were chosen because it was found to reduce the amount of intermediate **2** in the PSS by half—a compromise to enable observation of the asymmetric induction at 532 nm while leaving sufficient product **3** (after helix inversion) to allow for analysis.

Following burst irradiation under these conditions with both LCP and RCP 532 nm light (100 mW PP 355 nm, 50 mW CP 532 nm) and thermal helix inversion, CD spectra were obtained (Fig. 5a). These were comparable to those obtained with 355 nm CP irradiation, confirming the same enantioenriched product (**3**) in both experiments. Once again, the CD spectrum obtained is cleanly inverted when the handedness of the 532 nm light is switched from left to right (Fig. 5a). Comparing the amplitude of the CD peaks in Fig. 5a, indicates a greater stereoinduction by the photochemical ring closing reaction at 355 nm than in the ring opening reaction (532 nm), in accordance with the calculated *g*-factors (*g*355(*P*)-**1a** = +0.012 and *g*532(*P,R,R*)-**2** = +0.0077, Fig. 2). As a result of the halving of amount of **2** in the PSS by dual irradiation, half the conversion (0.29 ± 0.03) of **1** to **3** after helix inversion was obtained compared to CP 355 nm irradiation alone.

### Dual wavelength asymmetric induction

The effect of combining CP 355 nm and CP 532 nm light was then investigated. Using RCP 355 nm with PP 532 nm light, gave an asymmetric induction, after helix inversion, identical to that in the sample irradiated with CP 355 nm alone (Fig. 5a and b), suggesting that the ring opening reaction with CP 355 nm is equally unselective as that with PP 532 nm (see predictions 2 and 4), hence suggesting *g*355(*P,R,R*)-**2** ≈ 0. However, when RCP 355 nm light is combined with CP 532 nm light, the resulting asymmetric induction observed is either the sum or the difference of the stereoinduction using these wavelengths independently (Fig. 5b); the additivity depending on the relative handedness of the light at each wavelength (in accordance with the prediction 3). Thus, the combination of RCP 355 nm with LCP 532 nm almost doubles the asymmetric induction obtained. The fact that the opposite handedness of CP light at 355 nm and 532 nm is required for maximum asymmetric induction is a result of the identical sign of the *g*-factors *g*355(*P*)-**1a** and *g*532(*P,R,R*)-**2**.

While the original studies on the CP photochemical synthesis of octahelicene (Scheme 1)^21^ attributed the origin of stereocontrol to the forward photochemical ring closing reaction of diarylethylenes (followed by the rapid subsequent *in situ* oxidation to the helicene), our studies clearly indicate that asymmetric ring opening of intermediate **2** by CP light (at 532 nm) is an alternative means to induce asymmetry in such chemistry. Furthermore, we have shown that by judicious choice of the reaction conditions, it is possible to employ dual wavelength CP irradiation, such that the *g*-factors of the various photochemical steps involved operate additively, increasing the enantioselectivity of the overall reaction.

### Enantiomeric excess assignment

While the data in Fig. 5 shows this effect in a relative manner, the absolute enantioselectivity in this reaction is difficult to accurately quantify because the light and oxygen sensitivity^29^ of product **3** meant it could not be isolated in (enantio)pure form for chiroptical characterization.^35^ It should be noted, however, that these difficulties only cast uncertainty on the comparison to other work (whose *ee* measurements are also subject to uncertainty^21^) and not on the comparison of the additivity of the *ee* when comparing the single and dual wavelength photochemistry presented here. The comparison between the DFT-calculated *g*-factors and the experimental Δ*A*/*A* values (where *A* is the absorbance due only to product **3** and, where necessary, is extracted out using the extinction coefficients from Fig. 4) can, however, be used to estimate the *ee* values obtained (see ESI, Section S7.4†). Notably, the predicted Δ*A*/*A* values compare well to the experimental values (Table 1), especially considering the light for the photochemistry is measured at approximately 90% circularly polarized (see ESI, Section S5.2†). Based on this approach, the experimentally obtained enantiomeric excesses of **3** are estimated as 0.005 (CP 355 nm only), 0.003 (PP 355 nm with CP 532 nm) and 0.008 (RCP 355 with LCP 532).^26^ While the absolute magnitude of these values is firmly limited by *g*-factors, it should be noted that our dual wavelength approach enables more than double the *ee* obtained by Kagan in the photochemical synthesis of [6]helicene (0.0035).^6^

**Table 1:** Theoretical *vs.* experimental CD responses^*a*^

Condition	*λ*/nm	10^6^ Δ*A*/*A*	
		Calc.	Expt
LCP 355^*d*^	316^*b*^	–37	–38
	418^*c*^	+44	+34
PP 355	316^*b*^	–28	–26
LCP 532^*d*^	418^*c*^	+24	+21
RCP 355	316^*b*^	–72	–62
LCP 532^*e*^	418^*c*^	+61	+56

^*a*^Measured and predicted Δ*A*/*A* from the TDDFT *g*-factors (see ESI, Section S7.4).

^*b*^Δ*A*_316_/*A*_316_ is read from Fig. 5 and scaled according to the ratio of extinction coefficients at 418 nm and 316 nm from Fig. 4.

^*c*^The experimental Δ*A*_418_/*A*_418_ at 418 nm is read directly from Fig. 5.

^*d*^Magnitude determined from the average absolute value for RCP and LCP in Fig. 5a.

^*e*^Determined directly from Fig. 5b.

### CP irradiation with thermal inversion

While the combination of RCP 355 nm with LCP 532 nm almost doubles the asymmetric induction obtained in this reaction, the reliance on a PSS with an increased rate of ring opening results in a reduction in the overall conversion to product. To counter this, further dual irradiation experiments were conducted at 280 K, where the thermal inversion occurs on a useful timescale but still sufficiently slowly (*k*_inv_ = (2.5 ± 0.1) × 10^–3^ s^–1^, see “Kinetics of thermal inversion of **2** to **3**” above) that the PSS could be established much faster than the inversion at low laser powers. With these carefully selected conditions, it should be possible to use the thermal helix inversion to trap the enantioenriched photoproduct **2** in the PSS, giving a photostable species, thus recovering a high conversion to final product **3**. Irradiation of **1** with 20 mW RCP 355 nm light, in combination with 10 mW LCP 532 nm at 280 K (the 2 : 1 power ratio chosen for consistency with “Dual wavelength asymmetric induction” above), was carried out until the concentration of **3** (as judged by the absorbance at 418 nm) reached a maximum. Under these conditions, the establishment of the PSS is faster than the thermal inversion, and thus asymmetric induction should be governed by (*g*355(*P*)-**1a** ∓ *g*532(*P,R,R*)-**2**)/2 (as in the PSS, prediction 5). Accordingly, subsequent analysis by UV/vis and CD spectroscopy indicated that final product **3** was formed with the same enantioselectivity as in burst irradiation dual-wavelength photochemistry reported above. A significantly increased conversion of 0.45 ± 0.04 (*vs*. 0.29 ± 0.03, see “Dual wavelength asymmetric induction” above) was observed. This confirmed that the use of a thermal inversion step can partially compensate for the necessarily reduced conversion in the PSS during dual wavelength irradiation. The observed conversion was still lower than expected for clean photochemistry; ultimately attributed to photodestruction of **3** (see “Photodestruction of dihydrohelicene **3**” below). Reducing the power at both wavelengths (1.6 mW RCP 355 nm and 0.8 mW LCP 532 nm at 280 K) allowed the preparation of dihydrohelicene **3** with the same enantioselectivity as that obtained previously, but with a dramatically increased conversion (0.74 ± 0.07). Repeating this experiment using 1.6 mW 355 nm alone resulted in a conversion of 0.78 ± 0.07 showing that, under these low power conditions, the inclusion of 532 nm has little effect on the amount of product obtained. This clearly demonstrates that coupling of a thermal inversion process to the dual wavelength photochemistry ensures excellent conversion can be achieved without compromising on the enantioselectivity achieved in the PSS.

### Variation of laser power

When irradiating a sample of **1** at 280 K solely with CP 355 nm light at a range of powers, the asymmetric induction observed was found to be unchanged (see ESI, Section S7.3†). The lack of dependence of the *ee* on the 355 nm laser power, despite a change from a rate-determining ring closure to a rate determining helix inversion (see ESI, Section S5.2.1 and S2.5.2† for a discussion), is consistent with the photochemical ring opening back reaction being unselective at 355 nm (*i.e.*, *g*355(*P,R,R*)-**2** ≈ 0 see prediction 2).

**Fig. 6:**
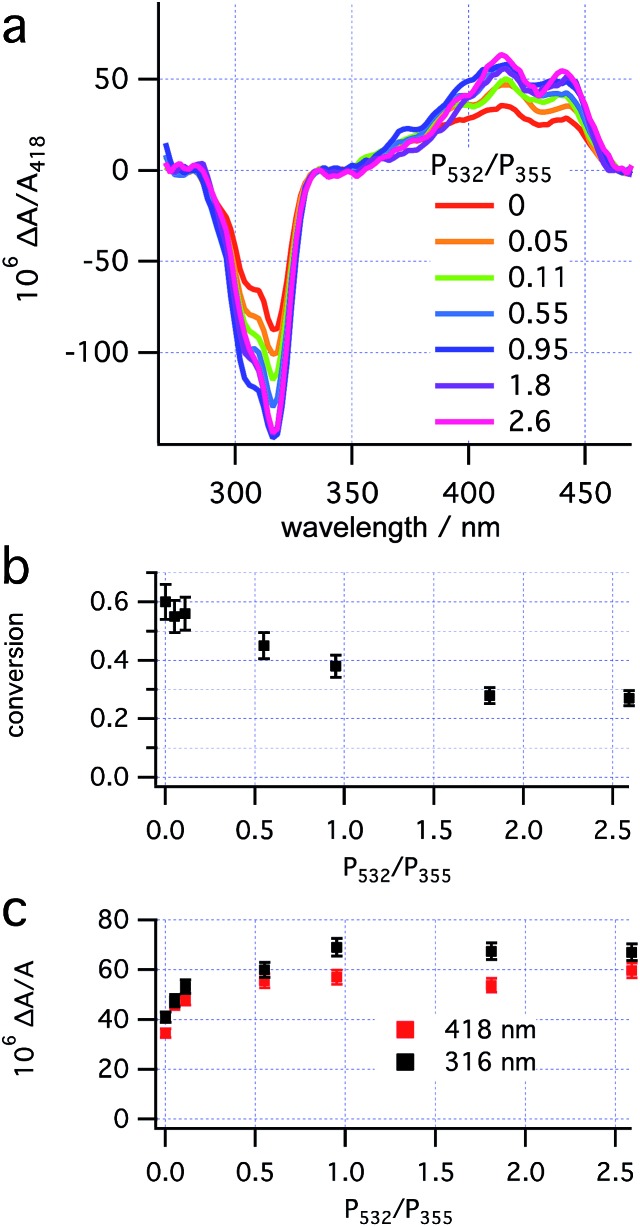
Effects of the power ratio (*P*_532_/*P*_355_, *P*_355_ ≈ 16 mW) on (a) concentration corrected CD spectra; (b) conversion to **3** and (c) enantioselectivity.

With the RCP 355 nm power maintained at 16 mW, the effect of the LCP 532 nm power on both conversion to **3** (deduced from the size of *A*_418_ after helix inversion) and enantioselectivity of **3** (deduced from Δ*A*/*A* at two wavelengths) was investigated at 280 K (Fig. 6). The data obtained indicate that the asymmetric induction increases upon increasing 532 nm power, up to approximately 8 mW, after which it plateaus. The maximal asymmetric induction is therefore observed at a power ratio *P*_532_/*P*_355_ > 0.5. This threshold is equal to the power ratios used in the preliminary experiments (see “Dual wavelength asymmetric induction” and “CP irradiation with thermal inversion” above).

The threshold power for maximum influence of the CP 532 nm light on the asymmetric induction in the photoreaction should be determined by two factors: the rate of the 532 nm-initiated opening of **2** → **1** must significantly exceed both that of the thermal helix inversion of **2** → **3**, and that of the (unselective, *g*355(*P,R,R*)-**2** ≈ 0) photochemical ring opening initiated by 355 nm light (see “Theory and predictions”). Because the reduction of laser power at both wavelengths (while maintaining the same ratio) does not lead to loss of enantioselectivity (see “CP irradiation with thermal inversion” above), it would appear that competition between the selective 532 nm (*g*532(*P,R,R*)-**2**) and (unselective) 355 nm (*g*355(*P,R,R*)-**2**) photochemical ring opening determines the threshold observed here. The PSS at the threshold under these conditions (16 mW 355 nm and 8 mW 532 nm) contains half the concentration of **2** as that with sole irradiation by 355 nm light (16 mW), suggesting that the rates of the photochemical ring opening initiated by 355 nm and 532 nm light are equal. The plateau of enantioselectivity should be expected where the rate of the ring opening initiated at 532 nm greatly exceeds that initiated at 355 nm and would, therefore, be expected to be at a significantly higher 532 nm power (approx. 30 mW). Overall, prediction 5 appears to be confirmed qualitatively, but not quantitatively. The enantioselective photodestruction of product **3** (see “Photodestruction of dihydrohelicene **3**” below and ESI, Section S7.5†) which should be more significant at increased 532 nm power is tentatively suggested as an explanation for this discrepancy.

### Photodestruction of dihydrohelicene **3**

**Fig. 7:**
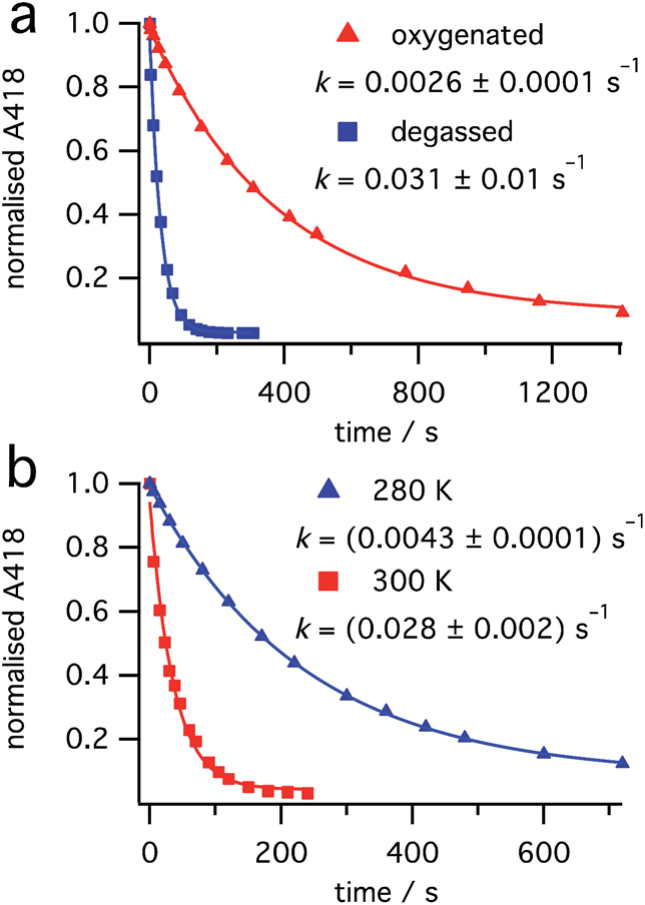
Photodestruction of **3** by 440 nm light (7.5 mW). Effect of (a) oxygen at 300 K and (b) temperature in the absence of oxygen.

In light of the reduced conversion observed at higher powers, we sought to investigate the photostability of the reaction products. Irradiation of an approximate 0.6 : 0.4 mixture of dihydrohelicene **3** and *cis*-alkene **1** (obtained from burst irradiation at 355 nm, followed by thermal inversion) at 440 nm, where only **3** absorbs, resulted in a first-order reduction in the concentration of dihydrohelicene **3**, as judged by reduction in the absorbance at 418 nm (see Fig. 7). This indicated that, contrary to previous results in which it was reported to be photostable,^28^ photodestruction of **3** could occur.

Surprisingly, thorough degassing of the sample prior to irradiation led to *faster* photodestruction (Fig. 7a). Time-resolved emission measurements at 1270 nm following 440 nm excitation of dihydrohelicene **3** in the presence of oxygen showed an emission, that disappeared when the sample was degassed, attributed to the weak phosphorescence of singlet oxygen O_2_(a^1^Δ_g_) (see ESI, Section S5.9†).^36^ At 275 K, the signal decayed monoexponentially with a lifetime of 23.5 ± 1.3 μs; slightly lower than that of authentic singlet oxygen generated by irradiation of known singlet oxygen sensitizer tetraphenylporphine (TPP),^37^ in the same solvent (275 K, methylcyclohexane, 26.7 ± 0.4 μs). This shows that dihydrohelicene **3** can act as a singlet oxygen sensitizer and provides a mechanism by which oxygen can retard an excited state reaction. The quantum yield of singlet oxygen of **3** is approximately 0.8 times that of TPP (see ESI, Fig. S7†) which itself generates singlet oxygen with a quantum yield of 0.7 in toluene.^37^ The apparent inhibition of the photodestruction of **3** by oxygen, along with the observation of singlet oxygen, suggests that the photodestruction of **3** occurs through a triplet excited state manifold.

Even in the absence of oxygen, the photodestruction of **3** at 440 nm proceeds much more slowly than the ring opening of **2** at equal photon flux (compare Fig. 7 with ESI, Sections S7.2 and S8.2†), despite a much larger extinction coefficient at the respective pump wavelengths (see Fig. 4). This suggests that the quantum yield for this photodestruction is much lower than that for the ring opening of **2** (0.31 ± 0.03, see “Ring opening of **2** at 532 nm” above). The photodestruction of **3** also shows a ten-fold rate increase between 280 K and 300 K (Fig. 7b), compared with a two-fold increase for the disappearance of **2** (ESI, Section S7.2†), despite little temperature effect on the extinction coefficient of **3**. Alongside the photodestruction, irradiation of **3** results in considerable fluorescence (see ESI, Fig. S8†)^38^ consistent with prior reports by Muszkat.^28^ Together with the reduced rate and quantum yield compared to the ring-opening of **2**, this suggests that the photodestruction reaction proceeds through an activation barrier on the excited state, unlike the barrierless ring opening of **2**.^25^ Overall, it would appear that excitation of dihydrohelicene **3** can result in a number of photophysical and photochemical processes: (1) radiative relaxation to the ground state; (2) intersystem crossing to a triplet state (followed by singlet oxygen sensitization); and (3) photochemical decomposition. As a result, oxygen has a fascinating and divergent effect on the outcome of the photochemistry of *cis*-dinaphthylethene **1**. While oxygen slows the photodestruction of product **3** through quenching of its triplet excited state, the resultant singlet oxygen may be capable of oxidizing dihydrohelicene **3** to the resultant helicene,^28^,^29^ thus reducing the conversion to **3**. Use of a triplet quencher that does not lead to singlet oxygen formation may allow increased conversion of **1** to product **3**, especially at higher laser powers. Further investigations into the photochemistry and photophysics of these dihydrohelicene products are currently underway.

## Conclusions

Of the photochemical steps possible in the asymmetric photochemical synthesis of dihydrohelicenes (Scheme 2), the photochemical ring closure of **1** to **2** by CP light at 355 nm and the ring opening of **2** to **1** by CP light at 532 nm can result in asymmetric induction. The ring opening of **2** is unselective using CP 355 nm. The degree and sense of enantioinduction in all cases are well predicted by TDDFT calculations. When CP light is used at two wavelengths to establish a photostationary state, the enantioselectivity obtained is half of the sum or difference of the *g*-factors for the two transitions used, provided that the rates of the photochemical and thermal processes are suitably balanced. Thus, by selecting for an ‘additive’ effect using two wavelengths of light of opposite handedness for the system under study, we were able to approximately double the asymmetric induction obtained for the product **3**. Furthermore, the use of a thermal reaction to convert the PSS concentration of intermediate **2** to a comparatively stable final product **3** enables high conversion, despite driving the PSS back towards the starting material, without a loss of enantioselectivity. While, in this work, such a thermal reaction is a simple helix inversion, it should not be necessary for this reaction to be intramolecular. For example, in the preparation of longer dihydrohelicenes, that invert more slowly but whose precursors possess higher *g*-factors,^27^ the use of an oxidant to convert the dihydrohelicene to a (non-racemizing)^39^ helicene could replace the thermal inversion, provided that the oxidant does not interfere with the photochemistry. A thermal intermolecular reaction would allow the reaction rate to be adjusted by varying the concentration of the other reagent (*e.g.* oxidant). However, the control of the relative rates of helix inversion and photochemistry presented here by controlling the temperature and photon flux is technically more simple.

While the absolute enantioselectivities in these reactions are low—limited by the *g*-factor of the processes under study—the principle of controlled use of multiple wavelengths of CP light simultaneously to significantly increase the enantioselectivity in a photochemical reaction has been established. Application of this principle to systems with increased *g*-factors could lead to useful enantioselectivities generally not seen in asymmetric photochemistry. Furthermore, given the recent growing interest in CP active molecules in chiroptical switches, materials and devices, the theoretical and experimental approach developed here should allow for prediction of photochemistry in which our methodology may prove useful.

## Experimental section

Brief notes on the experimental and computational methods can be found below. Complete experimental and computational methods can be found in the ESI, Sections S5 and S6† respectively.

### CP light irradiation experiments

532 and 355 nm plane polarised light was obtained from the second and third harmonics of a Q-switched Nd:YAG laser and were circularly polarised where necessary using multi-order quarter waveplates. 440 nm plane polarised light was obtained by pumping a dye laser (with Coumarin 120 dye in methanol) at 355 nm and this was circularly polarised using a quarter-wave Fresnel rhomb where necessary. All light sources were pulsed (10 Hz, ∼5 ns pulse duration) and the beam diameter at the sample was approximately 5 mm. Experiments in which the asymmetric induction was not under investigation were conducted with plane polarised (PP) light. For dual wavelength experiments, the beams were aligned to overlap at the center of the sample, but this overlap was found not to be crucial to the results.

### CD spectroscopy

CD spectroscopy was performed at 293 K after resting the sample at room temperature for at least 30 minutes to ensure complete thermal inversion of intermediate **2** to final product **3**. To concentration-correct the CD spectra (to allow *ee* comparison), UV spectra were recorded under identical conditions. CD spectra were averaged over at least 120 scans to ensure a reasonable signal-to-noise ratio for the samples of low *ee*. Spectra were baselined against a spectrum of the solvent in the same cuvette, then manually in Origin 8.3 using the “Peak Analyzer” function to correct for any effects of thermal fluctuation in the spectrometer optics.

### Computational methods

Ground state calculations were conducted using the B3LYP hybrid functional^40^,^41^ with a CPCM^42^ solvent model for methylcyclohexane. Geometry optimizations and frequency calculations were conducted using the 6-31G(d,p) basis set and single point calculations were performed with the larger 6-311G(2df,2p) basis set to refine the energy. TDDFT calculations were performed with the CAM-B3LYP^43^ functional with the same CPCM solvent model. Excitation energies were calculated using the 6-311G(2df,2p) basis set while excited state geometry optimizations starting from the ground state stationary geometries were performed using the 6-31G(d,p) basis set.

## Supplementary Material

Supplementary information

## References

[cit1] Feringa B. L., van Delden R. A. (1999). Angew. Chem., Int. Ed..

[cit2] Barron L. D. (1986). J. Am. Chem. Soc..

[cit3] Rubenstein E., Bonner W. A., Noyes H. P., Brown G. S. (1983). Nature.

[cit4] Kagan H. B., Fiaud J. C. (1988). Top. Stereochem..

[cit5] In several publications, the term “asymmetric photosynthesis” is used for this process

[cit6] Kagan H. B., Balavoine G., Moradpour A. (1974). J. Mol. Evol..

[cit7] Moradpour A., Nicoud J. F., Balavoine G., Kagan H. B., Tsoucaris G. (1971). J. Am. Chem. Soc..

[cit8] Balavoine G., Moradpour A., Kagan H. B. (1974). J. Am. Chem. Soc..

[cit9] Meinert C., Hoffmann S. V., Cassam-Chenaï P., Evans A. C., Giri C., Nahon L., Meierhenrich U. J. (2014). Angew. Chem., Int. Ed..

[cit10] Kawasaki T., Sato M., Ishiguro S., Saito T., Morishita Y., Sato I., Nishino H., Inoue Y., Soai K. (2005). J. Am. Chem. Soc..

[cit11] Noorduin W. L., Bode A. A. C., van der Meijden M., Meekes H., van Etteger A. F., van Enckevort W. J. P., Christianen P. C. M., Kaptein B., Kellogg R. M., Rasing T., Vlieg E. (2009). Nat. Chem..

[cit12] Rijeesh K., Hashim P. K., Noro S., Tamaoki N. (2015). Chem. Sci..

[cit13] Stevenson K. L., Verdieck J. F. (1969). Mol. Photochem..

[cit14] Wu S.-T., Cai Z.-W., Ye Q.-Y., Weng C.-H., Huang X.-H., Hu X.-L., Huang C.-C., Zhuang N.-F. (2014). Angew. Chem., Int. Ed..

[cit15] Wang Y., Sakamoto T., Nakano T. (2012). Chem. Commun..

[cit16] Yeom J., Yeom B., Chan H., Smith K. W., Dominguez-Medina S., Bahng J. H., Zhao G., Chang W.-S., Chang S.-J., Chuvilin A., Melnikau D., Rogach A. L., Zhang P., Link S., Král P., Kotov N. A. (2015). Nat. Mater..

[cit17] Kim M.-J., Shin B.-G., Kim J.-J., Kim D.-Y. (2002). J. Am. Chem. Soc..

[cit18] Li J., Schuster G. B., Cheon K.-S., Green M. M., Selinger J. V. (2000). J. Am. Chem. Soc..

[cit19] Huck N. P. M., Jager W. F., de Lange B., Feringa B. L. (1996). Science.

[cit20] Sato I., Sugie R., Matsueda Y., Furumura Y., Soai K. (2004). Angew. Chem., Int. Ed..

[cit21] Bernstein W. J., Calvin M., Buchardt O. (1972). J. Am. Chem. Soc..

[cit22] Yang Y., da Costa R. C., Smilgies D.-M., Campbell A. J., Fuchter M. J. (2013). Adv. Mater..

[cit23] Yang Y., da Costa R. C., Fuchter M. J., Campbell A. J. (2013). Nat. Photonics.

[cit24] Yang G., Han L., Jiang H., Zou G., Zhang Q., Zhang D., Wang P., Ming H. (2014). Chem. Commun..

[cit25] Kuhn W. (1930). Trans. Faraday Soc..

[cit26] To compare *ee* to the *g*-factor (–2 < *g* < 2), all *ee* values are given as fractions (–1 ≤ *ee* ≤ 1) rather than percentages. *ee* is defined for a single enantiomer, a negative sign shows the other enantiomer in excess

[cit27] Nakai Y., Mori T., Inoue Y. (2012). J. Phys. Chem. A.

[cit28] Muszkat K. A., Eisenstein M., Fischer E., Wagner A., Ittah Y., Lüttke W. (1997). J. Am. Chem. Soc..

[cit29] Muszkat K. A. (1980). Top. Curr. Chem..

[cit30] Grimme S., Peyerimhoff S. D. (1996). Chem. Phys..

[cit31] For single wavelength radiation, the *ee* is predicted to be that obtained by LCP radiation. If RCP radiation is used, the sign of this prediction should be reversed

[cit32] For dual wavelength radiation, the first wavelength is LCP. The second wavelength is expressed as either LCP (top symbol) or RCP (bottom symbol) to indicate control over the relative handedness

[cit33] Plane polarised (PP) light was used for all experiments in which asymmetric induction was not under investigation

[cit34] Goedicke C., Stegemeyer H. (1970). Tetrahedron.

[cit35] As an alternative strategy, oxidation of **3** to the corresponding helicene **4**, prior to enantioselectivity determination, would result in problems with rapid [5]helicene racemization during the CD analysis (see ref. 20)

[cit36] Ogilby P. R. (2010). Chem. Soc. Rev..

[cit37] Wilkinson F., Helman W. P., Ross A. B. (1993). J. Phys. Chem. Ref. Data.

[cit38] Reabsorption of this (possibly weakly CP) emitted light by **1** or **2** could influence the *ee* of the photochemistry. Under the low absorbance conditions used here, this effect is likely to be negligible

[cit39] Martin R. H., Marchant M. J. (1974). Tetrahedron.

[cit40] Becke A. D. (1993). J. Chem. Phys..

[cit41] Lee C., Yang W., Parr R. G. (1988). Phys. Rev. B: Condens. Matter Mater. Phys..

[cit42] Cossi M., Rega N., Scalmani G., Barone V. (2003). J. Comput. Chem..

[cit43] Yanai T., Tew D. P., Handy N. C. (2004). Chem. Phys. Lett..

