# Supplementary material for: Dual wavelength asymmetric photochemical synthesis with circularly polarized light
Source: Chem Sci. 2015 Apr 16;6(7):3853–62. doi: 10.1039/c4sc03897e (PMC5707454; doi:10.1039/c4sc03897e)
Supplement: Supplementary file 1 [file SC-006-C4SC03897E-s001.pdf]

Electronic Supplementary Material (ESI) for Chemical Science.
This journal is © The Royal Society of Chemistry 2015

Dual Wavelength Asymmetric Photosynthesis with Circularly Polarised Light
Electronic Supplementary Information

Robert D. Richardson, Matthias G. J. Baud, Claire E. Weston, Henry S. Rzepa, Marina K. Kuimova,* and Matthew J. Fuchter†

Department of Chemistry, Imperial College London, London, SW7 2AZ, United Kingdom

Contents

S1 Definitions and Sign Conventions **S4**
- S1.1 Circularly Polarized Light . . . S4
- S1.2 Kuhn Anisotropy (g-)Factor . . . S4
- S1.3 Enantiomeric Excess . . . S5

S2 Kinetic Modelling of CP Light Photostationary States **S6**
- S2.1 System under Investigation . . . S6
- S2.2 Rate of Photochemical Reactions . . . S6
  - S2.2.1 Assumptions . . . S6
  - S2.2.2 Simple Photochemical Reaction . . . S6
  - S2.2.3 Rate Law at High Absorbance . . . S7
  - S2.2.4 Rate Law at Low Absorbance . . . S8
  - S2.2.5 Rate Laws with Two or More Absorbing Species . . . S8
  - S2.2.6 Simple Enantioselective Photosynthesis using CP light . . . S9
- S2.3 Single Wavelength Enantioselective Photostationary States . . . S10
  - S2.3.1 Simple Photostationary State (PSS) . . . S10
  - S2.3.2 Asymmetric Photosynthetic PSS . . . S10
- S2.4 Dual Wavelength CP Light Photochemistry . . . S11
  - S2.4.1 Rate and PSS for Dual Wavelength Photochemistry . . . S11
  - S2.4.2 Enantioselectivity in a Photochemical Step Initiated by Two Wavelengths . . . S12
  - S2.4.3 Dual-Wavelength CP Light PSS . . . S13
- S2.5 Combined Thermal and Photochemistry . . . S15
  - S2.5.1 Enantioselective Synthesis at High Power . . . S15
  - S2.5.2 Enantioselective Synthesis at Low Power . . . S16

S3 Full Reaction Scheme **S17**

S4 Predictions for the Asymmetric Photosynthesis of 3 **S20**
- S4.1 Notes on Wavelengths, g-Factors and Kinetics . . . S20
- S4.2 Testable Predictions for the Asymmetric Photosynthesis . . . S20
  - S4.2.1 CP 355 nm PSS . . . S21
  - S4.2.2 CP 355 nm with Thermal Inversion . . . S21
  - S4.2.3 Dual Wavelength PSS . . . S21

*m.kuimova@imperial.ac.uk
†m.fuchter@imperial.ac.uk

S5 Experimental Methods S23
S5.1 Experimental Setup S23
S5.2 Light Generation S23
S5.3 Sample Preparation S24
S5.4 UV/Vis Spectroscopy S24
S5.5 CD Spectroscopy S24
S5.6 NMR Spectroscopy S25
S5.7 Procedures for Irradiation Experiments S25
S5.7.1 Photoswitching Between **1** and **2** S25
S5.7.2 Kinetic Experiments on the Thermal Inversion S25
S5.7.3 Competing Thermal Inversion and 532 nm Ring Opening S25
S5.7.4 PSS Experiments using High Power, Short Duration Irradiation S25
S5.7.5 Simultaneous Irradiation and Thermal Inversion S25
S5.7.6 Asymmetric Photodestruction of Intermediate **2** S26
S5.7.7 Photodestruction of Product **3** S26
S5.8 Quantification of Dihydrohelicene **3** in Photolysate S26
S5.8.1 Extinction Coefficient Spectra for *cis*- and *trans*-**1** S26
S5.8.2 Extinction Coefficient Spectrum for Helicene **4** S26
S5.8.3 Extinction Coefficient Spectrum for Dihydrohelicene **3** S28
S5.8.4 Extinction Coefficient Spectrum for Intermediate **2** S28
S5.9 Singlet Oxygen [$O_2(a^1\Delta_g)$] Detection S28
S5.10 Fluorescence Spectrum of Dihydrohelicene **3** S30

S6 Computational Methods S31
S6.1 General Computational Details S31
S6.2 Energies for **1**, **2**, **3** and **4** and Relevant Transition States S31
S6.3 Predicted Enantioselectivity and Helicene Length S31
S6.4 Racemization of (P)- and (M)-**1a** S32
S6.5 Energy Breakdown for all Calculated Structures S33

S7 Further Results and Discussion S34
S7.1 Kinetics of Dihydrohelicene Inversion S34
S7.2 Kinetics and Quantum Yields of Ring Opening Reaction S35
S7.3 Variable Power CP 355 nm Radiation During Thermal Inversion S35
S7.4 Determination of *ee* and Comparing Theory and Experiment S36
S7.5 Asymmetric Photodestruction of Dihydrohelicene with CP 440 nm Light S38

S8 Supplementary Data S39
S8.1 Kinetic Data for Thermal Inversion S39
S8.2 Competing Thermal Inversion and Ring Opening S39
S8.3 NMR and UV Spectra used for Characterization and Quantification S41
S8.3.1 ^1H NMR spectrum of helicene **4** in $CDCl_3$ S42
S8.3.2 ^{13}C NMR spectrum of helicene **4** in $CDCl_3$ S43
S8.3.3 DEPT-135 NMR spectrum of helicene **4** in C_6D_6 . . . . . S44
S8.3.4 ^1H NMR spectrum for quantification of helicene **4** *vs.* mesitylene S45
S8.3.5 ^1H NMR spectrum for quantification of dihydrohelicene **3** *vs.* mesitylene S46
S8.3.6 UV spectrum of diluted NMR sample from S8.3.4 into methylcyclohexane to extract extinction coefficients for dihydrohelicene **3** S47
S8.4 Atomic Coordinates for Computed Structures S47

List of Figures

S1 Left-handed circularly polarized light. S4
S2 Full reaction scheme. S17
S3 TDDFT Simulated CD of conformers of **1**. S19
S4 Experimental setup for irradiation experiments. S23
S5 UV/vis spectra for all species with uncertainties. S27
S6 Singlet oxygen detection. S29
S7 Singlet oxygen quantum yield. S30
S8 Fluorescence of **3**. S30
S9 Ground state enthalpy diagram. S32
S10 Racemization of (P)- and (M)-**1a**. S33
S11 Eyring plot for the thermal inversion of intermediate **2** to product **3**. S34
S12 Competing thermal and photochemical reactions of **2**. S36
S13 Effect of 355 nm power on formation rate and *ee* of **3**. S37
S14 Thermal inversion kinetics as varying temperature S39
S15 Photodestruction of **2** with varying 532 nm power at 300 K S40
S16 Photodestruction of **2** with varying 532 nm power at 280 K S40
S17 Photodestruction of **2** with varying 532 nm power at 260 K S41

List of Tables

S1 Computed conformations of cis-dinaphthylethene **1**. S18
S2 Calculated energy breakdown. S33
S3 g-Factor data used to generate Table 1 (main text). S38
S4 Calculated atomic coordinates for (P)-**1a** S48
S5 Calculated atomic coordinates for the TS connecting (P)-**1a** and (P, R, R)-**2** S49
S6 Calculated atomic coordinates for (P, R, R)-**2** S50
S7 Calculated atomic coordinates for TS connecting (P, R, R)-**2** and (M, R, R)-**3** S51
S8 Calculated atomic coordinates for (M, R, R)-**3** S52
S9 Calculated atomic coordinates for (M)-**4** S53
S10 Calculated atomic coordinates for TS for racemization of **4** S54
S11 Calculated atomic coordinates for conformer **1b** S55
S12 Calculated atomic coordinates for TS connecting (P)-**1a** with **1b** S56
S13 Calculated atomic coordinates for the TS for racemization of **1b** S57

S1 Definitions and Sign Conventions

S1.1 Circularly Polarized Light

Left-handed circularly polarized (LCP) light refers to light where the electric vector propagates as a spatially left-handed helix as defined using a left-hand grip rule pointing along the direction of light propagation (Figure S1). When viewed towards the light source, the electric vector of the light is seen to propagate counter-clockwise in time. Right-handed circularly polarized (RCP) light forms the enantiomeric helix.

Figure S1: Left-handed circularly polarized light.

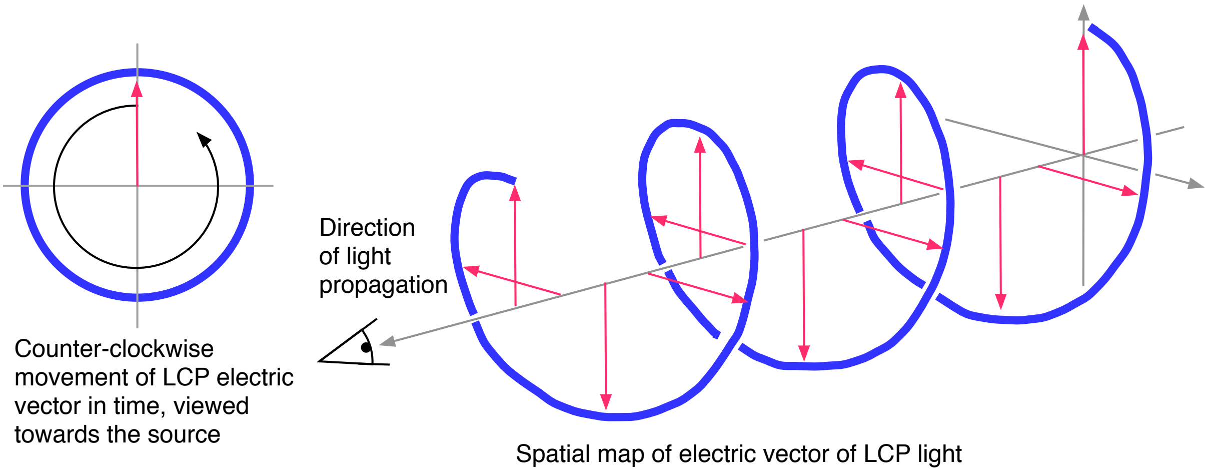

S1.2 Kuhn Anisotropy (g-)Factor

For a compound, A, existing as two enantiomers, A_R and A_S, the Kuhn anisotropy factor[1] (g where $-2 < g < +2$) is defined for a single enantiomer at a given wavelength, λ as:

$$g^{\lambda}_{\mathrm{A}_R} = \frac{\varepsilon^{\mathrm{LCP},\lambda}_{\mathrm{A}_R} - \varepsilon^{\mathrm{RCP},\lambda}_{\mathrm{A}_R}}{\varepsilon^{\lambda}_{\mathrm{A}_R}} \tag{1}$$

where, e.g., $\varepsilon^{\mathrm{LCP},\lambda}_{\mathrm{A}_R}$ is the extinction coefficient of pure the (R)-enantiomer of A towards pure left handed CP light at wavelength, λ and

$$\varepsilon^{\lambda}_{\mathrm{A}_R} = \frac{\varepsilon^{\mathrm{LCP},\lambda}_{\mathrm{A}_R} + \varepsilon^{\mathrm{RCP},\lambda}_{\mathrm{A}_R}}{2} \tag{2}$$

which is equal to the extinction coefficient of the racemate ($\varepsilon^{\lambda}_{\mathrm{A}}$) or of a single enantiomer with plane polarized or unpolarized light. A positive sign for the g-factor indicates that the enantiomer preferentially absorbs LCP light and a negative sign indicates preferential absorbance of RCP light at wavelength λ. It follows from symmetry that

$$g^{\lambda}_{\mathrm{A}_S} = \frac{\varepsilon^{\mathrm{LCP},\lambda}_{\mathrm{A}_S} - \varepsilon^{\mathrm{RCP},\lambda}_{\mathrm{A}_S}}{\varepsilon^{\lambda}_{\mathrm{A}_S}} = -g^{\lambda}_{\mathrm{A}_R} \tag{3}$$

and that

$$\varepsilon_{\mathrm{A}_R}^{\mathrm{LCP},\lambda} = \varepsilon_{\mathrm{A}_S}^{\mathrm{RCP},\lambda} \tag{4}$$
$$\varepsilon_{\mathrm{A}_S}^{\mathrm{LCP},\lambda} = \varepsilon_{\mathrm{A}_R}^{\mathrm{RCP},\lambda} \tag{5}$$

For the work described here involving selective excitation of a single enantiomer from a scalemic or racemic mixture, it is perhaps more useful to consider the g-factor in terms of the difference in extinction coefficients between both enantiomers towards light of a fixed circular polarization. Substituting equation (5) into equation (1) gives

$$g_{\mathrm{A}_R}^{\lambda} = \frac{\varepsilon_{\mathrm{A}_R}^{\mathrm{LCP},\lambda} - \varepsilon_{\mathrm{A}_S}^{\mathrm{LCP},\lambda}}{\varepsilon_{\mathrm{A}}^{\lambda}} \tag{6}$$

showing that a positive value of $g_{\mathrm{A}_R}^{\lambda}$ (which could be estimated by TDDFT calculations on the (R)-enantiomer) indicates that LCP at wavelength λ will preferentially excite the (R)-enantiomer, while a negative $g_{\mathrm{A}_R}^{\lambda}$ indicates LCP at wavelength λ will preferentially excite the (S)-enantiomer. As a result of the sign convention in the definition of the g-factor, the modelling of the asymmetry in the CP light photochemistry is done assuming LCP radiation, unless otherwise stated. If RCP light is used, the sign of the result needs to be inverted.

Where a g-factor for a compound is mentioned without specification of the enantiomer to which it is assigned, this refers to the magnitude of the g-factor only and will vary, e.g., $g_{\mathrm{A}}^{\lambda} = |g_{\mathrm{A}_R}^{\lambda}| = |g_{\mathrm{A}_S}^{\lambda}|$ where $0 < g_{\mathrm{A}}^{\lambda} < 2$.

S1.3 Enantiomeric Excess

Enatiomeric excess, ee, is used throughout this work (as opposed to enantiomeric ratio) because of the simplicity of derivation of the predicted ee from the g-factors. It is presented as a fraction ($-1 \leq ee \leq +1$) rather than a percentage to be more consistent with the presentation of g-factors. For a compound, A, existing as two enantiomers, A_R and A_S, the enantiomeric excess of the A_R enantiomer is defined as:

$$ee_{\mathrm{A}_R} = \frac{[\mathrm{A}_R] - [\mathrm{A}_S]}{[\mathrm{A}_R] + [\mathrm{A}_S]} \tag{7}$$

where a negative value of ee_{A_R} indicates that enantiomer A_S is present in excess. As for the g-factors, an ee referring only to a compound and not a specific enantiomer, e.g., ee_{A} refers to the magnitude of the ee only.

S2 Kinetic Modelling of CP Light Photostationary States

Various aspects of the derivations presented here have been shown elsewhere,[2] especially with regard to the single wavelength kinetics (Section S2.2) and photostationary states (Section S2.3). However, the presentation of the derivations required here is spread across many papers. In order to improve the clarity of this discussion, we present the entire derivation of this model mathematically here in Section S2 before giving the predictions derived from it in Section S4 and in the main text. We do not claim any of the basic concepts presented here to be novel, however, to the best of our knowledge, the application of these concepts to dual wavelength CP light photostationary states has not been previously reported.

S2.1 System under Investigation

In the system of interest, starting material *cis*-**1** exists in rapidly equilibrating enantiomeric conformations, (P)−**1a** and (M)−**1a**, among others. Excitation of one enantiomeric conformation of **1** with 355 nm light forms a specific enantiomer of intermediate **2** and, hence, a specific enantiomer of product **3** assuming no excited state racemization. 355 nm light also promotes the re-opening of the enantiomers of intermediate **2** back to **1** , establishing a single wavelength photostationary state **if** the rate of the photoswitching reactions is much faster than the thermal helix inversion of intermediate **2** to final product **3**. Introduction of 532 nm light further promotes the ring opening of intermediate **2** to starting material **1** and does not affect the ring closing (**1** does not significantly absorb at 532 nm). The models are derived for a general case, then applied specifically to this case.

$$
\begin{array}{ccccc}
(P)-\mathbf{1a} & \underset{355\,+\,532}{\overset{355}{\rightleftharpoons}} & (P,R,R)-\mathbf{2} & \xrightarrow{k_{inv}} & (M,R,R)-\mathbf{3} \\
k_{rac}\Big\updownarrow & & & & \\
(M)-\mathbf{1a} & \underset{355\,+\,532}{\overset{355}{\rightleftharpoons}} & (M,S,S)-\mathbf{2} & \xrightarrow{k_{inv}} & (P,S,S)-\mathbf{3}
\end{array}
$$

To model the photostationary states, we need to establish rate expressions for the photochemical steps, ideally ones that follow simple kinetic rate laws. These can be used to define the positions of the PSSs, accounting for the Kuhn anisotropy factor (g-factor, see equation(1)) to model the enantioselectivity in that PSS. Ultimately, this can be used to predict the enantioselectivity in final product **3** under various conditions.

S2.2 Rate of Photochemical Reactions

S2.2.1 Assumptions

1. The solution is rapidly stirred so that all species are distributed evenly throughout the sample.
2. The Beer-Lambert Law holds at all times during the irradiation.
3. All steps involving deactivation of the excited state are (pseudo-)first order.

S2.2.2 Simple Photochemical Reaction

Species A absorbs light (wavelength, λ) to give an excited state, A*, that can proceed to give product, B, or return to the ground state without chemical change either radiatively of non-radiatively.

$$\mathrm{A} \underset{k_{rad}+k_{non}}{\overset{\lambda}{\rightleftarrows}} \mathrm{A}^* \xrightarrow{k_\mathrm{B}} \mathrm{B}$$

The quantum yield of B on excitation of A, $\Phi_{\mathrm{B}\leftarrow\mathrm{A}}$, defined as the number of molecules of B formed as a fraction of the photons absorbed by A, is given by

$$\Phi_{\mathrm{B}\leftarrow\mathrm{A}} = \frac{k_\mathrm{B}}{\sum k} \tag{8}$$

where $\sum k$ is the sum of all rate constants from A*. For photon fluxes,[3] q_{in} (incident on the sample) and q_{out} (transmitted though the sample), the rate of photons absorbed by the sample, hence the rate, R, of molecular excitation of A in the sample is

$$R_{\mathrm{A}\rightarrow\mathrm{A}^*} = q_{in} - q_{out} \tag{9}$$

Dividing by the volume, V, we get the concentration rate that corresponds to thermal rates (measured in mol dm^{-3} s^{-1}).

$$r_{\mathrm{A}\rightarrow\mathrm{A}^*} = \frac{q_{in} - q_{out}}{V} \tag{10}$$

where q_{in} and q_{out} are related by the Beer-Lambert law, assuming that only A absorbs the light

$$\log_{10}\left(\frac{q_{in}}{q_{out}}\right) = \mathrm{Abs}_\mathrm{A} = \varepsilon_\mathrm{A}[\mathrm{A}]\ell \tag{11}$$

where Abs_A, ε_A and [A] are the absorbance, extinction coefficient and concentration of species A, and ℓ is the path-length of the sample.

Rearranging equation (11) and substitution into (10) gives

$$r_{\mathrm{A}\rightarrow\mathrm{A}^*} = \frac{q_{in}}{V}\left(1 - 10^{-\varepsilon_\mathrm{A}[\mathrm{A}]\ell}\right) \tag{12}$$

and, from the definition of quantum yield of B (equation (8)) on excitation of A ($\Phi_{\mathrm{B}\leftarrow\mathrm{A}}$) as the fraction of excited state of A that goes on to form B, the rate of formation of the photoproduct B,

$$r_{\mathrm{A}\rightarrow\mathrm{B}} = \frac{q_{in}\Phi_{\mathrm{B}\leftarrow\mathrm{A}}}{V}\left(1 - 10^{-\varepsilon_\mathrm{A}[\mathrm{A}]\ell}\right) \tag{13}$$

Generally, the kinetics of a basic photochemical reaction do not give a simple rate law. However, in the limits of high or low absorbance, this can be simplified, as outlined below.

S2.2.3 Rate Law at High Absorbance

When the absorbance ($\varepsilon_\mathrm{A}[\mathrm{A}]\ell$) is large, $10^{-\varepsilon_\mathrm{A}[\mathrm{A}]\ell} \ll 1$ so equation (13) simplifies to

$$r_{\mathrm{A}\rightarrow\mathrm{B}} = \frac{q_{in}\Phi_{\mathrm{B}\leftarrow\mathrm{A}}}{V} \tag{14}$$

and the photochemical reaction exhibits zeroth-order kinetics (rate independent of concentration).

S2.2.4 Rate Law at Low Absorbance

Given

$$10^{-\varepsilon[\mathrm{A}]\ell} = e^{-\varepsilon[\mathrm{A}]\ell \ln 10} \tag{15}$$

and the Taylor expansion

$$e^{-\varepsilon_{\mathrm{A}}[\mathrm{A}]\ell \ln 10} = 1 - \varepsilon_{\mathrm{A}}[\mathrm{A}]\ell \ln 10 + \frac{(\varepsilon_{\mathrm{A}}[\mathrm{A}]\ell \ln 10)^2}{2!} - \frac{(\varepsilon_{\mathrm{A}}[\mathrm{A}]\ell \ln 10)^3}{3!} + ... \tag{16}$$

when $\varepsilon_{\mathrm{A}}[\mathrm{A}]\ell \ln 10 \ll 1$ (hence $\mathrm{Abs_A} \ll 0.43$), the Taylor series can be truncated at the first-order term, simplifying equation (13) to

$$r_{\mathrm{A}\to\mathrm{B}} \approx \frac{q_{in}\Phi_{\mathrm{B}\leftarrow\mathrm{A}}}{V}\varepsilon_{\mathrm{A}}[\mathrm{A}]\ell \ln 10 \tag{17}$$

giving an approximate first-order rate constant, $k_{\mathrm{A}\to\mathrm{B}}$, for the simple photochemical process under low absorbance conditions

$$k_{\mathrm{A}\to\mathrm{B}} \approx \frac{q_{in}\Phi_{\mathrm{B}\leftarrow\mathrm{A}}}{V}\varepsilon_{\mathrm{A}}\ell \ln 10 \tag{18}$$

S2.2.5 Rate Laws with Two or More Absorbing Species

When two (or more) absorbing species are present, the fraction of the light absorbed by component A (in an A + M mixture) is given by the absorbance due to component A divided by the total sample absorbance. Hence for the scheme:

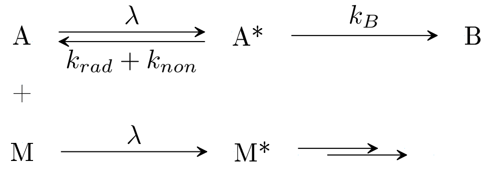

$$r_{\mathrm{A}\to\mathrm{B}} = \frac{\varepsilon_{\mathrm{A}}[\mathrm{A}]}{\varepsilon_{\mathrm{A}}[\mathrm{A}] + \varepsilon_{\mathrm{M}}[\mathrm{M}]}\frac{q_{in}\Phi_{\mathrm{B}\leftarrow\mathrm{A}}}{V}\left(1 - 10^{-(\varepsilon_{\mathrm{A}}[\mathrm{A}]+\varepsilon_{\mathrm{M}}[\mathrm{M}])\ell}\right) \tag{19}$$

or for any number of species, i

$$r_{\mathrm{A}\to\mathrm{B}} = \frac{\varepsilon_{\mathrm{A}}[\mathrm{A}]}{\sum \varepsilon_i[i]}\frac{q_{in}\Phi_{\mathrm{B}\leftarrow\mathrm{A}}}{V}\left(1 - 10^{-\sum \varepsilon_i[i]\ell}\right) \tag{20}$$

Under high absorbance conditions, this gives

$$r_{\mathrm{A}\to\mathrm{B}} = \frac{\mathrm{Abs_A}}{\mathrm{Abs_{total}}}\frac{q_{in}\Phi_{\mathrm{B}\leftarrow\mathrm{A}}}{V} \tag{21}$$

so the rate will vary with concentration of other species in the reaction.

Under the low absorbance conditions, the truncated Taylor expansion gives (now providing that the **total** sample absorbance $A_{\mathrm{total}} \ll 0.43$ at the wavelength of irradiation).

$$r_{\mathrm{A}\to\mathrm{B}} \approx \frac{\varepsilon_{\mathrm{A}}[\mathrm{A}]}{\cancel{\sum \varepsilon_i[i]}}\frac{q_{in}\Phi_{\mathrm{B}\leftarrow\mathrm{A}}}{V}\left(\cancel{\sum \varepsilon_i[i]}\right)\ell \ln 10 \tag{22}$$

Which, after cancelling, is identical to equation (17) for the rate under low absorbance conditions for a single absorbing species. Low absorbance species do not affect the photochemical reaction rates of the other species simplifying the analysis. Where low absorbance is assumed, this practically limits the method to low concentrations or short path-lengths and attempts to scale up the results here to synthetically useful levels will probably require short path lengths for the irradiation and the use of flow chemistry.[4]

S2.2.6 Simple Enantioselective Photosynthesis using CP light

In the simplest asymmetric photosynthesis, a starting material, A, exists in rapidly equilibrating enantiomeric conformers, A_R and A_S, that can be selectively excited by CP light before stereospecifically forming photostable, non-interconverting enantiomers B_R and B_S respectively.

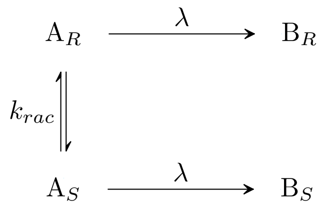

Using the general equation (20) for multiple absorbing species and assuming that no excited state racemization occurs

$$\frac{\frac{\mathrm{d}[\mathrm{B}_R]}{\mathrm{d}t}}{\frac{\mathrm{d}[\mathrm{B}_S]}{\mathrm{d}t}} = \frac{\frac{\varepsilon_{\mathrm{A}_R}[\mathrm{A}_R]}{\sum \varepsilon_i[i]} \frac{q_{in}\Phi_{\mathrm{B}_R\leftarrow\mathrm{A}_R}}{V}\left(1-10^{-\sum \varepsilon_i[i]\ell}\right)}{\frac{\varepsilon_{\mathrm{A}_S}[\mathrm{A}_S]}{\sum \varepsilon_i[i]} \frac{q_{in}\Phi_{\mathrm{B}_S\leftarrow\mathrm{A}_S}}{V}\left(1-10^{-\sum \varepsilon_i[i]\ell}\right)} \tag{23}$$

Provided that the racemization of A is faster than the photochemical conversion of either enantiomer of A to B, i.e., $k_{rac}[\mathrm{A}_R] \gg r_{\mathrm{A}_R\rightarrow\mathrm{B}_R}$ and $k_{rac}[\mathrm{A}_S] \gg r_{\mathrm{A}_S\rightarrow\mathrm{B}_S}$, $[\mathrm{A}_R] = [\mathrm{A}_S]$ at any time. Since the quantum yield depends only on thermal rate constants from the excited state (equal for each enantiomer of a given species), $\Phi_{\mathrm{B}_R\leftarrow\mathrm{A}_R} = \Phi_{\mathrm{B}_S\leftarrow\mathrm{A}_S} = \Phi_{\mathrm{B}\leftarrow\mathrm{A}}$, so equation(23) simplifies to

$$\frac{\frac{\mathrm{d}[\mathrm{B}_R]}{\mathrm{d}t}}{\frac{\mathrm{d}[\mathrm{B}_S]}{\mathrm{d}t}} = \frac{\varepsilon_{\mathrm{A}_R}}{\varepsilon_{\mathrm{A}_S}} \tag{24}$$

hence, at any time during the reaction

$$\frac{[\mathrm{B}_R]}{[\mathrm{B}_S]} = \frac{\varepsilon_{\mathrm{A}_R}}{\varepsilon_{\mathrm{A}_S}} \tag{25}$$

From the definition of *ee*

$$ee_{\mathrm{B}_R} = \frac{[\mathrm{B}_R]-[\mathrm{B}_S]}{[\mathrm{B}_R]+[\mathrm{B}_S]} \tag{26}$$

$$ee_{\mathrm{B}_R} = \frac{\varepsilon_{\mathrm{A}_R}-\varepsilon_{\mathrm{A}_S}}{\varepsilon_{\mathrm{A}_R}+\varepsilon_{\mathrm{A}_S}} \tag{27}$$

Then comparing to the definition of the g-factor (equations (1) and (2))

$$g_{\mathrm{A}_R} = \frac{2\left(\varepsilon_{\mathrm{A}_R}-\varepsilon_{\mathrm{A}_S}\right)}{\varepsilon_{\mathrm{A}_R}+\varepsilon_{\mathrm{A}_S}} \tag{28}$$

$$ee_{\mathrm{B}_R} = \frac{g_{\mathrm{A}_R}}{2} \tag{29}$$

When exciting enantiomeric conformations of a single molecule that forms a product that does not undergo photochemical transformations, the expected *ee* of the product is half the g-factor of the starting material, under the conditions outlined above. This result has been reported in a variety of reviews on asymmetric photochemistry.[5]

S2.3 Single Wavelength Enantioselective Photostationary States

S2.3.1 Simple Photostationary State (PSS)

At a photostationary state between species A and B in which both absorb light at one wavelength and are photochemically interconverted, the rates of both reactions must balance.

$$\mathrm{A} \underset{\lambda}{\overset{\lambda}{\rightleftarrows}} \mathrm{B}$$

$$r_{\mathrm{A}\to\mathrm{B}} = r_{\mathrm{B}\to\mathrm{A}} \tag{30}$$

In the general case, applying equation (19) gives:[6]

$$\frac{\varepsilon_{\mathrm{A}}[\mathrm{A}]}{\varepsilon_{\mathrm{A}}[\mathrm{A}]+\varepsilon_{\mathrm{B}}[\mathrm{B}]}\frac{q_{in}\Phi_{\mathrm{B}\leftarrow\mathrm{A}}}{V}\left(1-10^{-(\varepsilon_{\mathrm{A}}[\mathrm{A}]+\varepsilon_{\mathrm{B}}[\mathrm{B}])\ell}\right) = \frac{\varepsilon_{\mathrm{B}}[\mathrm{B}]}{\varepsilon_{\mathrm{A}}[\mathrm{A}]+\varepsilon_{\mathrm{B}}[\mathrm{B}]}\frac{q_{in}\Phi_{\mathrm{A}\leftarrow\mathrm{B}}}{V}\left(1-10^{-(\varepsilon_{\mathrm{A}}[\mathrm{A}]+\varepsilon_{\mathrm{B}}[\mathrm{B}])\ell}\right) \tag{31}$$

$$\varepsilon_{\mathrm{A}}[\mathrm{A}]\Phi_{\mathrm{B}\leftarrow\mathrm{A}} = \varepsilon_{\mathrm{B}}[\mathrm{B}]\Phi_{\mathrm{A}\leftarrow\mathrm{B}} \tag{32}$$

$$\frac{[\mathrm{B}]}{[\mathrm{A}]} = \frac{\varepsilon_{\mathrm{A}}\Phi_{\mathrm{B}\leftarrow\mathrm{A}}}{\varepsilon_{\mathrm{B}}\Phi_{\mathrm{A}\leftarrow\mathrm{B}}} \tag{33}$$

The low absorbance approximation is not required in this case.

S2.3.2 Asymmetric Photosynthetic PSS

For rapidly equilibrating enantiomeric conformations, A_R and A_S, each in a photostationary state with non-interconverting enantiomeric products, B_R and B_S respectively, the ratio of the photoproducts at the PSS can be predicted.

$$\begin{array}{ccc} \mathrm{A}_R & \underset{\lambda}{\overset{\lambda}{\rightleftarrows}} & \mathrm{B}_R \\ k_{rac}\Big\updownarrow & & \\ \mathrm{A}_S & \underset{\lambda}{\overset{\lambda}{\rightleftarrows}} & \mathrm{B}_S \end{array}$$

Equation (33) predicts that, at the PSS (given that the quantum yields for the dark reactions of each enantiomer of a species must be equal)

$$\frac{[\mathrm{B}_R]}{[\mathrm{A}_R]} = \frac{\varepsilon_{\mathrm{A}_R}\Phi_{\mathrm{B}\leftarrow\mathrm{A}}}{\varepsilon_{\mathrm{B}_R}\Phi_{\mathrm{A}\leftarrow\mathrm{B}}} \tag{34}$$

$$\frac{[\mathrm{B}_S]}{[\mathrm{A}_S]} = \frac{\varepsilon_{\mathrm{A}_S}\Phi_{\mathrm{B}\leftarrow\mathrm{A}}}{\varepsilon_{\mathrm{B}_S}\Phi_{\mathrm{A}\leftarrow\mathrm{B}}} \tag{35}$$

Assuming that $k_{rac}[\mathrm{A}_R] \gg r_{\mathrm{A}_R\to\mathrm{B}_R}$ and $k_{rac}[\mathrm{A}_S] \gg r_{\mathrm{A}_S\to\mathrm{B}_S}$, then $[\mathrm{A}_R] = [\mathrm{A}_S]$ so, dividing equation (34) by equation (35) gives

$$\frac{[\mathrm{B}_R]}{[\mathrm{B}_S]} = \frac{\varepsilon_{\mathrm{A}_R}\varepsilon_{\mathrm{B}_S}}{\varepsilon_{\mathrm{A}_S}\varepsilon_{\mathrm{B}_R}} \tag{36}$$

Rearranging the definition of the g-factor for A_R and B_R gives

$$\frac{\varepsilon_{\mathrm{A}_R}}{\varepsilon_{\mathrm{A}_S}} = \frac{2+g_{\mathrm{A}_R}}{2-g_{\mathrm{A}_R}} \tag{37}$$

$$\frac{\varepsilon_{\mathrm{B}_S}}{\varepsilon_{\mathrm{B}_R}} = \frac{2-g_{\mathrm{B}_R}}{2+g_{\mathrm{B}_R}} \tag{38}$$

Substituting equations (37) and (38) into equation (36) and rearranging gives

$$\frac{[\mathrm{B}_R]}{[\mathrm{B}_S]} = \frac{(2+g_{\mathrm{A}_R})(2-g_{\mathrm{B}_R})}{(2-g_{\mathrm{A}_R})(2+g_{\mathrm{B}_R})} \tag{39}$$

Rearranging the definition of ee (equation (7))

$$ee_{\mathrm{B}_R} = \frac{\frac{[\mathrm{B}_R]}{[\mathrm{B}_S]}-1}{\frac{[\mathrm{B}_R]}{[\mathrm{B}_S]}+1} \tag{40}$$

Substituting equation (39) into equation (40) and rearranging gives

$$ee_{\mathrm{B}_R} = \frac{2g_{\mathrm{A}_R}-2g_{\mathrm{B}_R}}{4-g_{\mathrm{A}_R}g_{\mathrm{B}_R}} \tag{41}$$

For the π–π^* transitions in these systems, $g_{\mathrm{A}_R}g_{\mathrm{B}_R} \ll 4$, so

$$ee_{\mathrm{B}_R} \approx \frac{g_{\mathrm{A}_R}-g_{\mathrm{B}_R}}{2} \tag{42}$$

where the minus sign indicates that the enantioselective effect of the single wavelength of CP light on the forward and backward photochemical steps will be additive only if the sign of the g-factor at wavelength, λ, of B_R is the **opposite** to that of A_R, i.e., when the CP light-induced back reaction selectively destroys the minor product of the CP light-induced forward reaction. The relative signs of these two g-factors are intrinsic properties of the molecules and cannot be varied. Of course, the handedness of the CP light at the single wavelength must be the same for inducing both forward and back reactions.

The initiation of the back reaction moves the PSS towards the starting material, so any increase in ee must come at the cost of product yield compared to initiating the forward reaction alone. If either of the g-factors are zero, the ee is determined solely by the g-factor of the other species. An example where the forward reaction must have a zero g-factor is where the starting material, A, does not exist in enantiomeric conformations but, on irradiation, forms a chiral product, B which can be converted back to A photochemically. No selectivity is possible in the forward reaction, but, under the correct conditions, the product can be selectively converted back to starting material with CP light giving enantiomerically enriched B in the PSS.

S2.4 Dual Wavelength CP Light Photochemistry

S2.4.1 Rate and PSS for Dual Wavelength Photochemistry

Under the assumptions made in Section S2.2.1, the rate of conversion of A→B induced by light of two wavelengths, λ_1 and λ_2, is the sum of the rates induced by each of the wavelengths separately.[7]

$$\mathrm{A} \xrightarrow{\lambda_1 + \lambda_2} \mathrm{B}$$

$$r^{\lambda_1+\lambda_2}_{\mathrm{A}\to\mathrm{B}} = r^{\lambda_1}_{\mathrm{A}\to\mathrm{B}} + r^{\lambda_2}_{\mathrm{A}\to\mathrm{B}} \tag{43}$$

At the photo stationary state,

$$\mathrm{A} \underset{\lambda_1 + \lambda_2}{\overset{\lambda_1 + \lambda_2}{\rightleftarrows}} \mathrm{B}$$

$$r^{\lambda_1}_{\mathrm{A}\to\mathrm{B}} + r^{\lambda_2}_{\mathrm{A}\to\mathrm{B}} = r^{\lambda_1}_{\mathrm{B}\to\mathrm{A}} + r^{\lambda_2}_{\mathrm{B}\to\mathrm{A}} \tag{44}$$

S2.4.2 Enantioselectivity in a Photochemical Step Initiated by Two Wavelengths

$$\begin{array}{ccc} \mathrm{A}_R & \xrightarrow{\lambda_1 + \lambda_2} & \mathrm{B}_R \\ k_{rac}\Big\updownarrow & & \\ \mathrm{A}_S & \xrightarrow{\lambda_1 + \lambda_2} & \mathrm{B}_S \end{array}$$

Using equation (43), the low absorbance approximation from equation (17) and the fact that the quantum yields in each enantiomeric reaction must be equal (but the quantum yields for the same reaction initiated by different wavelengths need not be),

$$\frac{\frac{\mathrm{d}[\mathrm{B}_R]}{\mathrm{d}t}}{\frac{\mathrm{d}[\mathrm{B}_S]}{\mathrm{d}t}} = \frac{\frac{q^{\lambda_1}_{in}\Phi^{\lambda_1}_{\mathrm{B}\leftarrow\mathrm{A}}}{V}\varepsilon^{\lambda_1}_{\mathrm{A_R}}\ell \ln 10[\mathrm{A}_R] + \frac{q^{\lambda_2}_{in}\Phi^{\lambda_2}_{\mathrm{B}\leftarrow\mathrm{A}}}{V}\varepsilon^{\lambda_2}_{\mathrm{A_R}}\ell \ln 10[\mathrm{A}_R]}{\frac{q^{\lambda_1}_{in}\Phi^{\lambda_1}_{\mathrm{B}\leftarrow\mathrm{A}}}{V}\varepsilon^{\lambda_1}_{\mathrm{A_S}}\ell \ln 10[\mathrm{A}_S] + \frac{q^{\lambda_2}_{in}\Phi^{\lambda_2}_{\mathrm{B}\leftarrow\mathrm{A}}}{V}\varepsilon^{\lambda_2}_{\mathrm{A_S}}\ell \ln 10[\mathrm{A}_S]} \tag{45}$$

With fast equilibration of the enantiomers of A, $[\mathrm{A}_R] = [\mathrm{A}_S]$, so the rate ratio is time-independant and, at any time,

$$\frac{[\mathrm{B}_R]}{[\mathrm{B}_S]} = \frac{q^{\lambda_1}_{in}\Phi^{\lambda_1}_{\mathrm{B}\leftarrow\mathrm{A}}\varepsilon^{\lambda_1}_{\mathrm{A}_R} + q^{\lambda_2}_{in}\Phi^{\lambda_2}_{\mathrm{B}\leftarrow\mathrm{A}}\varepsilon^{\lambda_2}_{\mathrm{A}_R}}{q^{\lambda_1}_{in}\Phi^{\lambda_1}_{\mathrm{B}\leftarrow\mathrm{A}}\varepsilon^{\lambda_1}_{\mathrm{A}_S} + q^{\lambda_2}_{in}\Phi^{\lambda_2}_{\mathrm{B}\leftarrow\mathrm{A}}\varepsilon^{\lambda_2}_{\mathrm{A}_S}} \tag{46}$$

Dividing top and bottom by $q^{\lambda_2}_{in}\Phi_{\mathrm{B}\leftarrow\mathrm{A}}$ gives

$$\frac{[\mathrm{B}_R]}{[\mathrm{B}_S]} = \frac{c\varepsilon^{\lambda_1}_{\mathrm{A}_R} + \varepsilon^{\lambda_2}_{\mathrm{A}_R}}{c\varepsilon^{\lambda_1}_{\mathrm{A}_S} + \varepsilon^{\lambda_2}_{\mathrm{A}_S}} \tag{47}$$

where $c = \frac{q^{\lambda_1}_{in}\Phi^{\lambda_1}_{\mathrm{B}\leftarrow\mathrm{A}}}{q^{\lambda_2}_{in}\Phi^{\lambda_2}_{\mathrm{B}\leftarrow\mathrm{A}}}$ (dependant only on the relative photon flux between the two wavelengths for any individual reaction) varies between ∞ (λ_1 dominates the reaction) and 0 (λ_2 dominates the reaction). From the definition of ee in equation (40)

$$ee_{\mathrm{B}_R} = \frac{c\varepsilon^{\lambda_1}_{\mathrm{A}_R} + \varepsilon^{\lambda_2}_{\mathrm{A}_R} - c\varepsilon^{\lambda_1}_{\mathrm{A}_S} - \varepsilon^{\lambda_2}_{\mathrm{A}_S}}{c\varepsilon^{\lambda_1}_{\mathrm{A}_R} + \varepsilon^{\lambda_2}_{\mathrm{A}_R} + c\varepsilon^{\lambda_1}_{\mathrm{A}_S} + \varepsilon^{\lambda_2}_{\mathrm{A}_S}} \tag{48}$$

$$ee_{\mathrm{B}_R} = \frac{c\Delta\varepsilon^{\lambda_1}_{\mathrm{A}} + \Delta\varepsilon^{\lambda_2}_{\mathrm{A}}}{2\left(c\varepsilon^{\lambda_1}_{\mathrm{A}} + \varepsilon^{\lambda_2}_{\mathrm{A}}\right)} \tag{49}$$

where $\Delta\varepsilon_{\mathrm{A}} = \varepsilon_{\mathrm{A}_R} - \varepsilon_{\mathrm{A}_S}$ and $2\varepsilon_{\mathrm{A}} = \varepsilon_{\mathrm{A}_R} + \varepsilon_{\mathrm{A}_S}$. This expression has no stationary points for $0 < c < \infty$. The maximum ee will be obtained either when $c = \infty$, $ee = 0.5g^{\lambda_1}_{\mathrm{A}}$ or when $c = 0$, $ee = 0.5g^{\lambda_2}_{\mathrm{A}}$ depending on which wavelength has the greatest g-factor. The dual wavelength photochemistry should **not** be able to increase the enantioselectivity of a simple asymmetric photosynthesis (or for an individual photochemical step in a PSS in a more complex mechanism). A similar analysis shows this also to be the case for the selectivity factor in an asymmetric photodestruction.

S2.4.3 Dual-Wavelength CP Light PSS

For rapidly equilibrating enantiomeric conformations, A_R and A_S, irradiated at wavelengths, λ_1 and λ_2, to establish photostationary states with B_R and B_S respectively:

$$\begin{array}{ccc} \mathrm{A}_R & \underset{\lambda_1+\lambda_2}{\overset{\lambda_1+\lambda_2}{\rightleftharpoons}} & \mathrm{B}_R \\ k_{rac} \Big\Updownarrow & & \\ \mathrm{A}_S & \underset{\lambda_1+\lambda_2}{\overset{\lambda_1+\lambda_2}{\rightleftharpoons}} & \mathrm{B}_S \end{array}$$

Under the low absorbance approximation (equation (17)) the reactions follow first-order kinetics so, at the photostationary state,

$$\frac{[\mathrm{B}_R]}{[\mathrm{A}_R]} = \frac{k^{\lambda_1}_{\mathrm{A}_R\to\mathrm{B}_R} + k^{\lambda_2}_{\mathrm{A}_R\to\mathrm{B}_R}}{k^{\lambda_1}_{\mathrm{B}_R\to\mathrm{A}_R} + k^{\lambda_2}_{\mathrm{B}_R\to\mathrm{A}_R}} \tag{50}$$

$$\frac{[\mathrm{B}_S]}{[\mathrm{A}_S]} = \frac{k^{\lambda_1}_{\mathrm{A}_S\to\mathrm{B}_S} + k^{\lambda_2}_{\mathrm{A}_S\to\mathrm{B}_S}}{k^{\lambda_1}_{\mathrm{B}_S\to\mathrm{A}_S} + k^{\lambda_2}_{\mathrm{B}_S\to\mathrm{A}_S}} \tag{51}$$

Assuming rapid equilibration of the enantiomers of A, $[\mathrm{A}_R] = [\mathrm{A}_S]$, so dividing equation (50) by equation (51) gives

$$\frac{[\mathrm{B}_R]}{[\mathrm{B}_S]} = \frac{\left(k^{\lambda_1}_{\mathrm{A}_R\to\mathrm{B}_R} + k^{\lambda_2}_{\mathrm{A}_R\to\mathrm{B}_R}\right)\left(k^{\lambda_1}_{\mathrm{B}_S\to\mathrm{A}_S} + k^{\lambda_2}_{\mathrm{B}_S\to\mathrm{A}_S}\right)}{\left(k^{\lambda_1}_{\mathrm{B}_R\to\mathrm{A}_R} + k^{\lambda_2}_{\mathrm{B}_R\to\mathrm{A}_R}\right)\left(k^{\lambda_1}_{\mathrm{A}_S\to\mathrm{B}_S} + k^{\lambda_2}_{\mathrm{A}_S\to\mathrm{B}_S}\right)} \tag{52}$$

This leads to complicated expressions for the *ee* so, instead of studying the full expression, some simplifications can be made that are relevant to the reaction and wavelengths under investigation. Firstly, if λ_2 is chosen such that it is negligibly absorbed by the starting material (as is the case for 532 nm and *cis*-dinaphthylethene **1**), $k^{\lambda_2}_{\mathrm{A}_R\to\mathrm{B}_R} \approx 0$ and $k^{\lambda_2}_{\mathrm{A}_S\to\mathrm{B}_S} \approx 0$ so

$$\frac{[\mathrm{B}_R]}{[\mathrm{B}_S]} = \frac{k^{\lambda_1}_{\mathrm{A}_R\to\mathrm{B}_R}\left(k^{\lambda_1}_{\mathrm{B}_S\to\mathrm{A}_S} + k^{\lambda_2}_{\mathrm{B}_S\to\mathrm{A}_S}\right)}{k^{\lambda_1}_{\mathrm{A}_S\to\mathrm{B}_S}\left(k^{\lambda_1}_{\mathrm{B}_R\to\mathrm{A}_R} + k^{\lambda_2}_{\mathrm{B}_R\to\mathrm{A}_R}\right)} \tag{53}$$

Now, considering the extreme case of low power λ_2, $k^{\lambda_2}_{\mathrm{B}\to\mathrm{A}} \approx 0$, so

$$\frac{[\mathrm{B}_R]}{[\mathrm{B}_S]} = \frac{k^{\lambda_1}_{\mathrm{A}_R\to\mathrm{B}_R} k^{\lambda_1}_{\mathrm{B}_S\to\mathrm{A}_S}}{k^{\lambda_1}_{\mathrm{A}_S\to\mathrm{B}_S} k^{\lambda_1}_{\mathrm{B}_R\to\mathrm{A}_R}} \tag{54}$$

which, ultimately and expectedly, reduces to the same expression for *ee* as in the single wavelength PSS, so

$$ee_{\mathrm{B}_R} \approx \frac{g^{\lambda_1}_{\mathrm{A}_R} - g^{\lambda_1}_{\mathrm{B}_R}}{2} \tag{55}$$

again, where the minus sign indicates that the *ee* will be increased over that from the forward reaction alone only if the signs of the *g*-factors for the starting material and product at λ_1 are opposite.

Considering the other extreme, the case of very high power λ_2, $k^{\lambda_2}_{\mathrm{B}\to\mathrm{A}} \gg k^{\lambda_1}_{\mathrm{B}\to\mathrm{A}}$ so equation (53) becomes

$$\frac{[\mathrm{B}_R]}{[\mathrm{B}_S]} = \frac{k^{\lambda_1}_{\mathrm{A}_R\to\mathrm{B}_R} k^{\lambda_2}_{\mathrm{B}_S\to\mathrm{A}_S}}{k^{\lambda_1}_{\mathrm{A}_S\to\mathrm{B}_S} k^{\lambda_2}_{\mathrm{B}_R\to\mathrm{A}_R}} \tag{56}$$

Substitution for the low absorbance rate constants (equation (18)) and cancelling where possible gives

$$\frac{[\mathrm{B}_R]}{[\mathrm{B}_S]} = \frac{\varepsilon^{\lambda_1}_{\mathrm{A}_R}\varepsilon^{\lambda_2}_{\mathrm{B}_S}}{\varepsilon^{\lambda_1}_{\mathrm{A}_S}\varepsilon^{\lambda_2}_{\mathrm{B}_R}} \tag{57}$$

Taking the usual definitions of $g^{\lambda_1}_{\mathrm{A}_R}$ and $g^{\lambda_1}_{\mathrm{B}_R}$ but now, using the $\pm$ or $\mp$ symbols, we account for the fact that now the handedness of the second wavelength can be either the same (LCP, top symbol) or opposite (RCP, bottom symbol) to that of the first (LCP), we now get

$$\frac{\varepsilon^{\lambda_1}_{\mathrm{A}_R}}{\varepsilon^{\lambda_1}_{\mathrm{A}_S}} = \frac{2 + g^{\lambda_1}_{\mathrm{A}_R}}{2 - g^{\lambda_1}_{\mathrm{A}_R}} \tag{58}$$

$$\frac{\varepsilon^{\lambda_2}_{\mathrm{B}_S}}{\varepsilon^{\lambda_2}_{\mathrm{B}_R}} = \frac{2 \mp g^{\lambda_2}_{\mathrm{B}_R}}{2 \pm g^{\lambda_2}_{\mathrm{B}_R}} \tag{59}$$

Substituting equations (58) and (59) into equation (57) and proceeding as in Section S2.3.2 yields

$$ee_{\mathrm{B}_R} = \frac{2g^{\lambda_1}_{\mathrm{A}_R} \mp 2g^{\lambda_2}_{\mathrm{B}_R}}{4 - g^{\lambda_1}_{\mathrm{A}_R} g^{\lambda_2}_{\mathrm{B}_R}} \tag{60}$$

For the π–π^* transitions in unsaturated hydrocarbons (such as those under study here), $g_{\mathrm{A}_R} g_{\mathrm{B}_R} \ll 4$, so

$$ee_{\mathrm{B}_R} \approx \frac{g^{\lambda_1}_{\mathrm{A}_R} \mp g^{\lambda_2}_{\mathrm{B}_R}}{2} \tag{61}$$

where the $\mp$ sign shows that:

- If $g^{\lambda_1}_{\mathrm{A}_R}$ and $g^{\lambda_2}_{\mathrm{B}_R}$ have the **same** sign, the *ee* effects should be additive using CP light of **opposite** handedness at the two wavelengths.
- If $g^{\lambda_1}_{\mathrm{A}_R}$ and $g^{\lambda_2}_{\mathrm{B}_R}$ have the **opposite** sign, the *ee* effects should be additive using CP light of **same** handedness at the two wavelengths.

Combining these two results, it can be seen that the *ee* of the product in the PSS should change from that of the single wavelength (given by equation (55)) monotonically[8] to that given in equation (61) as the power at the second wavelength is steadily increased. Therefore, the introduction of the second wavelength, with the appropriate circular polarization and sufficient power relative to the first should lead to an increase in the *ee* at the PSS **unless**:

- The magnitude of the g-factor of the product at λ_1 is greater than that at λ_2 **and**
- The sign of the g-factor of the product at λ_1 is the opposite to that of the starting material at λ_1.

Physically this can be interpreted as replacing the selectivity due to $g^{\lambda_1}_{\mathrm{B}_R}$ with that due to $g^{\lambda_2}_{\mathrm{B}_R}$ in the PSS as the power at λ_2 is increased. When plane polarized (or unpolarized) light is used at either wavelength, this effectively sets $g = 0$.

This second wavelength promoting only the back reaction must move the PSS towards A, so this increase in *ee* must come at the cost of product yield.

S2.5 Combined Thermal and Photochemistry

Considering the situation in Section S2.4.3 where now the photoproduct enantiomers B_R and B_S undergoes a stereospecific and irreversible thermal conversion to (photo)stable enantiomers, C_R and C_S respectively, necessarily with the same rate thermal constant, $k^{\Delta}_{B\to C}$. With two wavelengths, this is a generalized version of the specific reaction of interest shown in Section S2.1.

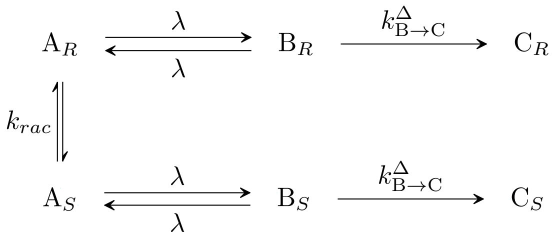

Under low absorbance conditions (Section S2.2.4) the photochemical steps with one or many wavelengths follow first-order kinetics (equations (18) and (43)).

S2.5.1 Enantioselective Synthesis at High Power

At high laser powers, $k^{\lambda}_{B_R\to A_R}, k^{\lambda}_{B_S\to A_S} \gg k^{\Delta}_{B\to C}$. Depending on the position of the PSS between A and B, significant quantities of B many accumulate, so the steady state approximation cannot be applied. However, since the thermal inversion step is unselective ($k^{\Delta}_{B\to C}$ is equal for both enantiomers), the *ee* of product C must reflect the PSS *ee* of B, hence, for a single wavelength PSS (from equation (42), Section S2.3.2) or a dual wavelength PSS where the same wavelength dominates both the forward and reverse reactions (from equation (55), Section S2.4.3)

$$ee_{C_R} \approx \frac{g_{A_R} - g_{B_R}}{2} \tag{62}$$

or for a dual wavelength PSS where different wavelengths dominate the forward and reverse reactions (from equation (61), Section S2.4.3)

$$ee_{C_R} \approx \frac{g^{\lambda_1}_{A_R} \mp g^{\lambda_2}_{B_R}}{2} \tag{63}$$

where the significance of the $\mp$ sign is discussed below equation (61).

The kinetics of formation of C in this case can be determined using:

$$\frac{d[C]}{dt} = k^{\Delta}_{B\to C}[B] \tag{64}$$

with the initial concentration if starting material

$$A_0 = [A] + [B] + [C] \tag{65}$$

and, because the PSS is fully and rapidly established

$$[A] = \frac{k^{\lambda}_{B\to A}}{k^{\lambda}_{A\to B}}[B] \tag{66}$$

Substituting equation (66) into equation (65), then into equation (64) gives

$$\frac{\mathrm{d[C]}}{\mathrm{d}t} = k^{\Delta}_{\mathrm{B\rightarrow C}} \frac{k^{\lambda}_{\mathrm{A\rightarrow B}}}{k^{\lambda}_{\mathrm{A\rightarrow B}} + k^{\lambda}_{\mathrm{B\rightarrow A}}} (\mathrm{A_0} - [\mathrm{C}]) \tag{67}$$

Rearranging and integrating gives

$$[\mathrm{C}] = \mathrm{A_0} \left[1 - \exp\left(-k^{\Delta}_{\mathrm{B\rightarrow C}} \frac{k^{\lambda}_{\mathrm{A\rightarrow B}}}{k^{\lambda}_{\mathrm{A\rightarrow B}} + k^{\lambda}_{\mathrm{B\rightarrow A}}} t\right)\right] \tag{68}$$

showing first order kinetics with an observed rate constant

$$k_{obs} = k^{\Delta}_{\mathrm{B\rightarrow C}} \frac{k^{\lambda}_{\mathrm{A\rightarrow B}}}{k^{\lambda}_{\mathrm{A\rightarrow B}} + k^{\lambda}_{\mathrm{B\rightarrow A}}} \tag{69}$$

which is independent of photon flux and, hence, on laser power. It can also be shown that the fraction $\frac{k^{\lambda}_{\mathrm{A\rightarrow B}}}{k^{\lambda}_{\mathrm{A\rightarrow B}}+k^{\lambda}_{\mathrm{B\rightarrow A}}}$ is equal to the mole fraction of B present in the PSS. Determination of the rate constant at high laser power, and additionally of the thermal rate constant $k^{\Delta}_{\mathrm{B\rightarrow C}}$ provides an estimate of the position of the PSS position at the wavelength if it cannot be easily determined by other means.

S2.5.2 Enantioselective Synthesis at Low Power

At low laser powers, $k^{\lambda}_{\mathrm{B}_R\rightarrow\mathrm{A}_R}, k^{\lambda}_{\mathrm{B}_S\rightarrow\mathrm{A}_S}, k^{\lambda}_{\mathrm{A}_R\rightarrow\mathrm{B}_R}, k^{\lambda}_{\mathrm{A}_S\rightarrow\mathrm{B}_S} \ll k^{\Delta}_{\mathrm{B\rightarrow C}}$ and so B is thermally converted to C as fast as it it formed, hence $[\mathrm{B}] \approx 0$ and the steady state approximation can be applied. Since the thermal reaction is unselective, the *ee* of the final product will reflect only the enantioselectivitiy in the formation of B from A as described for the simple asymmetric photosynthesis by equation (29) in Section S2.2.6. The *ee* of the final product (assuming that only one wavelength is absorbed by the starting material) and kinetics of its formation are expected to be

$$ee_{\mathrm{C}_R} = \frac{g_{\mathrm{A}_R}}{2} \tag{70}$$

$$\frac{\mathrm{d[C]}}{\mathrm{d}t} = k^{\lambda}_{\mathrm{A\rightarrow B}}[\mathrm{A}] \tag{71}$$

The *ee* therefore cannot be improved by a dual wavelength approach above the best single wavelength *ee* available if the thermal reaction trapping the photoproduct is faster that the enantioselective back reaction (see Section S2.4.2) and the kinetics should now be proportional to photon flux. A PSS must be established for the dual wavelength idea to be beneficial.

Overall, on varying the laser power in this system we expect the rate constant for the production of C to be asymptotic to the rate constant for the first step (linear in power) as low powers and asymptotic to the product of the thermal rate constant and the fraction of B in the PSS at high power (independent of power). The *ee* should change from that expected for a simple asymmetric photosynthesis to that expected in a PSS as the power is increased.

S3 Full Reaction Scheme

The full reaction scheme for the photochemistry, including the formation of the possible non-helical isomers **5** (from cyclization of (P)-**1a** with opposite torquoselectivity) and **6** (from cyclization of conformer **1b**) is shown in Figure S2. The computed relative free energies of all conformers of **1** are shown in tabular form (Table S1). Energetically, only two conformations — **1a** and **1b twisted** — are significant at room temperature. As discussed in the main text, products **5** and **6** undergo rapid thermal ring opening back to cis-alkene **1**,[9] so do not need to be considered in the kinetic modelling. Conversely, photoproducts of **1a** are thermally stable under the reaction conditions.[9] Representative TDDFT simulated CD spectra for conformers **1a** and **1b twisted** are shown in Figure S3. It should be noted however, that due to the low energy barrier for interconversion between the conformers (S6.4), at ambient temperatures all conformers of molecule **1** would interconvert, giving an averaged CD response of zero; on average **1** is achiral.

Figure S2: Full scheme for the reaction under consideration, including the formation of undesired non-helical products (**5** and **6**) that undergo rapid ring opening. Oxidation to helicene **4** is not expected to be significant in this work because no oxidant is included in the reaction mixture.

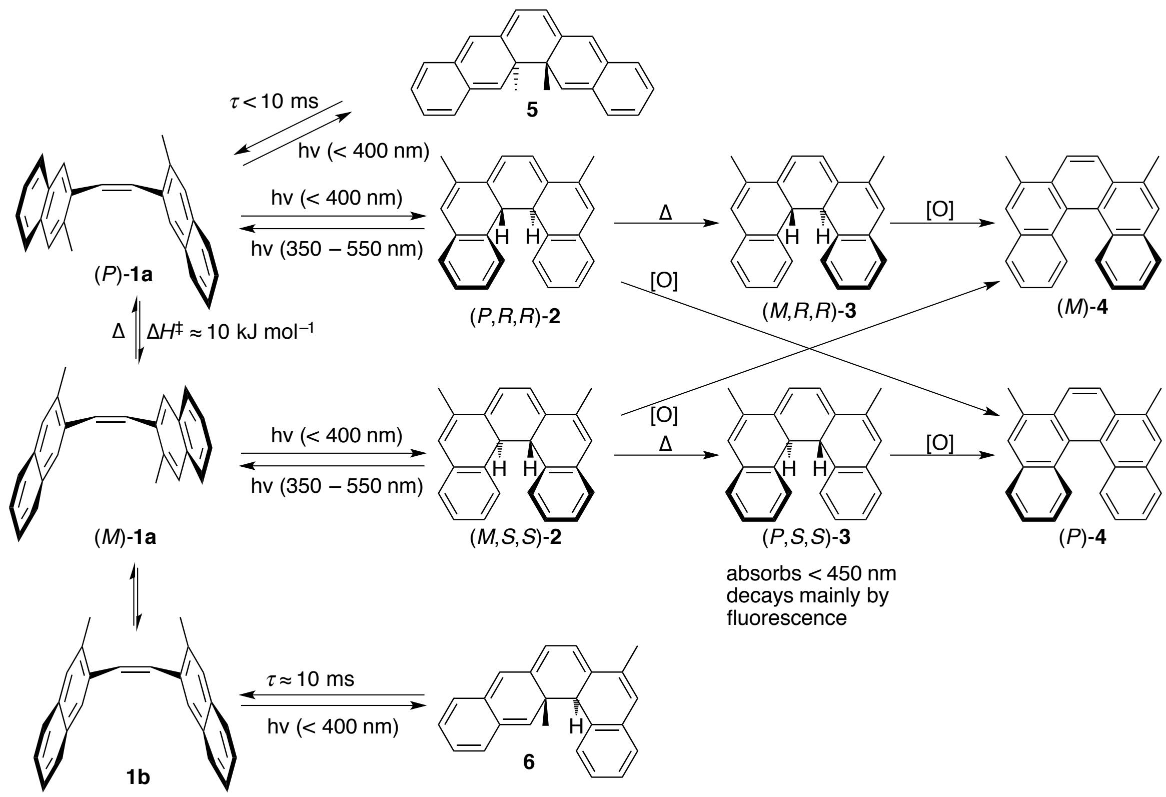

Table S1: Computed conformations of cis-dinaphthylethene 1.[a]

System	$\Delta\Delta G_{298}$[b]	$\Delta\Delta G_{298}$[c]	DOI[d]
Conformation 1 (chiral, C_2, **1a**)			
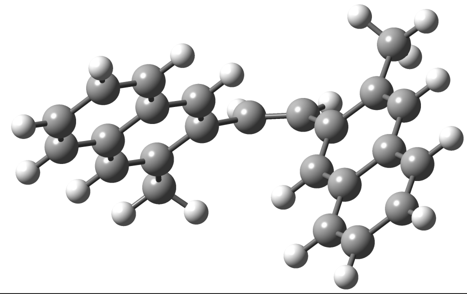	0.0	0.0 (0.0)[e]	190041, 190785
Conformation 2 (chiral, C_2)			
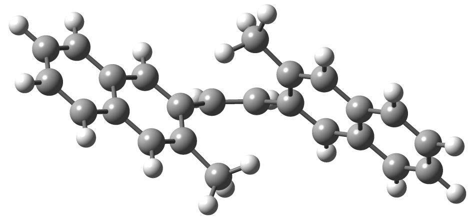	4.45	2.78	190782, 190783
Conformation 3 (meso, **1b**)			
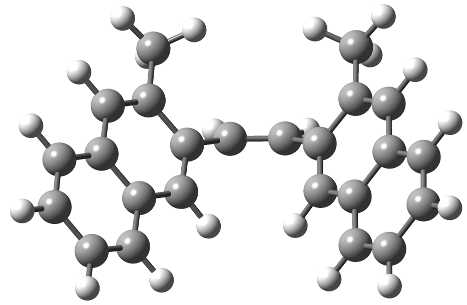	3.94	3.66	190784, 190786
Conformation 4 (chiral, **1b twisted**)			
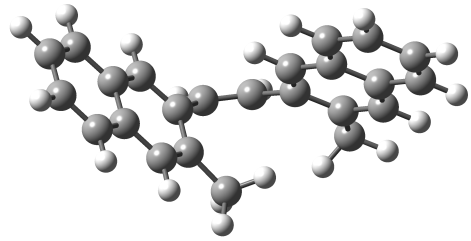	1.02	0.06 (0.0)[e]	190788, 190792

[a]Interactive models of these structures can viewed (as a FAIR data table) at `http://doi.org/10.6084/m9.figshare.1317341`
[b]Relative free energy at 298 K computed as B3LYP/6-31G(d,p)SCRF=methylcyclohexane for a standard state of 0.044 M.
[c]Relative free energy at 298 K computed as B3LYP/6-31G(d,p)SCRF=methylcyclohexane with inclusion of a D3 dispersion correction for a standard state of 0.044 M.
[d]A digital repository identifier (DOI), resolved as e.g. `http://doi.org/10.14469/ch/190041`
[e]Relative free energy at 298 K computed as B3LYP/6-311G(2df,2p)SCRF=methylcyclohexane with inclusion of a D3 dispersion correction.

Figure S3: TDDFT(100-states, CAM-B3LYP/6-311G(2df,2p)SCRF=methylcyclohexane) predicted CD spectra of conformers **1a** (blue line) and **1b twisted** (red line).

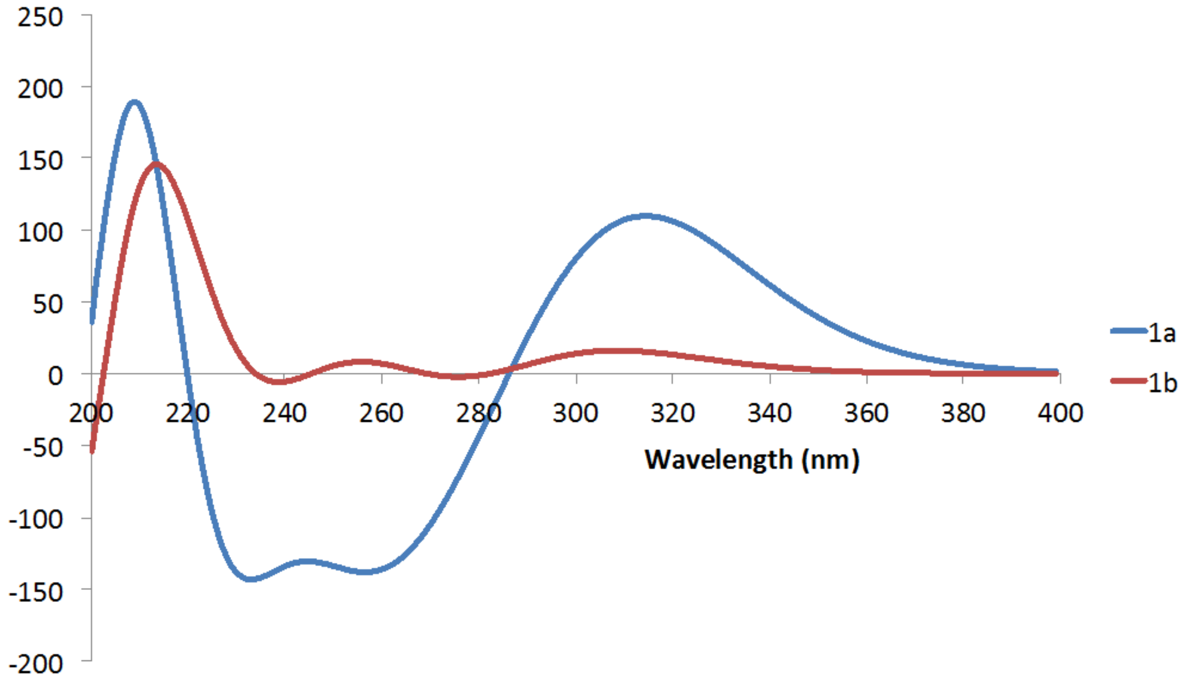

S4 Predictions for the Asymmetric Photosynthesis of 3

The model in Section S2 has been derived for a general case that is suitable for the enantioselective photosynthesis presented in this paper as outlined in Section S2.1 and that is repeated here. Predictions for the enantioselecitivity under certain conditions in the (dual wavelength) asymmetric photosynthesis of dihydrohelicene **3** are presented in this section.

$$
\begin{array}{ccccc}
(P)\text{–}\mathbf{1a} & \underset{355\,+\,532}{\overset{355}{\rightleftarrows}} & (P,R,R)\text{–}\mathbf{2} & \xrightarrow{k_{inv}} & (M,R,R)\text{–}\mathbf{3} \\
k_{rac}\Big\Updownarrow & & & & \\
(M)\text{–}\mathbf{1a} & \underset{355\,+\,532}{\overset{355}{\rightleftarrows}} & (M,S,S)\text{–}\mathbf{2} & \xrightarrow{k_{inv}} & (P,S,S)\text{–}\mathbf{3}
\end{array}
$$

S4.1 Notes on Wavelengths, g-Factors and Kinetics

From the UV/Vis spectra (Figure 4, main text) and the TDDFT-calculated CD spectra, the following points relevant to the *ee* predictions in the photosynthesis of **3** could be extracted.

1. By choosing light at $\lambda_1 = 355$ nm and $\lambda_2 = 532$ nm, starting material **1** does not absorb light at λ_2, simplifying the dual wavelength PSS analysis (Section S2.4.3).
2. 355 nm excites a single transition in the starting material **1** and 532 nm excites a single transition in intermediate **2** so $g_{\mathbf{1}}^{355}$ and $g_{\mathbf{2}}^{532}$ should be predictable.
3. The DFT calculations predict $g_{(P)-\mathbf{1a}}^{355} = +0.012$ and $g_{(P,R,R)-\mathbf{2}}^{532} = +0.0077$. The signs indicate that the predicted major product from irradiation of **1** with LCP 355 nm ($(P,R,R)-\mathbf{2}$ from excitation of $(P)-\mathbf{1a}$) preferentially absorbs LCP 532 nm and hence that opposite handedness of CP light at 355 nm and 532 nm would be required for a beneficial effect on the enantioselectivity.
4. 355 nm light excites a combination of two transitions with g-factors of opposite sign in intermediate **2**, so $g_{(P,R,R)-\mathbf{2}}^{355}$ will be a weighted average of that for the individual transitions, and will be hard to predict, but is likely to be considerably smaller in magnitude than the g-factors for the other steps.
5. The racemization barrier between $(P)-\mathbf{1a}$ and $(M)-\mathbf{1a}$ is calculated (DFT, see Section S6.2) to be ≈ 10 kJ mol^{-1} indicating fast ground state racemization at all temperatures used in this study. The condition that $r_{\mathbf{1}\to\mathbf{2}} \ll k_{rac}[\mathbf{1}]$ is likely to be met at any laser power used in this study.
6. TDDFT calculations, and studies by others,[9] suggest that the progress from the Franck-Condon regions on the S_1 surface to the ground state is barrierless, predicting that excited state racemization should not compete with product formation.
7. Final product **3** is reported by Muszkat to be photochemically stable.[9]

S4.2 Testable Predictions for the Asymmetric Photosynthesis

Based on the model presented in Section S2 and the relevant points outlined in Section S4.1, the following predictions can be made and tested regarding the enantioselective photosynthesis of **3**.

S4.2.1 CP 355 nm PSS

With high power 355 nm irradiation so that the 355 nm PSS is reached much faster than the thermal inversion, the single wavelength 355 nm PSS is fully established such that equation (42), Section S2.3.2 applies, so after thermal conversion of **2** present in the PSS to **3**

$$ee_{(M,R,R)-\mathbf{3}} = \frac{g^{355}_{(P)-\mathbf{1a}} - g^{355}_{(P,R,R)-\mathbf{2}}}{2} \tag{72}$$

The sign and magnitude of $g^{355}_{(P,R,R)-\mathbf{2}}$ is hard to predict (note 4), but if $g^{355}_{(P,R,R)-\mathbf{2}} \approx 0$, the final *ee* should around 0.006 (based on $g^{355}_{(P)-\mathbf{1a}} = +0.012$ (note 3)) in favour of $(M,R,R)-\mathbf{3}$ if LCP 355 nm is used.

S4.2.2 CP 355 nm with Thermal Inversion

With lower power 355 nm irradiation so that the thermal inversion occurs during the irradiation, the *ee* of the final product is expected to change from

$$ee_{(M,R,R)-\mathbf{3}} = \frac{g^{355}_{(P)-\mathbf{1a}}}{2} \tag{73}$$

(equation (70) when $k^{355}_{\mathbf{2}\to\mathbf{1}} \ll k^{\Delta}_{\mathbf{2}\to\mathbf{3}}$) to

$$ee_{(M,R,R)-\mathbf{3}} = \frac{g^{355}_{(P)-\mathbf{1a}} - g^{355}_{(P,R,R)-\mathbf{2}}}{2} \tag{74}$$

(equation (63), Section S2.5.1 when $k^{355}_{\mathbf{2}\to\mathbf{1}} \gg k^{\Delta}_{\mathbf{2}\to\mathbf{3}}$) as the laser power is increased. The sign and magnitude of $g^{355}_{(P,R,R)-\mathbf{2}}$ is hard to predict (note 4), but if $g^{355}_{(P,R,R)-\mathbf{2}} \approx 0$, the final *ee* should be independent of laser power at around 0.006 (based on $g^{355}_{(P)-\mathbf{1a}} = +0.012$ (note 3))

S4.2.3 Dual Wavelength PSS

If the 355 nm laser power is chosen such that $k^{355}_{\mathbf{2}\to\mathbf{1}} \gg k^{\Delta}_{3}$ (with or without thermal inversion), the *ee* should be determined by the dual wavelength PSS (Section S2.4.3). As the power at 532 nm is increased, the predicted *ee* of intermediate **2** in the PSS, and hence the predicted *ee* of the final product **3**, should change from

$$ee_{(M,R,R)-\mathbf{3}} = \frac{g^{355}_{(P)-\mathbf{1a}} - g^{355}_{(P,R,R)-\mathbf{2}}}{2} \tag{75}$$

from equation (61) when $k^{532}_{\mathbf{2}\to\mathbf{1}} \ll k^{355}_{\mathbf{2}\to\mathbf{1}}$ to

$$ee_{(M,R,R)-\mathbf{3}} = \frac{g^{355}_{(P)-\mathbf{1a}} \mp g^{532}_{(P,R,R)-\mathbf{2}}}{2} \tag{76}$$

from equation (61) when $k^{532}_{\mathbf{2}\to\mathbf{1}} \gg k^{355}_{\mathbf{2}\to\mathbf{1}}$ as the laser power is increased. Looking at individual cases, we predict

1. Using LCP 355 nm with plane polarized 532 nm when $k^{532}_{\mathbf{2}\to\mathbf{1}} \gg k^{355}_{\mathbf{2}\to\mathbf{1}}$ sets $g^{532}_{(P,R,R)-\mathbf{2}} = 0$ in equation (76) giving a final *ee* representative of $g^{355}_{(P)-\mathbf{1a}}$ only, predicted to be 0.006 (from $g^{355}_{(P)-\mathbf{1a}} =$

+0.012 (note 3)) in favour of $(M, R, R)-\mathbf{3}$ if LCP 355 nm is used. The change in $ee_{\mathbf{3}}$ as the plane polarized 532 nm power is increased should give an estimate of $g^{355}_{(P,R,R)-\mathbf{2}}$ that was hard to predict previously (note 4).

2. Using plane polarized 355 nm with LCP 532 nm when $k^{532}_{\mathbf{2}\to\mathbf{1}} \gg k^{355}_{\mathbf{2}\to\mathbf{1}}$ sets $g^{355}_{(P)-\mathbf{1a}} = 0$, predicting a final *ee* representative of $-g^{532}_{(P,R,R)-\mathbf{2}}$ only, predicted to be -0.0038 (from $g^{532}_{(P)-\mathbf{1a}} = +0.0077$ (note 3)) in favour of $(M, R, R)-\mathbf{3}$.

3. Using CP 355 nm and CP 532 nm and $k^{532}_{\mathbf{2}\to\mathbf{1}} \gg k^{355}_{\mathbf{2}\to\mathbf{1}}$ predicts an additive effect of the enantioselectivities when the wavelengths are polarized in the **opposite** sense to each other. The final *ee* is predicted to be 0.0095 (from $g^{355}_{(P)-\mathbf{1a}} = +0.012$ and $g^{532}_{(P,R,R)-\mathbf{2}} = +0.0077$ (note 3)) in favour of $(M, R, R)-\mathbf{3}$ using LCP 355 nm and RCP 532 nm.

S5 Experimental Methods

S5.1 Experimental Setup

Figure S4: Experimental setup for irradiation experiments.

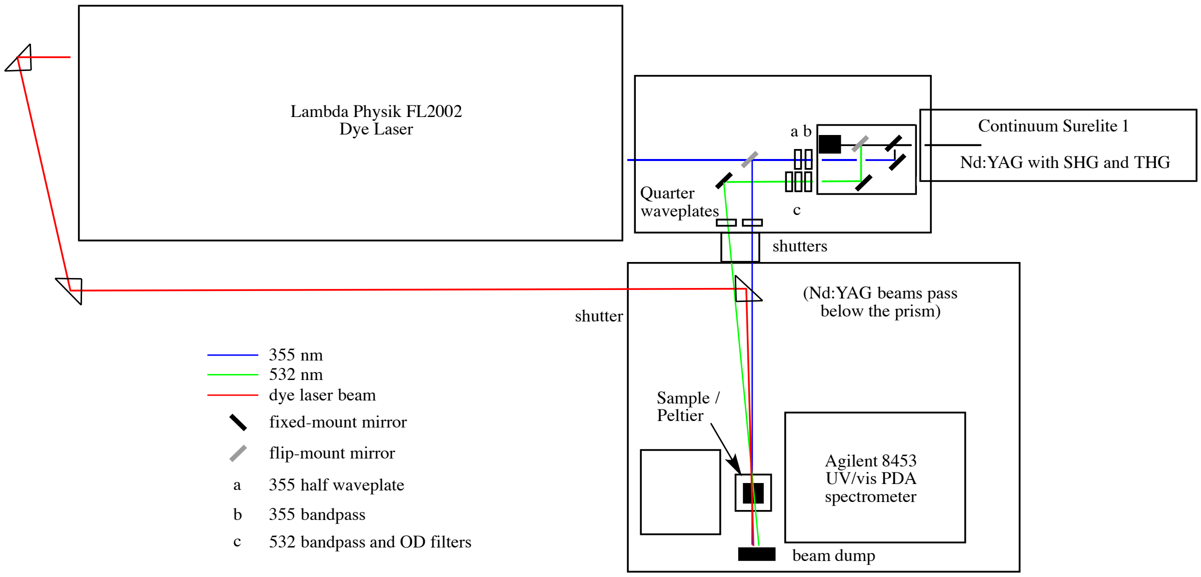

S5.2 Light Generation

The setup used for all laser (CP) radiation experiments with *in situ* UV/vis spectroscopic monitoring is shown in Figure S4. 355 nm and 532 nm light were obtained from the third and second (respectively) harmonics of a Surelite Continuum I Nd:YAG laser ($\approx$ 5 ns pulse duration, $\approx$ 6 mm beam diameter, 10 Hz repetition rate) and separated from each other (and the fundamental) with dichroic mirrors and bandpass filters. For CP light experiments, after steering the beam towards the sample with further dichroic mirrors, the plane polarization of the laser output ($\approx$ 95% horizontal for 355 nm, $\approx$ 95% vertical for 532 nm) was improved to essentially 100% with plane polarizers then circularly polarized with multi-order quarter waveplates. From laser power measurements through a rotating plane polarizer, the degree of circular polarization is estimated to be approximately 90% at either wavelength. For dual wavelength experiments, the laser beams were aligned so that they intersected at the centre of the sample, although it was found that this did not make significant different to the *ee* of the product. The laser powers were controlled by varying the Q-switch delay, tuning the third harmonic generator crystal and placing neutral density filters in the 532 nm beam.

Radiation at 440 nm ($\approx$ 7 ns pulse duration, $\approx$ 4 mm beam diameter at the sample, 10 Hz repetition rate) was generated with a Lambda-Physik FL2002 tuneable dye laser using a solution of coumarin 120 in methanol (0.25 g dm^{-3} oscillator / pre-amp solution, 0.088 g dm^{-3} amplifier solution) pumped with the 355 nm output of the Nd:YAG laser and steered to the sample using Suprasil prisms. For CP light experiments, the plane polarization ($\approx$ 95% vertical) was improved with a plane polarizer before the final prism and the light was circularly polarized with a Fresnel rhomb placed between the final prism and the sample.

Laser average power measurements were made with a Coherent PM10V1 thermopile head coupled to a Coherent FieldMate power meter. All power readings were performed directly in front of the sample

or insulating jacket. The laser powers quoted throughout this work are corrected for Fresnel reflections from any windows between the location of the power meter and the sample.

S5.3 Sample Preparation

The sample (4.0 ± 0.1 mL, 36.3 ± 0.5 μmol dm^{-3} in methylcyclohexane unless otherwise stated) was prepared in a 1 cm $\times$ 1 cm Suprasil four-window cuvette, held in a Quantum Northwest TLC50 cuvette holder, stirred vigorously throughout the irradiation and maintained at the specified temperature by a Peltier-type temperature controller. For experiments at sub-ambient temperatures the cuvette walls were purged with a gentle stream of dry air to prevent condensation. At temperatures at or below 270 K the cuvette holder was insulated in two purpose built corrugated plastic jackets fitted with quartz windows where necessary for the pump and probe light. The cuvette windows and the space between these two jackets were purged with dry air.

S5.4 UV/Vis Spectroscopy

UV/vis spectroscopy was performed using an Agilent 8453 single beam photodiode array spectrometer using the same sample holder as for the radiation experiments. For *in situ* monitoring of the photochemical reactions, the probe light was filtered using a borosilicate microscope slide to remove wavelengths below 280 nm. For monitoring fast reactions, the integration time of the spectrometer was set to 0.1 s to allow spectra to be recorded once every 4 s. For other UV spectra, then integration times was increased to 1 s. The temperature control and stirring was achieved as stated above. Unless otherwise stated all spectra were recorded after measuring a baseline of the same solvent in the same cuvette at the same temperature. For all spectra used in quantification, the average absorbance above 600 nm was subtracted from all points in the spectrum as a further baseline correction. All spectra used to quantify concentrations were recorded at 293 K.

S5.5 CD Spectroscopy

CD spectroscopy was performed at 293 K using a Jasco J715 spectropolarimeter with continual gentle stirring and temperature control from a Peltier-type controller. Owing to the small signals, the spectra were averaged over 120 scans between 240 nm and 600 nm scanning at 500 nm min^{-1} with a 2 nm data pitch and 2 nm bandwidth. The samples were contained in the same 1 cm $\times$ 1 cm Suprasil cuvette for all experiments inserted into the instrument in the same orientation.

After acquisition, the spectra were baseline-corrected by subtraction of a spectrum of the racemate of the same compound (prepared using PP light) recorded at the same temperature in the same cuvette. The spectra were further baselined using the "Peak Analyzer" function in Origin 8.6 to subtract a baseline through points at 278 nm, 332 nm, 470 nm and 570 nm to correct for small thermal fluctuations in the CD spectra. UV spectra were recorded on the same samples at the same temperature and used to correct the CD spectra for concentration by dividing the CD response at all wavelengths ΔA_λ by the absorbance of the sample at 418 nm (where only dihydrohelicene **3** absorbs significantly). All CD spectra are presented in this form ($\Delta A_\lambda / A_{418}$ *vs.* λ).

$\Delta A_\lambda / A_\lambda$ (for *ee* determination and for the comparison between the theoretical and experimental results) were obtained from $\Delta A_\lambda / A_{418}$ using the extinction coefficients obtained for the extracted spectrum of product **3** (Figure S5) in order to account only for absorbance due to product **3**. This assumes that other species absorbing at that wavelength do not show a CD response. Given that the remainder of the sample is mostly from the achiral alkene **1**, this assumption is reasonable.

S5.6 NMR Spectroscopy

NMR spectra were recorded on Bruker Advance 400 FT-NMR spectrometers operating at 400 MHz or 100 MHz for ^{1}H NMR or ^{13}C NMR respectively against an internal deuterium lock. Chemical shifts are reported in parts per million downfield of tetramethylsilane and are referenced to the (residual) solvent peak. Coupling constants are reported in hertz (Hz). Quantitative NMR spectra were recorded with $D_1 = 10$ s. Images of the NMR spectra used in this study can be found in Section S8.3.

S5.7 Procedures for Irradiation Experiments

S5.7.1 Photoswitching Between 1 and 2

The sample was cooled to 260 K and constant UV/vis spectroscopic monitoring was started. The sample was irradiated at 355 nm until the PSS was reached (no further change in UV/vis spectrum) then with 532 nm until the initial UV/vis spectrum was recovered. This cycle was repeated as necessary. At 100 mW 355 nm irradiation the PSS was reached in under 10 s. UV spectra of the PSSs can be found in the main text, Figure 2.

S5.7.2 Kinetic Experiments on the Thermal Inversion

The sample was irradiated at high power (>100 mW) at 355 nm for 10 s then the progress of the helix inversion was monitored by UV/vis spectroscopy until the absorbance at 477 nm (due to intermediate **2**) was reduced to below 5% of that recorded immediately after the irradiation. Below 280 K, the spectrometer was baselined on the starting *cis*-alkene **1** rather than the solvent and the spectra were recorded as difference spectra. A typical UV/vis time-course (280 K) is shown in the main text, Figure 3. The time courses and kinetics at other temperatures can be found in Section S8.1. After determining the kinetics at several temperature, Eyring analysis (Figure S11) was conducted to determine the activation parameters of the inversion.

S5.7.3 Competing Thermal Inversion and 532 nm Ring Opening

The sample was irradiated at high power (>100 mW) at 355 nm for 10 seconds then monitored by UV/vis spectroscopy during irradiation at 532 nm until the absorbance at 477 nm (due to intermediate **2**) was reduced to below 5% of that recorded immediately after the 355 nm irradiation. Below 280 K, the spectrometer was baselined on the starting *cis*-alkene **1** rather than the solvent and the spectra were recoded as difference spectra.

S5.7.4 PSS Experiments using High Power, Short Duration Irradiation

The sample was irradiated for 10 s at high laser power (>100 mW 355 nm, >50 mW 532 nm) with the appropriate polarization. If required, UV/vis spectra were recorded before and after the irradiation. The sample was warmed to room temperature (≈293 K) and left for at least 30 minutes (to allow complete helix inversion) before analysis by CD and UV/vis spectroscopy.

S5.7.5 Simultaneous Irradiation and Thermal Inversion

A UV/vis spectrum of the sample was recorded then the sample was irradiated at low laser power (usually <30 mW) while the progress of the reaction was monitored by UV/vis spectroscopy. When

the absorbance at 418 nm (due mainly to product **3**) reached a maximum, irradiation was stopped and the sample was warmed to room temperature for at least 30 minutes (to ensure helix inversion of any intermediate **2** remaining) before analysis by CD and UV/vis spectroscopy.

S5.7.6 Asymmetric Photodestruction of Intermediate 2

The sample at 260 K was irradiated at high power (>100 mW) with plane polarized 355 nm light for 10 seconds then monitored by UV/vis spectroscopy during irradiation with low power (<5 mW) CP 532 nm until the absorbance at 470 nm (due to intermediate **2**) was reduced to half that recorded immediately after the 355 nm irradiation. The sample was warmed to room temperature for at least 30 minutes (to ensure helix inversion of **2**) before analysis by CD and UV/vis spectroscopy.

S5.7.7 Photodestruction of Product 3

A solution containing product **3** and starting material **1** (approx. 0.6 : 0.4 ratio) was prepared using the burst irradiation method in Section S5.7.4 at 280 K, then, after helix inversion, diluted with further methylcyclohexane to obtain an absorbance of 0.1 at the pump wavelength (440 nm). The solution was monitored by UV/vis spectroscopy during irradiation at 440 nm until the absorbance at 418 nm (due to product **3**) was below 5% of the original value. For the experiments in the absence of oxygen, the sample was degassed by three cycles of freeze-pump-thaw at < 0.01 mbar then kept under reduced pressure for the photodestruction experiment.

S5.8 Quantification of Dihydrohelicene 3 in Photolysate

Preparation of a pure sample of inverted dihydrohelicene **3** was not possible due to its sensitivity to light and oxidation.[9] However, a sample of **3** as a mixture with *cis*-**1** and helicene **4** could be obtained. From this, and pure samples of *cis*- and *trans*-**1** and helicene **4**, extinction coefficients (Figure S5) for dihydrohelicene **3** were obtained as outlined below. The concentration of *cis*-**1** before irradiation was determined by comparison of the UV/vis spectrum below 340 nm with this authentic spectrum. The concentration of dihydrohelicene **3** after irradiation and complete thermal inversion was determined by comparison of the post-irradiation UV/vis spectrum with this spectrum above 390 nm.

S5.8.1 Extinction Coefficient Spectra for *cis*- and *trans*-1

Extinction coefficients between 240 nm and 500 nm for *cis*- and *trans*-**1** were obtained by recording the UV/vis spectra of samples of each alkene at a range of concentrations between 1 μmol dm^{-3} and 50 μmol dm^{-3} in methylcyclohexane. Each absorbance spectrum was divided by the product of the concentration and pathlength, and the average and standard deviation of these was taken as the extinction coefficient spectrum and uncertainties (after accounting for uncertainty in the initial stock solution concentration).

S5.8.2 Extinction Coefficient Spectrum for Helicene 4

A solution of *trans*-alkene **1** (30 mg, 0.1 mmol)[11] in methylcyclohexane (10 mL) held in a 12 mm diameter borosilicate glass vial under an atmosphere of air (atmospheric O_2 being the oxidant to convert dihydrohelicene **3** to helicene **4**) was irradiated in a Luzchem 4V photoreactor fitted with 14×8 W broadband UV-A lamps[10] (spectral range: 320–400 nm, peak 365 nm, irradiance at sample = 5 mW cm^{-2}) at ambient temperature for 4 hours, concentrated under reduced pressure and chromatographed (SiO_2 : pentane) to give helicene **4** (approx. 3 mg, 0.01 mmol, 10%) that was clean by ^{1}H and ^{13}C NMR from any

Figure S5: UV/vis spectra for all species with uncertainties.

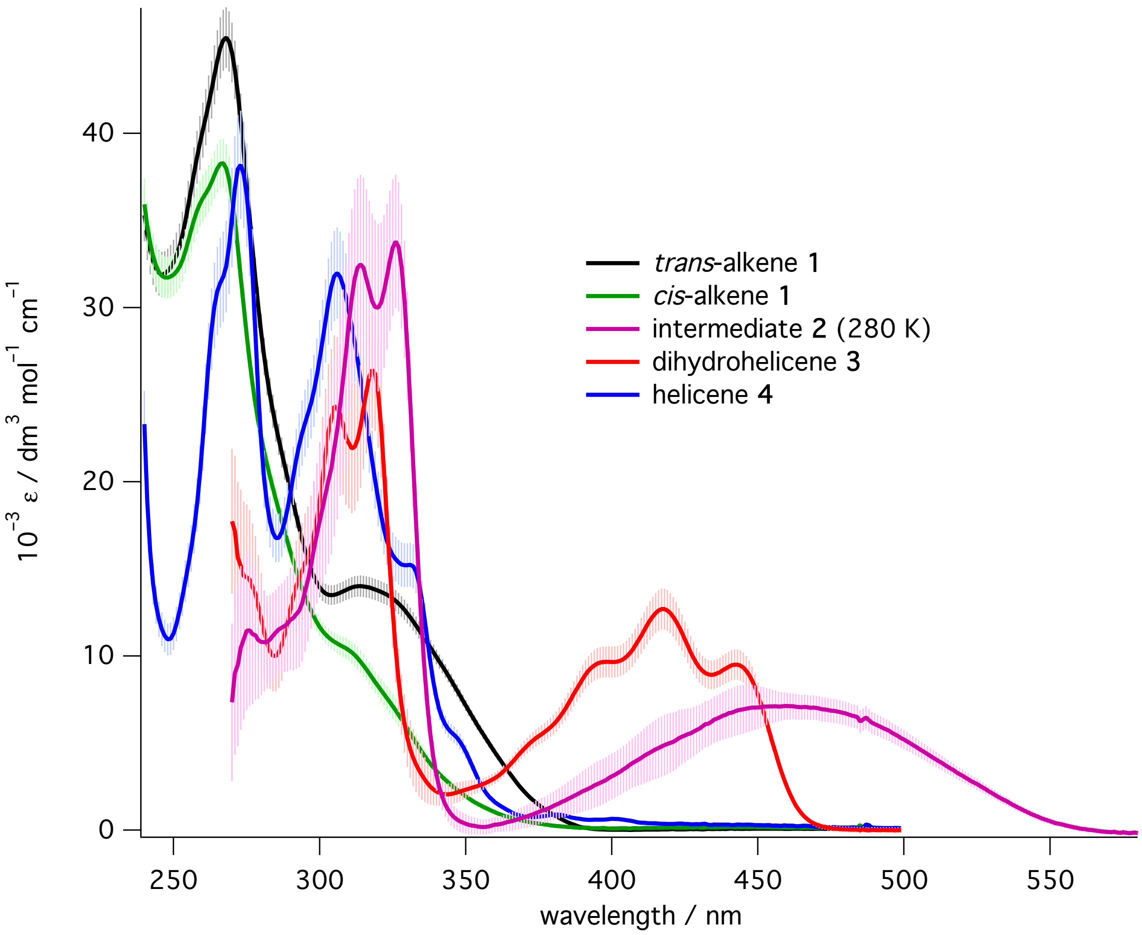

impurities that would interfere with the UV spectrum. The compound was dissolved in d_6-benzene (1.00 ± 0.02 mL) containing 1.20 ± 0.05 mmol dm^{-3} mesitylene as an internal standard. Quantitative ^{1}H NMR based on an average integral per proton across isolated signals due to both compound and standard gave an estimate of the concentration of helicene **4** as 3.8 ± 0.3 mmol dm^{-3}. An aliquot (20 ± 2 μL) of this solution was diluted into methylcyclohexane (1.98 ± 0.02 mL) and the UV/vis spectrum was of this sample was recorded ([**4**] $= 38 \pm 3$ μmol dm^{-3}) and divided by the concentration and pathlength to obtain the extinction coefficient spectrum from 270 nm to 500 nm. To obtain the extinction coefficients from 240 nm to 270 nm (the region in which C_6D_6 absorbs), a small quantity of **4** was dissolved in methylcyclohexane and the UV spectrum of this sample was scaled to match the extinction coefficient spectrum above 270 nm obtained previously.

Characterization data for helicene **4**: ^{1}H NMR (C_6D_6, 400 MHz) δ_H 8.56 (d, 2H, J = 8.5 Hz), 7.89 (s, 2H), 7.70 (dd, 2H, J = 8.0, 1.3 Hz), 7.52 (s, 2H), 7.27 (ddd, 2H, J = 8.0, 6.9, 1.1 Hz), 6.96 (ddd, 2H, J = 8.4, 6.9, 1.4 Hz), 2.57 (s, 6H). ^{1}H NMR ($CDCl_3$, 400 MHz) δ_H 8.37 (d, 2H, J = 8.5 Hz), 8.13 (s, 2H), 7.87 (dd, 2H, J = 7.0, 1.1 Hz), 7.78 (s, 2H), 7.47 (ddd, 2H, J = 8.0, 7.0, 1.1 Hz), 7.18 (ddd, 2H, J = 8.4, 6.9, 1.3 Hz), 2.89 (s, 6H). ^{13}C NMR ($CDCl_3$, 100 MHz) δ_C 132.2 (C), 131.8 (C), 131.7 (C), 130.3 (C), 129.5 (CH), 127.4 (C), 127.3 (CH), 127.0 (CH), 126.2 (CH), 123.6 (CH), 122.9 (CH), 20.2 (CH_3). HRMS (EI+): Calculated m/z for $C_{24}H_{18}$ = 306.1409, found m/z = 306.1411. LRMS (EI+): 306 ($M^{\bullet+}$), 291, 281, 276, 231, 219, 181, 169, 149, 140, 138.

S5.8.3 Extinction Coefficient Spectrum for Dihydrohelicene 3

A saturated (approx. 5 mg cm^{-3}) solution of *trans*-**1**[11] in methylcyclohexane at 300 K was stirred vigorously and irradiated in batches (5 × 4 mL) at 355 nm (0.6 W) in the same setup as used for the asymmetric photochemistry with a convex lens placed in front of the sample holder so that the laser beam was expanded to cover the full window (1 cm× 1 cm) in the cuvette holder. The irradiation was continued until the tail of the UV absorbance due to dihydrohelicene **3** reached a maximum. The solvent was removed by evaporation in the dark under a stream of nitrogen until the volume was approximately 3 mL. This mixture was chromatographed (SiO_2 : pentane) in the dark. After evaporation of the solvent under a flow of nitrogen then under reduced pressure (0.01 mbar, 20 minutes), the remaining solid was dissolved in the same internal standard solution (1 mL) as that used for the helicene quantification (1.20 ± 0.05 mmol dm^{-3} mesitylene in C_6D_6, Section S5.8.2).

Partial characterization data for dihydrohelicene **3** (further characterization was not possible owing to difficulties with purification and oxidation to helicene **4** when the compound is present at high concentrations): ^{1}H NMR (C_6D_6, 400 MHz) δ_H 7.06 (t, 2H, J = 7.5 Hz), 6.99 (d, 2H, J = 7.1 Hz), 6.93 (d, 2H, J = 7.5 Hz), 6.81 (2H, t J = 7.4 Hz), 6.40 (s, 2H), 6.05 (s, 2H), 4.25 (s, 2H), 1.95 (s, 6H).

Part of this sample was transferred to an ambered NMR tube (0.75 mL) and the remainder retained in an ambered vial. ^{1}H NMR spectroscopy indicated a mixture of *cis*-alkene **1** (0.44 ± 0.04 mmol dm^{-3}), helicene **4** (1.77 ± 0.13 mmol dm^{-3}) and dihydrohelicene **3** (1.96 ± 0.15 mmol dm^{-3}) in the NMR sample. Repeating the ^{1}H NMR spectroscopy 10 minutes after the first spectrum was acquired indicated a small (approx. 5%) further conversion of **3** to **4**. Two aliquots (40 μL) of the retained benzene solution were diluted into methylcyclohexane (1.96 ± 0.02 mL) for UV/vis spectroscopy at the start and finish of the first NMR acquisition period and the average of these two spectra was used as the UV/vis spectrum of the mixture of *cis*-**1** (8.9 ± 0.9 μmol dm^{-3}), **3** (39 ± 3 μmol dm^{-3}) and **4** (35 ± 3 μmol dm^{-3}).

The concentrations determined from the NMR analysis and the extinction coefficients for *cis*-**1** and **4** were used to estimate the contribution of those components to the UV/vis spectrum of the mixture (Figure S8.3.6). Subtraction of the contributions of these from the spectrum of the mixture gave an estimate of the UV/vis spectrum of dihydrohelicene **3** at the concentration estimated from the NMR analysis that was divided by the product of this concentration and the path length to give an estimate of the extinction coefficient spectrum of pure dihydrohelicene **3** above 270 nm.

S5.8.4 Extinction Coefficient Spectrum for Intermediate 2

A vigourously stirred solution of *cis*-**1** (36.3 ± 0.5 μmol dm^{-3}) in methylcyclohexane at 280 K (any higher temperature led to too rapid a helix inversion) was irradiated at 355 nm (0.4 W, 5 s). A UV/vis spectrum was recorded immediately after the irradiation. After complete helix inversion ($\approx$30 minutes) and warming to 293 K, a new spectrum was obtained. This second spectrum was used (in conjunction with the extinction coefficients for product **3** obtained in Section S5.8.3) to determine the concentration of **3** after inversion. Assuming quantitative helix inversion, this was taken to be the concentration of intermediate **2** formed in the irradiation. The remaining compound present in the irradiated sample was assumed to be pure *cis*-alkene **1** and the extinction coefficients determined in Section S5.8.1 were used to deconvolute the spectrum for intermediate **2** from the spectrum of the mixture just after irradiation and hence, with the estimate of the concentration of **2** present in the sample, to obtain the extinction coefficient spectrum for intermediate **2**.

S5.9 Singlet Oxygen [$O_2(a^1\Delta_g)$] Detection

Singlet oxygen [$O_2(a^1\Delta_g)$] was detected through its weak 1270 nm phosphorescence by time-resolved emission spectroscopy using a liquid nitrogen-cooled North Coast Scientific EO-817P germanium photodiode detector protected by a 1064 nm OD4 longpass filter then two OD4 1270 nm notch filters. The

excitation light (440 nm) was directed to the sample through a liquid-filled light guide at 90° to the detector. Samples of product **3** in methylcyclohexane were prepared by burst irradiation at 355 nm, diluted to obtain an absorbance of 0.1 at the pump wavelength then placed in a 1 cm × 1 cm Suprasil four-window cuvette. Degradation of the sample during this measurement (significant at 300 K) was monitored by observing the disappearance of the fluorescence spectrum using an Ocean Optics QE Pro-FL fibre-optic spectrometer protected by a 450 nm long pass filter located on the opposite side of the cuvette from the germanium photodiode and by measuring the UV/vis spectrum of the sample before and after the experiment. The sample was vigorously stirred and the temperature (275 K) was maintained using the same cell holder as for the irradiation experiments. The minimum number of decay acquisitions (between 64 and 256) in order to obtain a reasonable signal-to-noise ratio at each pulse energy were averaged. Prolonged averaging led to loss of signal following sample degradation at higher pump energies (Figure S6A).

The emission was confirmed to be due to $O_2(a^1\Delta_g)$ by observation of its disappearance upon freeze-pump-thaw degassing the sample. The authentic lifetime of $O_2(a^1\Delta_g)$ in methylcyclohexane was measured, at the same temperature using 5,10,15,20-tetraphenylporphine (TPP) as a photosensitizer (Figure S6B). The quantum yield of singlet oxygen production by dihydrohelicene **3** relative to that of TPP in the same solvent was estimated by averaging 20 data points centred on 3 μs after the laser pulse on each decay curve and plotting a graph of this signal size against pump energy. The gradients of the linear fits of these graphs for each sensitizer gave the ratio of the quantum yields of singlet oxygen from dihydrohelicene **3** and TPP as 0.8 (Figure S7).

Figure S6: Decay of the 1270 nm phosphorescence of singlet ($a^1\Delta_g$) oxygen in methylcyclohexane at 275 K generated using either (A) dihydrohelicene **3** or (B) 5,10,15,20-tetraphenylporphine (TPP) as a photosensitizer. The greater noise in graph A is a result of averaging the signal over far fewer scans due to photobleaching of the sensitizer. * indicates that the lifetime measurement at that power was not including in the average lifetime determination.

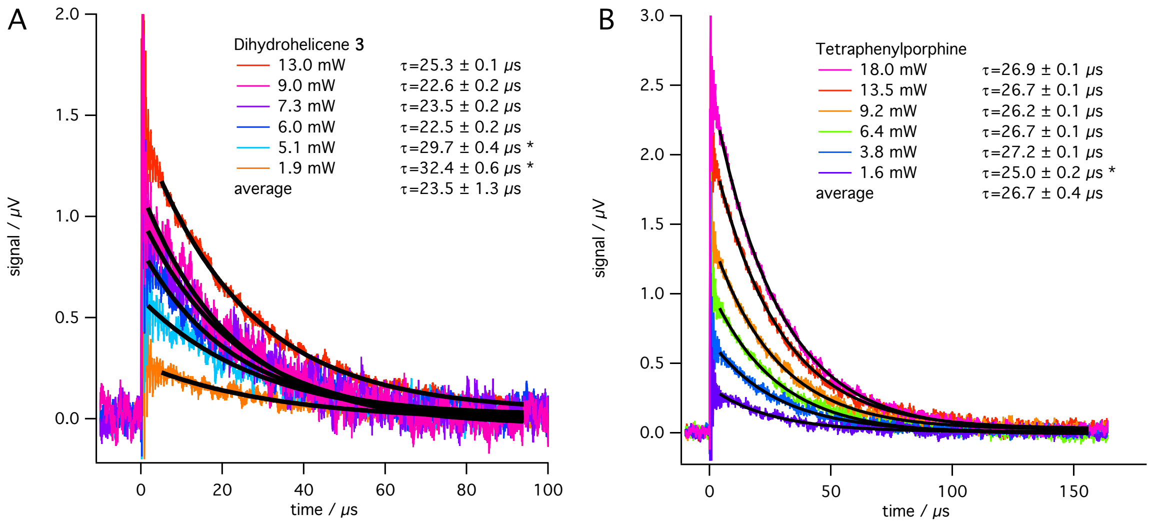

Figure S7: Determination of singlet oxygen quantum yield of dihydrohelicene **3** relative to that of tetraphenylporphine in methylcyclohexane. The gradient of the signal *vs.* power graph (at a set time delay after the laser pulse) is proportional to the quantum yield of singlet oxygen.

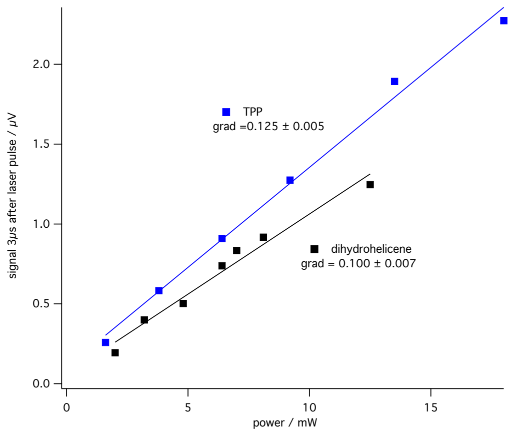

S5.10 Fluorescence Spectrum of Dihydrohelicene 3

The fluorescence spectrum recorded simultaneously to the singlet oxygen detection experiments (pumped at 440 nm) is shown below in Figure S8.

Figure S8: Fluorescence spectrum of dihydrohelicene **3** excited at 440 nm.

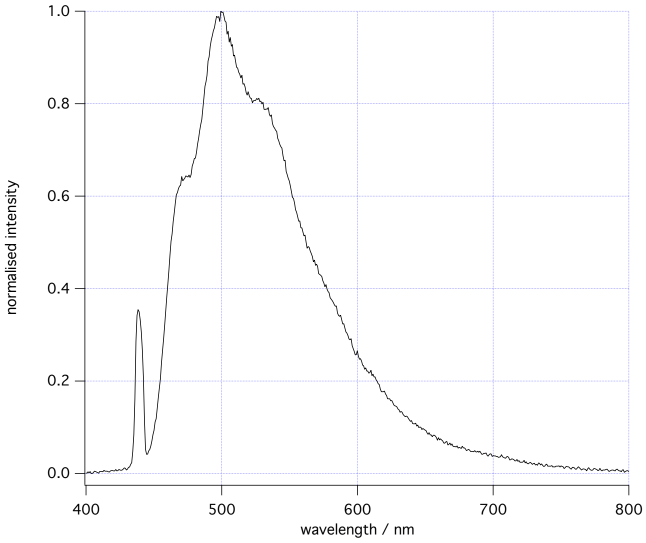

S6 Computational Methods

S6.1 General Computational Details

All calculations were performed using the Gaussian09 suite of programs.[12] Geometry optimizations and frequency calculations were performed with the relevant functional (usually the hybrid B3LYP functional[13] but others explored include Truhlar's[14] M06-2x and M06-HF double hybrid functionals and the ωB97xD range-separated hybrid functional from Head-Gordon[15] including Grimme's[16] empirical D2 dispersion model) using Pople's 6-31G(d,p) basis set and a conductor-like polarisable continuum model (CPCM)[17] for methylcyclohexane as implemented in Gaussian09. The energies were further refined using the same functional and the 6-311G(2df,2p) basis set. The results are reported as enthalpies at 298 K in kJ mol^{-1}. Location of S_0 saddle points (transition states) at S_0/S_1 avoided crossings were performed using the broken symmetry formalism when necessary.

All reported calculations of the optical and chiroptical responses of all molecules were performed using time-dependant density functional theory (TDDFT) and the Coulomb-attenuated CAM-B3LYP functional from Handy.[18] Other functionals (including M06-2x and M06-HF) did not give significant changes to the results. The calculated UV and CD spectra were generated using Gaussview 5.09[19] using the default parameters and no energy shifting was applied to the calculated excitation energies.

In this work, it is the computational accuracy in calculating the g-factors for each transition that is important, rather than the precise excitation energies and oscillator strengths (see Section S7.4 and main text "Enantiomeric Excess Assignment"). In order to assess whether the g-factors obtained by TDDFT calculations are likely to be sufficiently accurate, Mori's optimised geometry[20] of pentahelicene (as the closest comparative molecule for which accurate theoretical and experimental g-factors are known) was taken. TDDFT calculations at CAM-B3LYP/6-311G(2df,2p) were performed on pentahelicene with or without a reoptimisation of the geometry at the B3LYP/6-31G(d,p) level. For the same transition ($\approx$ 300 nm) as that reported by Mori, a g-factor of +0.0043 was obtained, regardless of reoptimisation of the geometry. This compares well to the values reported by Mori (+0.0042 (experimental) and +0.0043 (RI-CC2 computed)), suggesting that the g-factors obtained by our TDDFT methods should be sufficiently accurate for the analysis presented here without the need for higher level calculations; especially given the good agreement with experiment shown in Section S6.3 and given the experimental uncertainties in the methods used for our analysis (Section S7.4).

S6.2 Energies for 1, 2, 3 and 4 and Relevant Transition States

Figure S9 shows an enthalpy diagram for the key species in the photochemistry discussed here and pictorial views of their structures. The breakdown of the energies of these species can be found in Sections S6.5 and the atomic coordinates for all relevant species can be found in Section S8.4.

S6.3 Predicted Enantioselectivity and Helicene Length

Calculations performed by Inoue and co-workers[20] at the RI-CC2/TZVPP//DFT-D2-B97-D/TZVP level nicely reproduced the experimental CD and UV spectra of the carbo[n]helicenes for $n = 5-8$ then showed that, for the same series with $n = 5-10$ that the g-factor for the helicene increases rapidly as n increases above $n = 6$. Our TDDFT calculations at the CAM-B3LYP/6-311G(2df,2p)//B3LYP/6-31G(d,p) level using a CPCM model for methylcyclohexane shows the same trend in g-factors albeit with less accuracy in the excitation energies.

In a photosynthesis of octahelicene, Calvin and co-workers[21] obtain an enantioselectivity of 0.02, much higher than in the previous photosynthesis of hexahelicene by Kagan[22] of 0.0035, potentially in line with the increased g-factors of the products. However, Calvin assigns the induced asymmetry to the

Figure S9: Calculated enthalpy diagram (CPCM(methylcyclohexane)/B3LYP/6-311G(2df,dp)//CPCM(methylcyclohexane)/B3LYP/6-31G(d,p) of the main species involved in this photochemistry. *energy of the thermal ring opening/closing transition state is subject to greater uncertainty that the other values owing to considerable multi reference character to the wavefunction at this geometry.

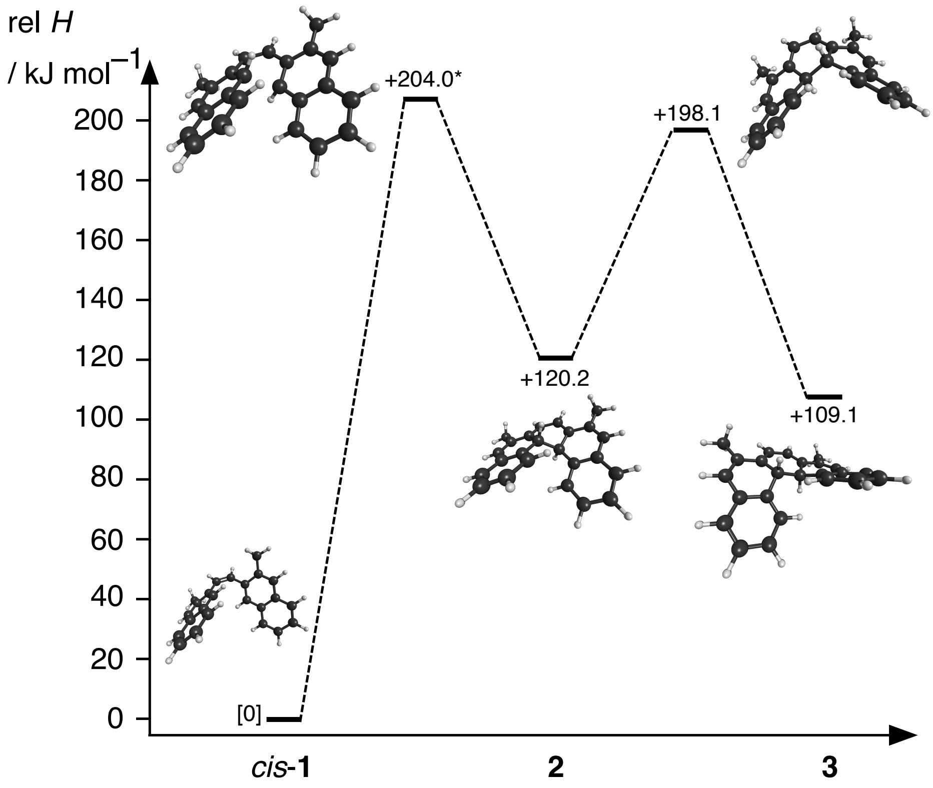

initial photochemical ring closure and not to an asymmetric photodestruction of the products or any intermediate, so only the g-factors of the enantiomeric reacting conformers of the starting material should be relevant. Given these conformers are in rapid equilibrium, this cannot be measured. Using the TDDFT methods employed throughout this work, g-factors of the presumed reacting conformers are predicted as 0.008 for 3-[(1Z)-2-(2-naphthalenyl)ethenyl]phenanthrene (Kagan's hexahelicene precursor) and 0.05 for 2-[(1Z)-2-(3-phenanthrenyl)ethenyl]benzo[c]phenanthrene (Calvin's octahelicene precursor), in line with the enantioselectivites observed in these reactions being a result only of preferential excitation of the enantiomeric conformers of the starting materials only.

Although the thermal inversion of the longer dihydrohelicenes is expected to be slow for this dual-wavelength approach to be used except at highly elevated temperatures,[23] such substrates may prove amenable to the approach outlines in this paper with an intermolecular thermal reaction (e.g., an oxidation to the helicene) replacing the helix inversion used here.

S6.4 Racemization of (P)- and (M)-1a

The lowest energy pathway located for the interconversion of the enantiomeric conformations, (P)- and (M)-**1a** occurs through a three-step mechanism *via* two enantiomers of intermediate **1b** and an achiral transition state as shown in Figure S10. The activation enthalpy for this process (10 kJ mol^{-1}) is consistent with a first order rate constant ($k \sim 10^9$ s^{-1} at 298 K) far in excess of the rate of conversion

of **1** to **2**, so depletions of either enantiomeric conformation of the starting material resulting in mass-action effects on the enantioselectivity should not be a problem with this system.

Figure S10: Racemization of (P)- and (M)-**1a**. Energies are presented as enthalpies at 298 K.

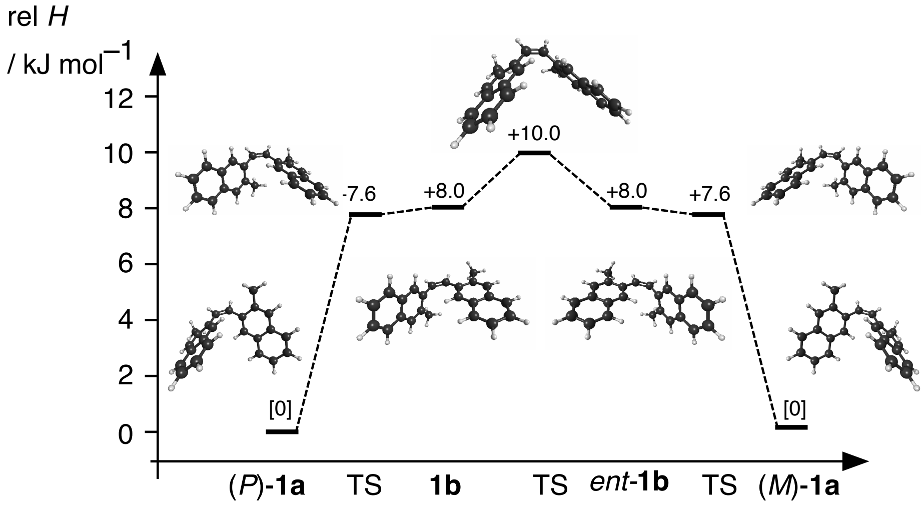

S6.5 Energy Breakdown for all Calculated Structures

The SCF energies, enthalpies and free energies from the B3LYP calculations are given in Section S2 for the important species in this study. In addition, the energy of helicene **4** and the transition state for its racemization are also given as a comparison to the helix inversion transition state (**2** to **3**).

Table S2: Energy breakdown for all species calculated using the CPCM solvent model for methyl-cyclohexane and the B3LYP hybrid functional. All energies are given in hartrees (E_h) and imaginary frequencies (where appropriate) are given in wavenumbers (cm^{-1}).

	6-31G(d,p)				6-311G(2df,2p)
Species	SCF Energy	H	G	ν imag.	SCF Energy
**1a**	-926.6571	-926.2724	-926.3423	-	-926.9035
**1a** to **2** TS	-926.5780	-926.1975	-926.2625	-978.3	-926.8217
**2**	-926.6147	-926.2294	-926.2940	-	-926.8583
**2** to **3** TS	-926.5830	-926.1990	-926.2616	-72.7	-926.8275
**3**	-926.6191	-926.2335	-926.2980	-	-926.8630
**4**	-925.4702	-925.1075	-925.1706	-	-925.7129
**4** racemization TS	-925.4301	-925.0686	-925.1301	-101.9	-925.6738
**1a** to **1b** TS	-926.6532	-926.2694	-926.3367	-23.6	-926.9005
**1b**	-926.6541	-926.2694	-926.3389	-	-926.9005
**1b** racemization TS	-926.6519	-926.2683	-926.3351	-21.1	-926.8986

S7 Further Results and Discussion

Further discussion of interesting aspects of this photochemistry that are not vital to the main message of the paper are included here.

S7.1 Kinetics of Dihydrohelicene Inversion

Eyring analysis on the kinetics of the inversion of intermediate **2** to product **3** was conducted after determining the rate constants for this process at six temperatures between 250 K and 300 K (Figure S11, see Section S8.1 for the data used to determination of the rate constants). The experimental activation parameters are $\Delta H^{\ddagger} = +71.5$ kJ mol^{-1}, $\Delta S^{\ddagger} = -38.7$ J K^{-1} mol^{-1}. DFT calculations using the hybrid B3LYP functional gave a small overestimate of the activation enthalpy (+77.8 kJ mol^{-1}) for this process. Otherwise identical calculations with the dispersion-corrected hydrid ωB97xD or the double hybrid M06-2x functionals gave much increased overestimates of the activation enthalpy (+86.1 kJ mol^{-1} and +85.7 kJ mol^{-1}) consistent with our calculations on similar bond rotation processes in aromatic systems.[24] This is presumably a result of fortuitous error cancellation but, owing to the importance of this activation barrier throughout the work, the B3LYP functional was retained for the rest of the study. Without energy the energy refinement using the 6-311G(2df,2p) basis set, the inversion barrier is further overestimated by $2-3$ kJ mol^{-1} using any functional.

Figure S11: Eyring plot for the thermal inversion of intermediate **2** to product **3**. The rate constant at each temperature was determined from the average of those measured at **2** at 447 nm and 417 nm. The kinetics fitting at each temperature can be found in Section S8.1.

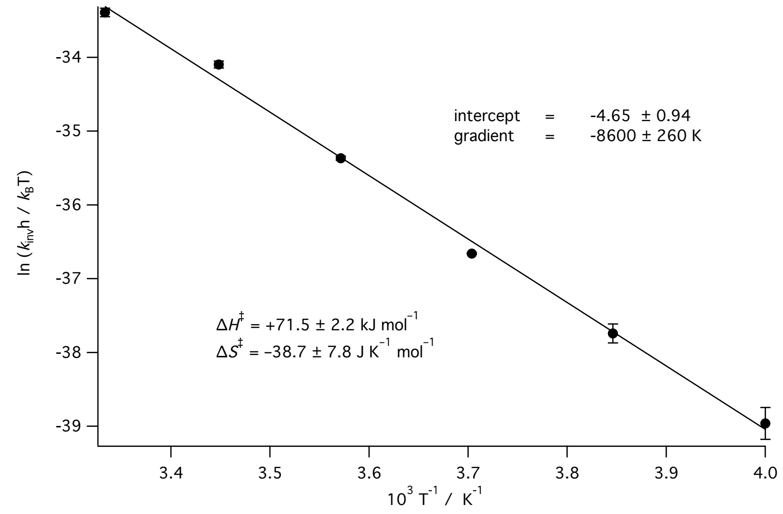

The same DFT calculations predict that the removal of the methyl groups from the dihydrohelicenes (**2**/**3**) results in a reduction of the activation enthalpy by around 5 kJ mol^{-1} regardless of the functional employed in the calculations and that this effect is also present in the activation barrier for the helicene **4**. These activation enthalpies for the dihydrohelicene inversion are significantly lower than that for the racemization of helicene **4** (+99.6 kJ mol^{-1} from DFT calculations) or [5]helicene itself (+94.0 kJ mol^{-1} from DFT calculations or +96 kJ mol^{-1} as measured by Stedmeyer in *iso*-octane solution[25]), indicating that the inversion of the dihydro[5]helicenes is significantly faster that the racemization of the corresponding [5]helicenes. It may be possibly, therefore, to perform this asymmetric photosynthesis of dihydrohelicene **3** with simultaneous oxidation to helicene **4** at a temperature such that the thermal inversion can occur readily while avoiding racemization of the helicene product.

S7.2 Kinetics and Quantum Yields of Ring Opening Reaction

When a solution of intermediate **2** is irradiated with low power 532 nm light at a temperature at which the thermal inversion also occurs, the rate of disappearance of intermediate **2** is equal to the sum of the rates of the thermal and photochemical processes:

$$\frac{\mathrm{d}[\mathbf{2}]}{\mathrm{d}t} = k^{\Delta}_{\mathbf{2}\to\mathbf{3}}[\mathbf{2}] + k^{532}_{\mathbf{2}\to\mathbf{1}}[\mathbf{2}] \tag{77}$$

Given that only intermediate **2** absorbs at 477 nm, A_{477} should decay with an observed rate constant, k_{obs}, where

$$k_{\mathrm{obs}} = k^{\Delta}_{\mathbf{2}\to\mathbf{3}} + k^{532}_{\mathbf{2}\to\mathbf{1}} \tag{78}$$

Substituting the expression for the photochemical rate constant from equation 18, we obtain

$$k_{\mathrm{obs}} = k^{\Delta}_{\mathbf{2}\to\mathbf{3}} + \frac{q_{in}\Phi^{532}_{\mathbf{1}\leftarrow\mathbf{2}}\varepsilon^{532}_{\mathbf{2}}\ell\ln 10}{V} \tag{79}$$

where $\Phi^{532}_{\mathbf{1}\leftarrow\mathbf{2}}$ is the quantum yield for the ring opening at 532 nm, $\varepsilon^{532}_{\mathbf{2}}$ is the extinction coefficient of **2** at 532 nm, ℓ is the path length of the sample and V is the volume of the sample. The photon flux incident on the sample, q_{in}, can be determined from the incident power

$$q_{in} = \frac{P\lambda}{hcN_{\mathrm{A}}} \tag{80}$$

where P is the laser power, h is the Planck constant, c is the speed of light and N_{A} is Avagdro's number. Substituting equation (80) into equation (79) gives

$$k_{\mathrm{obs}} = k^{\Delta}_{\mathbf{2}\to\mathbf{3}} + \frac{\lambda\Phi^{532}_{\mathbf{1}\leftarrow\mathbf{2}}\varepsilon^{532}_{\mathbf{2}}\ell\ln 10}{hcN_{\mathrm{A}}V}P \tag{81}$$

Assuming no significant heating of the sample due to the radiation, the thermal rate constant is independent of laser power, so a graph of k_{obs} *vs.* P (Figure S12) should be linear with gradient, m, given by

$$m = \frac{\lambda\Phi^{532}_{\mathbf{1}\leftarrow\mathbf{2}}\varepsilon^{532}_{\mathbf{2}}\ell\ln 10}{hcN_{\mathrm{A}}V} \tag{82}$$

allowing the product of the quantum yield and the extinction coefficient, $\Phi^{532}_{\mathbf{1}\leftarrow\mathbf{2}}\varepsilon^{532}_{\mathbf{2}}$, to be determined at each temperature. From Figure 4 (main text), $\varepsilon^{532}_{\mathbf{2}} = 1940 \pm 150$.

From the gradient of this graph, the quantum yield for the ring opening initiated by 532 nm light at 280 K is estimated as 0.31 ± 0.03. The change in gradient with temperature is probably due mainly to the fact that 532 nm lies well off the peak maximum for the electronic transition under investigation, hence peak broadening with increasing temperature increases the extinction coefficient in this region.

Determination of the quantum yield for the ring closing reaction is complicated by significant absorbance of intermediate **2** at all wavelengths at which *cis*-**1** absorbs. Using the PSS ratio of **2** and *cis*-**1** at 355 nm results in a problem due to the fact that 355 nm does not excite **2** to a single excited state, so the quantum yield at 532 nm cannot reliably be used here. Consequently, this value has not been determined.

S7.3 Variable Power CP 355 nm Radiation During Thermal Inversion

From equations (62), (68), (70) and (71) in Sections S2.5.1 and S2.5.2, varying the power at 355 nm should result in a change in rate determining step for the formation of dihydrohelicene **3** from *cis*-alkene **1**

Figure S12: Variation of the observed first-order rate constant for the disappearance of intermediate **2** with competing thermal inversion and photochemical ring opening at three temperatures. The crude data use to generate this graph can be found in Section S8.2.

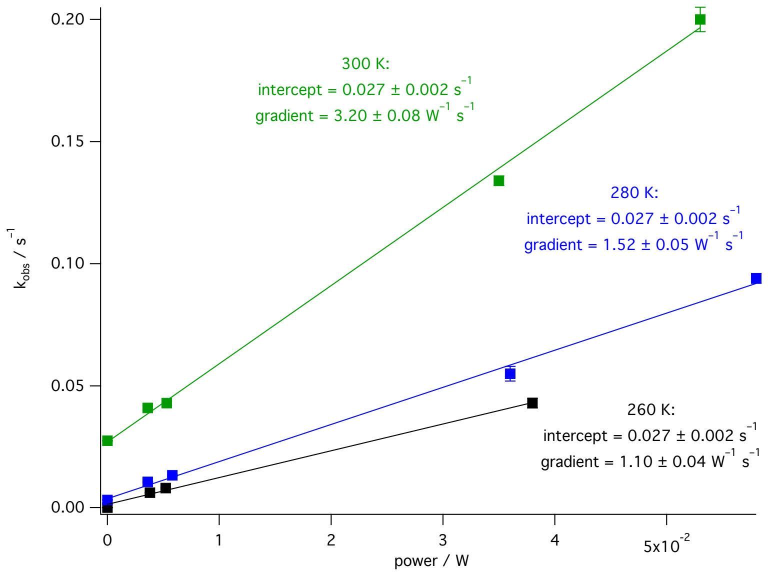

at the power at which the rate of the photochemical ring opening (**2** $\rightarrow$ **1**) equals that of the thermal helix inversion (**2** $\rightarrow$ **3**). This should be marked by a change from the rate being linear in laser power (low power) to being independent of laser power (high power). In addition, the effect of $g_{\mathbf{2}}^{355}$ on the *ee* of **3** should also change through this point (see Section S4.2). The variation of the rate of formation of **3** at 300 K (fitted to a first-order kinetic model, although this should only be truly accurate at extreme power values) with laser power (Figure S13) indicates a clear transition through this point. However, when the photolysis was performed with CP 355 nm light at powers significantly above and below this point, no difference in the *ee* in product **3** is observed, suggesting that $g_{\mathbf{2}}^{355} \approx 0$.

In addition to this, the observation from the main text that the introduction of plane polarized 532 nm light to the CP 355 nm photo stationary state has no effect on the *ee* also supports the idea that this g-factor is negligible. From Figure S5, 355 nm occurs at the overlap of two electronic transitions that are predicted (see main text Figure 1) to have fairly equal and opposite g-factors, so an overall g-factor of approximately zero is not unexpected.

S7.4 Determination of *ee* and Comparing Theory and Experiment

For a single enantiomer, A_R, the size of the CD response (compared to the UV absorbance) at any probe wavelength, λ_{probe}, is, by definition (see equation (1)), equal to the g-factor of the compound at that wavelength

$$\frac{\Delta\varepsilon_{\mathrm{A}_R}^{\lambda_{\mathrm{probe}}}}{\varepsilon_{\mathrm{A}_R}^{\lambda_{\mathrm{probe}}}} = g_{\mathrm{A}_R}^{\lambda_{\mathrm{probe}}} \tag{83}$$

Providing that the UV and CD are measured at the same concentration and in cuvettes of equal path length, the CD response in terms of absorbance, A, should be given by the same expression:

$$\frac{\Delta A_{\mathrm{A}_R}^{\lambda_{\mathrm{probe}}}}{A_{\mathrm{A}_R}^{\lambda_{\mathrm{probe}}}} = g_{\mathrm{A}_R}^{\lambda_{\mathrm{probe}}} \tag{84}$$

Figure S13: Variation of the observed first order rate constant for the formation of **3** at 300 K with laser power (top) showing the asymptotic behaviour (asymptotes as drawn are for illustration only) at high and low power and the transition through the critical power around 9 mW at which the rate determining step changes from the ring closing photochemical step to the thermal inversion. The CD spectra show the *ee* of final product **3** to be invariant of power, suggesting that the ring opening of intermediate **2** initiated by 355 nm light is unselective, hence $g_{\mathbf{2}}^{355} \approx 0$.

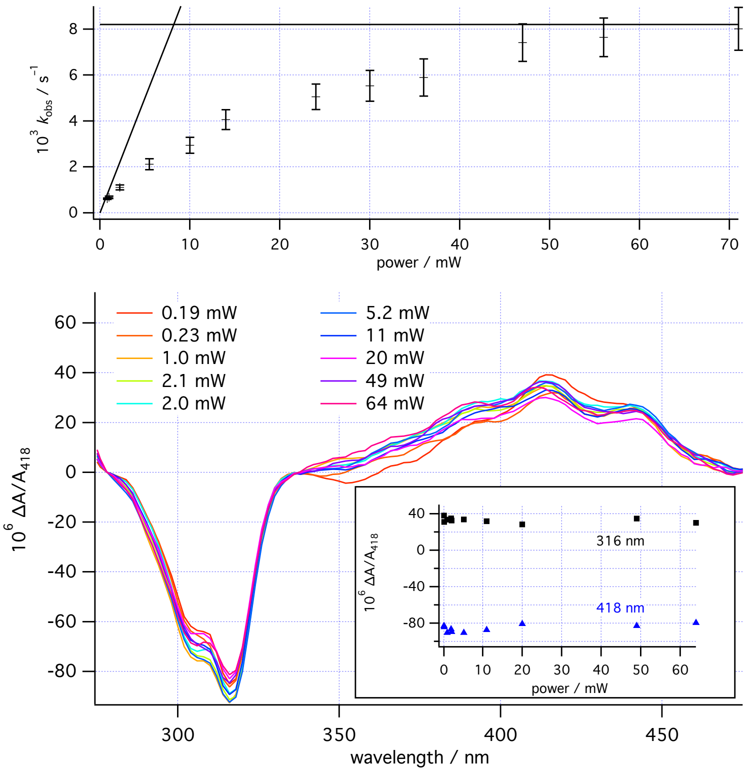

while for a scalemic mixture of A, the CD response is simply scaled by the *ee* of A_R (it is no longer necessary to specify the enantiomer in excess if the *ee* and *g*-factors are defined consistently)

$$\frac{\Delta A_{\mathrm{A}}^{\lambda_{\mathrm{probe}}}}{A_{\mathrm{A}}^{\lambda_{\mathrm{probe}}}} = g_{\mathrm{A}_R}^{\lambda_{\mathrm{probe}}} ee_{\mathrm{A}_R} \tag{85}$$

Substituting the predicted *ee* values from Section S4 into equation (85), then using the TDDFT calculated *g*-factors for the relevant compounds and wavelengths allows conversion of the predictions into values that can be directly compared to the measured CD responses in Figure 5 (main text) after correcting for the relative extinction coefficients in Figure 4 (main text) assuming that dihydrohelicene **3** is the only CD-active compound in the mixture (reasonable because the rest of the photolysate is almost entirely achiral alkene **1**). For example, for the LCP 355 nm PSS alone (invert sign for RCP radiation), the expected *ee* of product **3** (from equation (72)) is

$$ee_{(M,R,R)-\mathbf{3}} = \frac{g_{(P)-\mathbf{1}}^{355} - g_{(P,R,R)-\mathbf{2}}^{355}}{2} \tag{86}$$

hence, at a probe wavelength λ_{probe}, the theoretical CD response due to dihydrohelicene **3** should be

$$\frac{\Delta A_{\mathbf{3}}^{\lambda_{\text{probe}}}}{A_{\mathbf{3}}^{\lambda_{\text{probe}}}} = \frac{g_{(M,R,R)-\mathbf{3}}^{\lambda_{\text{probe}}}\left(g_{(P)-\mathbf{1}}^{355} - g_{(P,R,R)-\mathbf{2}}^{355}\right)}{2} \tag{87}$$

Given that: $g_{(P,R,R)-\mathbf{2}}^{355} \approx 0$ (see Section S7.3 and main text); $g_{(M,R,R)-\mathbf{3}}^{418} = -0.0073$ and $g_{(P)-\mathbf{1}}^{355} = +0.012$ (from the TDDFT, which should be suitably accurate for this work, see Section S6.1); and only species **3** absorbs at 418 nm the expected CD response at 418 nm after irradiating *cis*-alkene **1** with LCP 355 nm light is

$$\frac{\Delta A_{\mathbf{3}}^{418}}{A_{\mathbf{3}}^{418}} = \frac{-0.0073 \times (+0.012 - 0)}{2} = -4.4 \times 10^{-5} \tag{88}$$

compared to -3.5×10^{-5} observed experimentally. The reason for the discrepancy (incomplete circular polarization of the laser light) is discussed in the "Enantiomeric Excess Assignment" section of the main text along with the results of similar calculations performed on the results of several irradiation conditions and at the two CD peaks at 418 nm and 316 nm. At 316 nm other achiral species absorb light, so the absorbance at this wavelength is estimated using the calculated extinction coefficients at 418 nm and 316 nm.

As seen in the main text, Table 1, the theoretical results from these calculations are in good agreement with the observed CD responses. Although the possibility for cancellation of errors in this model cannot be discounted, this is consistent with the DFT g-factors being reliable and the reaction behaving according to the predictions made in Section S4. From this, we assign the *ee* values for each irradiation as half the relevant linear combination of g-factors scaled by 0.8 to account for incomplete circular polarization. The data used to generate the numbers is Table 1 (main text) can be found in Table S3.

Table S3: g-Factor data used to generate Table 1 (main text).

Species	λ / nm	calculated g
(P)-**1a**	355	+0.012
(P,R,R)-**2**	355	≈ 0
(P,R,R)-**2**	532	+0.0077
(M,R,R)-**3**	418	-0.0073
(M,R,R)-**3**	316	+0.0062

S7.5 Asymmetric Photodestruction of Dihydrohelicene with CP 440 nm Light

When the degassed photodestruction of racemic dihydrohelicene **3** is performed with LCP 440 nm light to a conversion of approximately 0.5, the dihydrohelicene **3** remaining in solution results in a small but significant CD spectrum showing preferential destruction of (M,R,R)-**3**, which is the enantiomer that is preferentially formed when *cis*-**1** is excited with LCP 355 nm light (see main text, Figures 1 and 5A). Given that Figure S5 suggests that the same transition in **3** is excited at 355 nm as at 440 nm, this result suggests that the photodestruction of **3** with LCP 355 nm light would occur in such a way that the same enantiomer of **3** that is preferentially formed by irradiation of *cis*-**1** with LCP 355 nm light is also preferentially destroyed, reducing the *ee* of any **3** formed under conditions that increase the degree of photodestruction of **3**.

S8 Supplementary Data

S8.1 Kinetic Data for Thermal Inversion

Figure S14: Kinetic data for the thermal inversion of intermediate **2** to product **3** at six temperatures used to generate the Eyring plot in Figure S11. The average rate constant from the two wavelengths shown was used in the Eyring analysis.

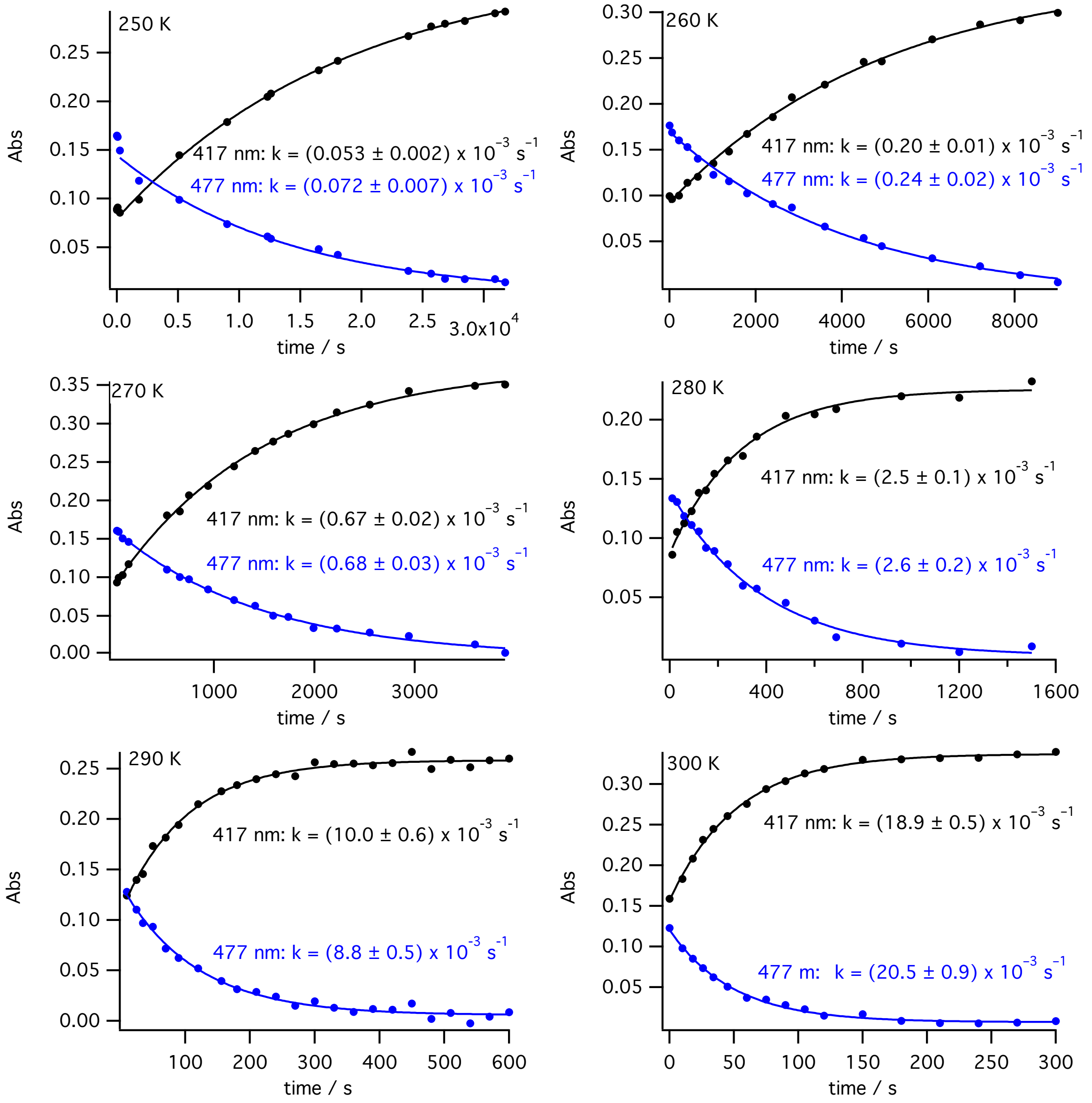

S8.2 Competing Thermal Inversion and Ring Opening

The following spectra show the kinetics of the decay of the signal at 477 nm when solutions containing intermediate **2** at three different temperatures are irradiated with 532 nm light at varying powers. The

observed first-order rate constants from these graphs were used to perform the ring-opening quantum yield investigation in Section S7.2.

Figure S15: Normalized decays of intermediate **2** followed at 477 nm with varying laser powers at 300 K.

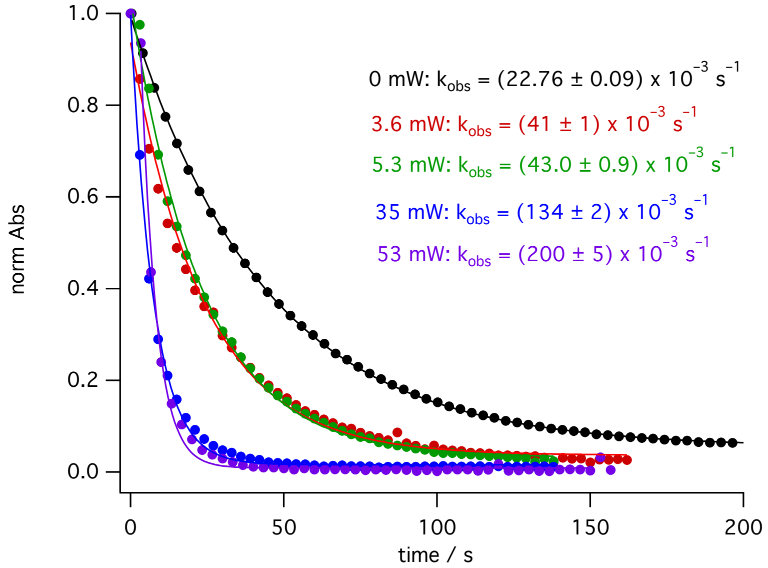

Figure S16: Normalized decays of intermediate **2** followed at 477 nm with varying laser powers at 280 K.

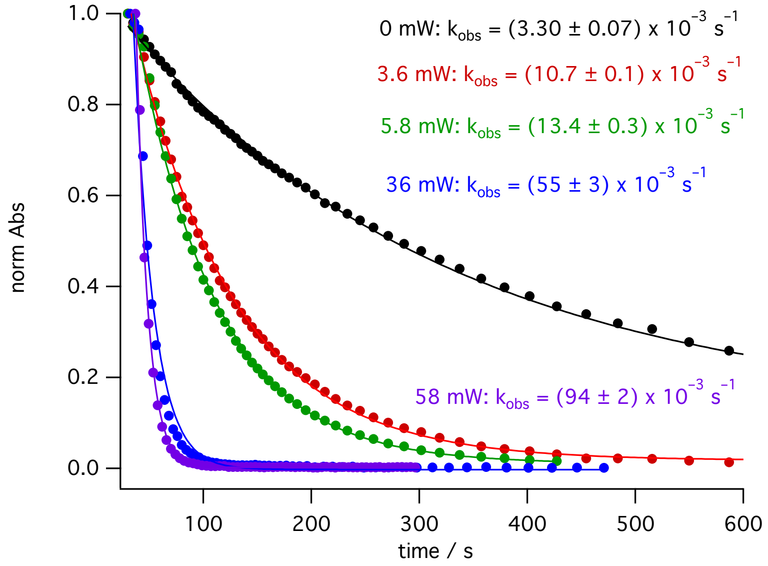

Figure S17: Normalized decays of intermediate **2** followed at 477 nm with varying laser powers at 260 K. The data for 0 mW extends well off the right hand side of the graph, but the graph has been truncated to allow easy viewing of the other traces.

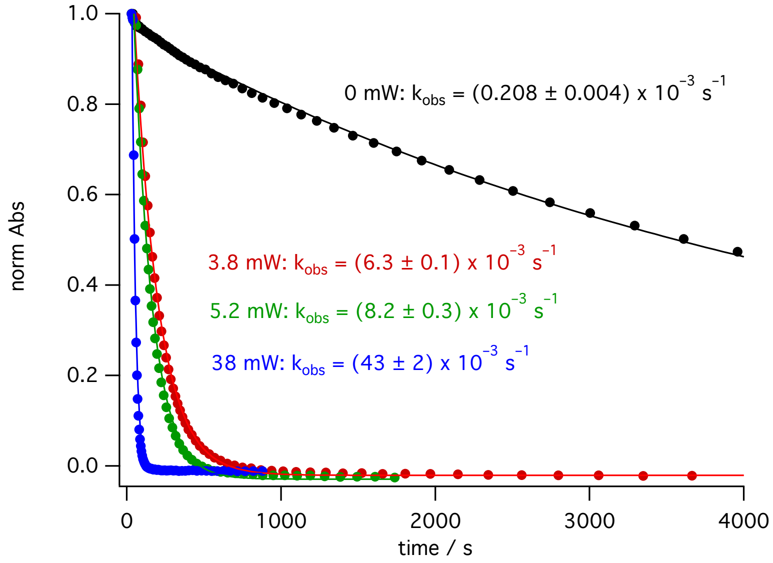

S8.3 NMR and UV Spectra used for Characterization and Quantification

S8.3.1 ^{1}H NMR spectrum of helicene 4 in $CDCl_3$

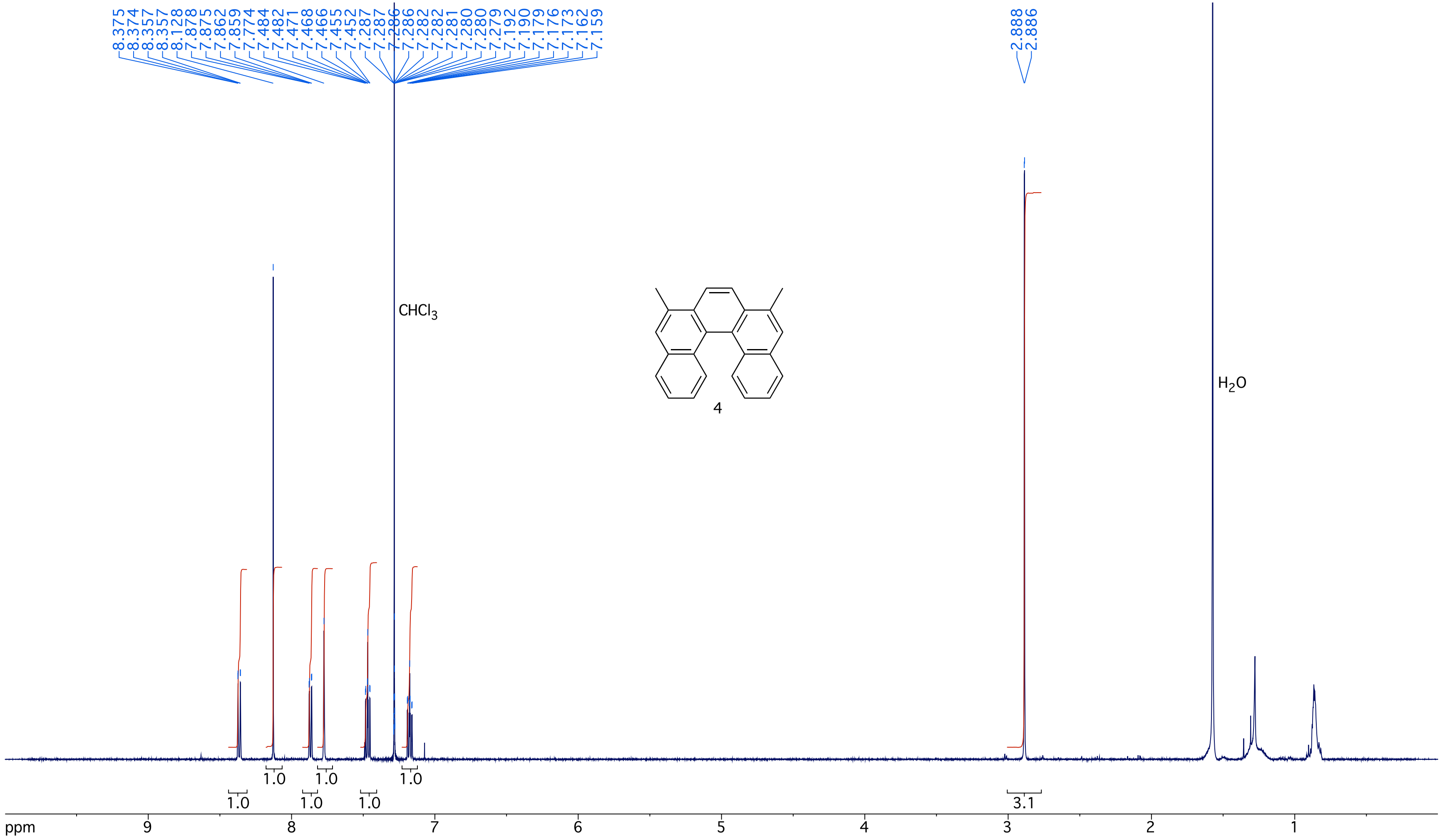

S8.3.2 ^{13}C NMR spectrum of helicene 4 in $CDCl_3$

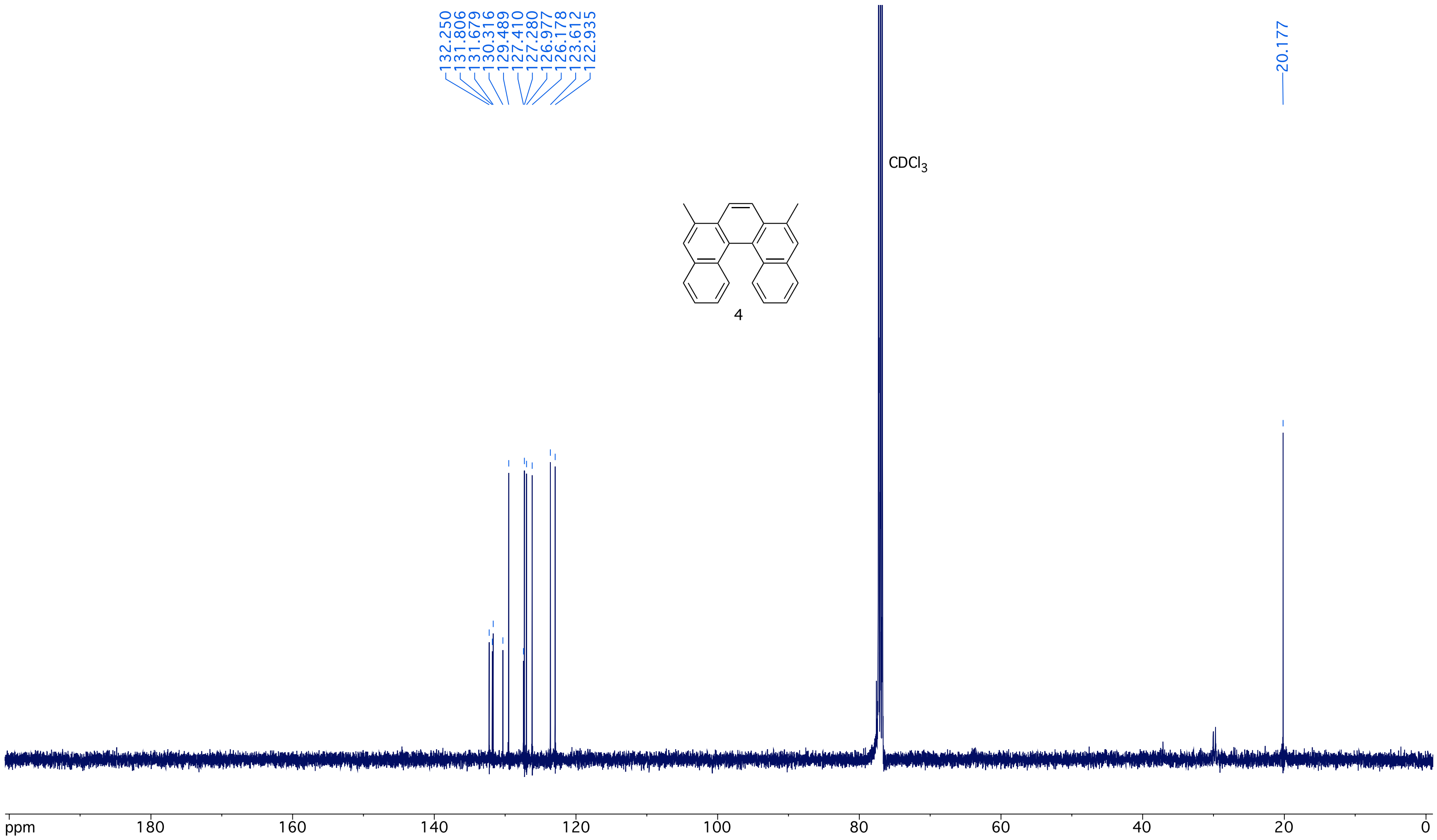

S8.3.3 DEPT-135 NMR spectrum of helicene 4 in C_6D_6

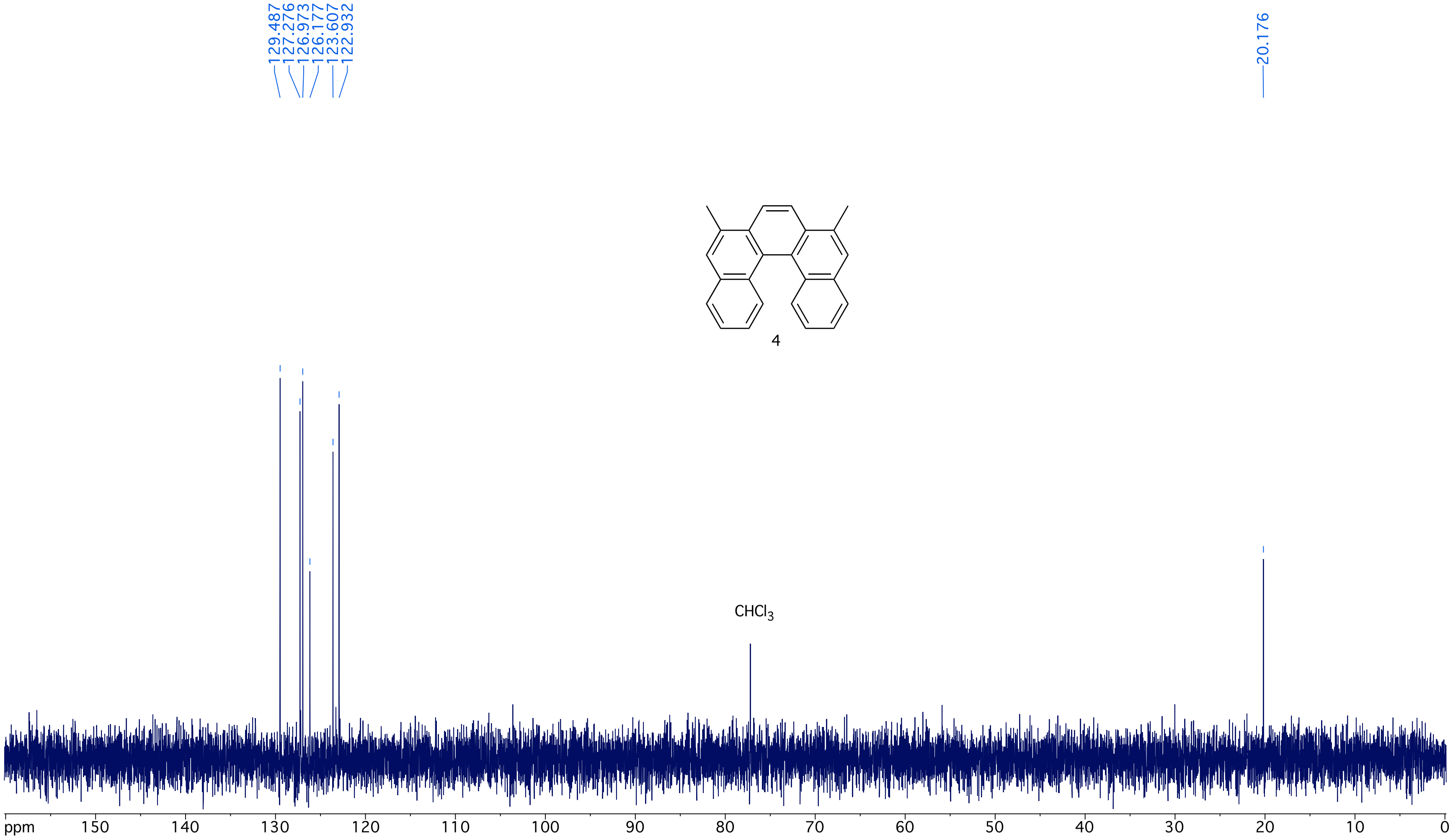

S8.3.4 ^{1}H NMR spectrum for quantification of helicene 4 *vs.* mesitylene

[mesitylene] $= 1.20 \pm 0.05$ mmol dm^{-3} $\Longrightarrow$ [**4**] $= 3.82 \pm 0.30$ mmol dm^{-3}

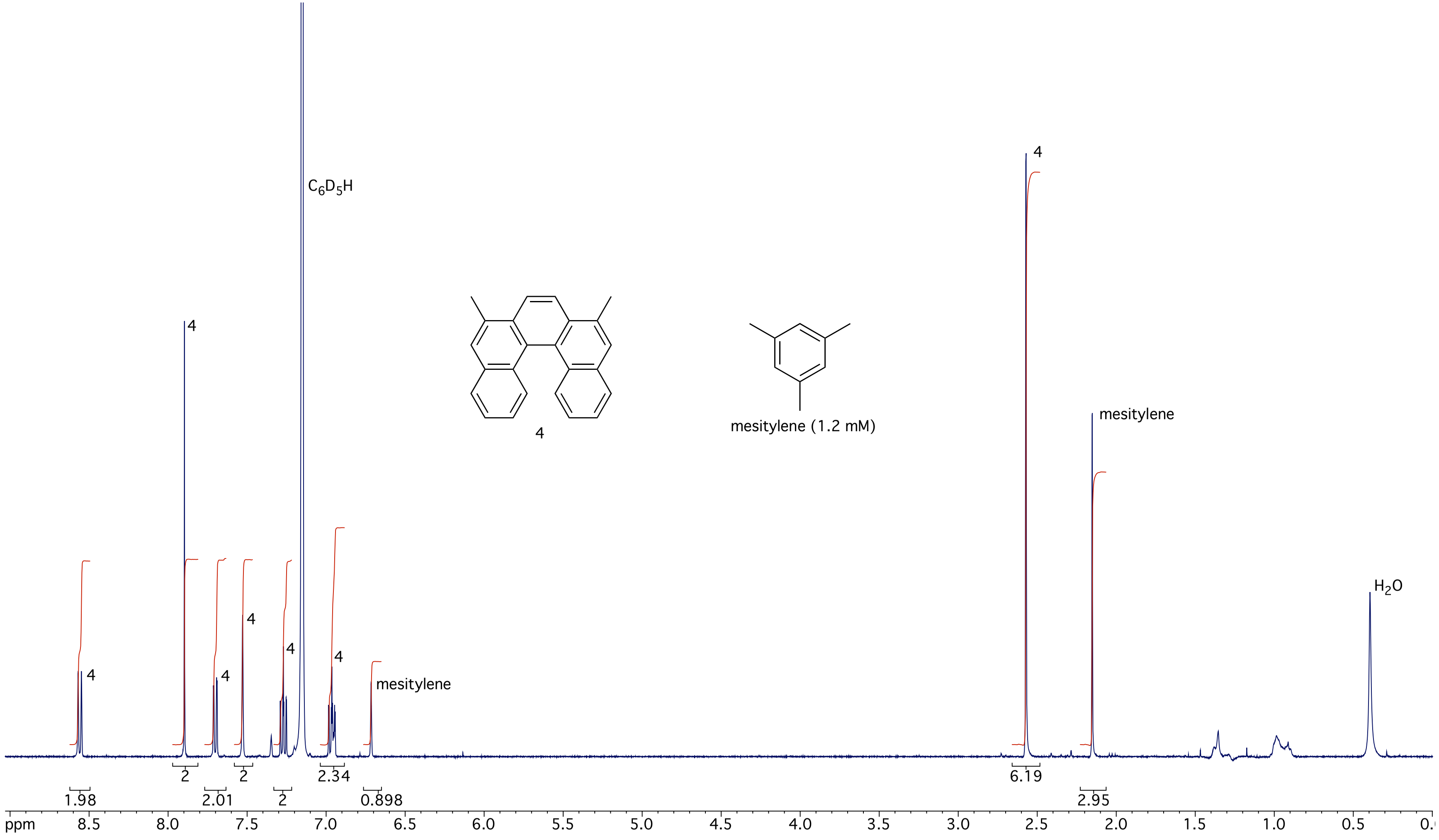

S8.3.5 ^{1}H NMR spectrum for quantification of dihydrohelicene 3 *vs.* mesitylene

[mesitylene] $= 1.20 \pm 0.05$ mmol dm^{-3} $\Longrightarrow$ [*cis*-**1**] $= 0.44 \pm 0.04$ mmol dm^{-3}, [**3**] $= 1.96 \pm 0.15$ mmol dm^{-3}, [**4**] $= 1.77 \pm 0.13$ mmol dm^{-3}

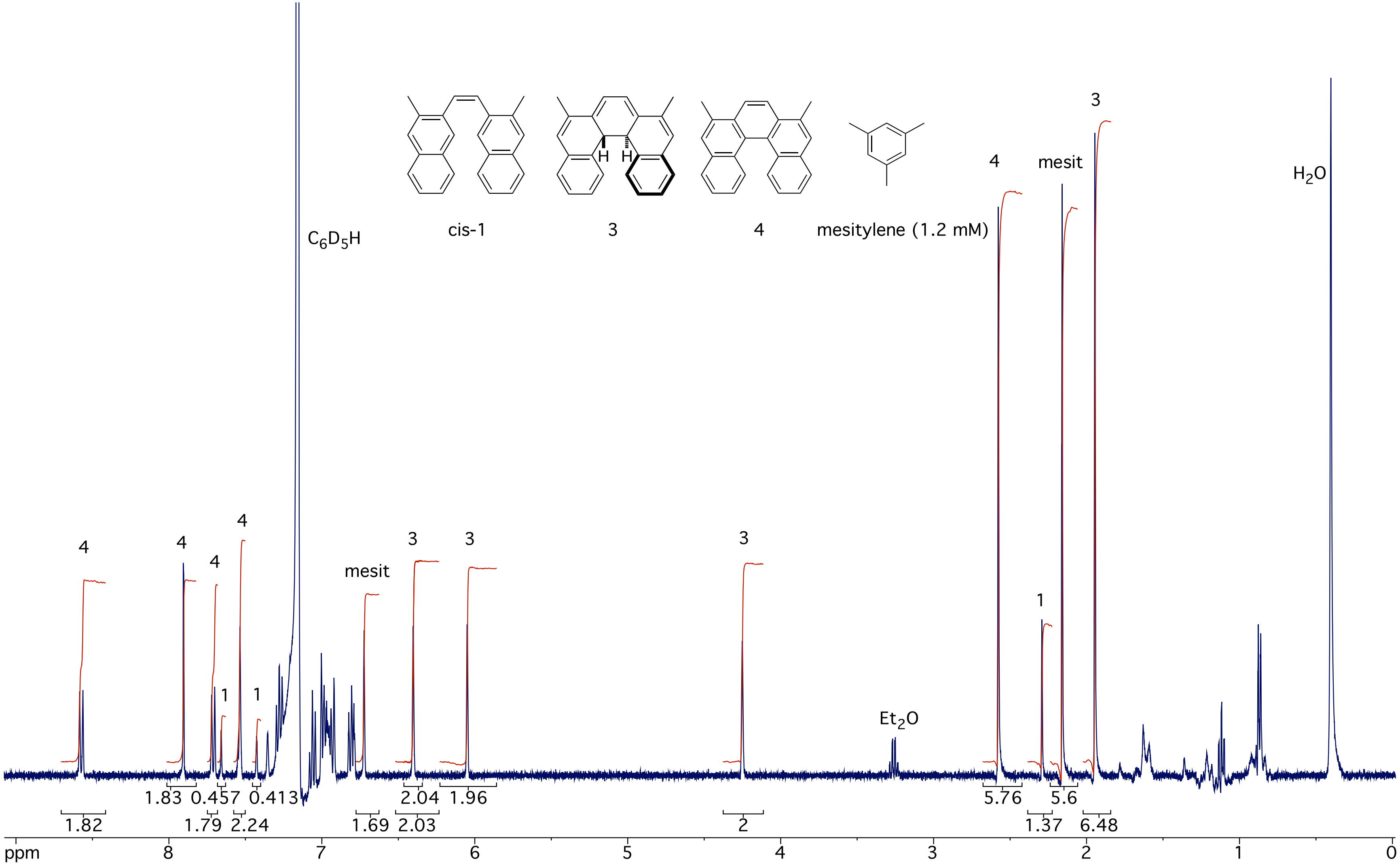

S8.3.6 UV spectrum of diluted NMR sample from S8.3.4 into methylcyclohexane to extract extinction coefficients for dihydrohelicene 3

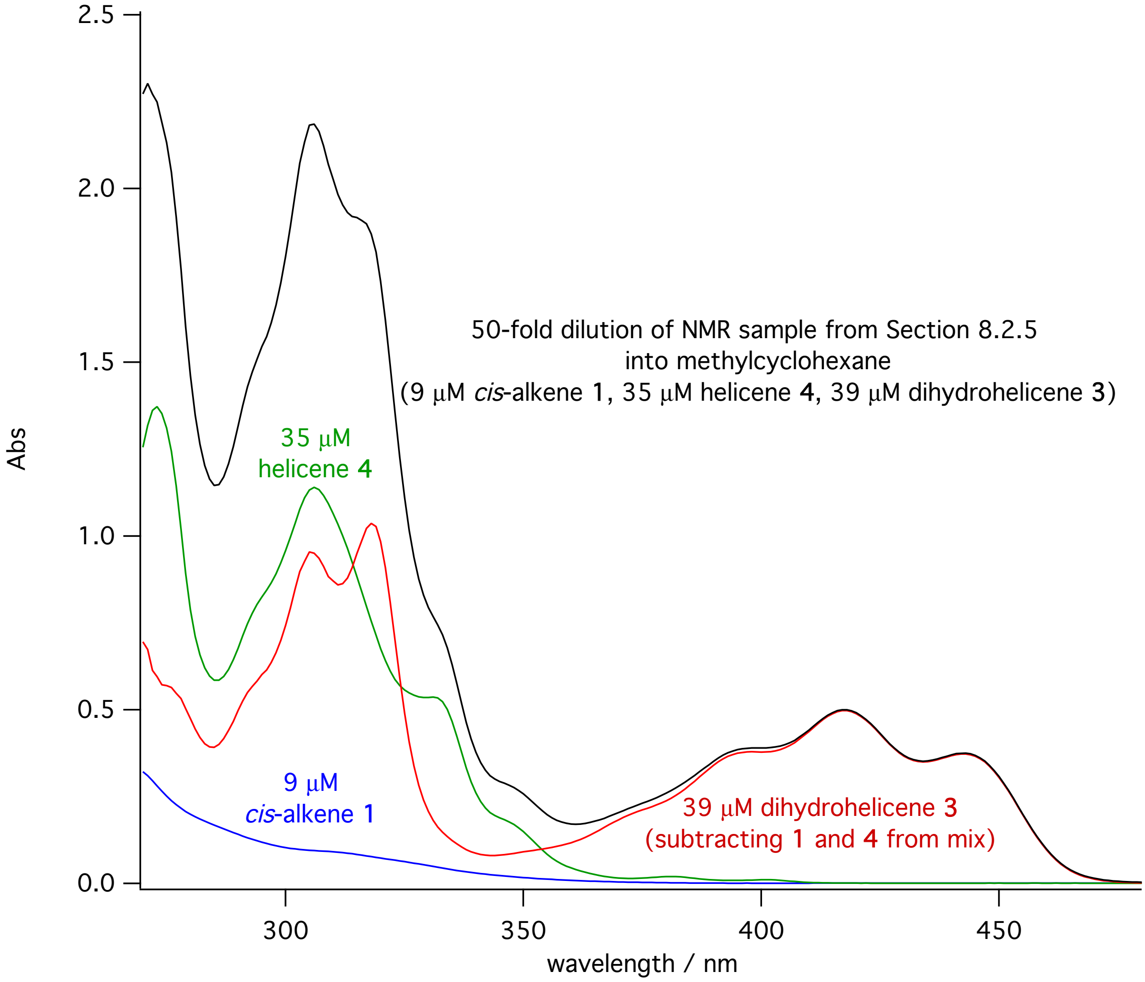

S8.4 Atomic Coordinates for Computed Structures

Atomic coordinates for all structures optimized at the CPCM(methylcyclohexane)/B3LYP/6-31G(d,p) are included in the following tables.

Table S4: Calculated atomic coordinates for (*P*)-**1a**

C	0.669027	2.669289	0.082595
C	-0.669027	2.669289	-0.082595
H	-1.135215	3.648922	-0.180932
H	1.135215	3.648922	0.180932
C	-1.609688	1.529599	-0.115564
C	-1.497973	0.485030	0.787015
C	-2.417713	-0.591877	0.812476
C	-3.499913	-0.591714	-0.124134
C	-3.605788	0.492263	-1.034763
C	-2.706443	1.537871	-1.046648
C	-2.864762	2.667777	-2.036033
C	-2.306536	-1.666735	1.736798
C	-3.220955	-2.696005	1.731642
C	-4.290844	-2.696913	0.801823
C	-4.426429	-1.668307	-0.104415
H	-0.685402	0.489647	1.507095
H	-1.483808	-1.663128	2.446982
H	-3.125792	-3.512978	2.440778
H	-5.005710	-3.514525	0.807697
H	-5.246900	-1.666618	-0.817434
H	-4.429465	0.490095	-1.745351
H	-1.953969	2.819638	-2.625637
H	-3.687833	2.467405	-2.726368
H	-3.078342	3.619875	-1.534212
C	1.609688	1.529599	0.115564
C	1.497973	0.485030	-0.787015
C	2.417713	-0.591877	-0.812476
C	3.499913	-0.591714	0.124134
C	3.605788	0.492263	1.034763
C	2.706443	1.537871	1.046648
C	2.864762	2.667777	2.036033
C	4.426429	-1.668307	0.104415
C	4.290844	-2.696913	-0.801823
C	3.220955	-2.696005	-1.731642
C	2.306536	-1.666735	-1.736798
H	1.483808	-1.663128	-2.446982
H	0.685402	0.489647	-1.507095
H	3.125792	-3.512978	-2.440778
H	5.005710	-3.514525	-0.807697
H	5.246900	-1.666618	0.817434
H	4.429465	0.490095	1.745351
H	3.687833	2.467405	2.726368
H	1.953969	2.819638	2.625637
H	3.078342	3.619875	1.534212

Table S5: Calculated atomic coordinates for the TS connecting (P)-**1a** and (P,R,R)-**2**

C	1.291568	-2.793085	-3.054880
C	0.801797	-3.448349	-1.935525
C	0.631832	-2.765341	-0.711179
C	0.944828	-1.375939	-0.642284
C	1.460859	-0.739208	-1.785949
C	1.634560	-1.433441	-2.977117
C	0.745178	-0.652651	0.610332
C	0.367214	-1.422269	1.806676
C	0.121945	-2.818203	1.710009
C	0.226452	-3.449593	0.481196
C	0.121945	-0.680795	3.011767
C	-0.121945	0.680795	3.011767
C	-0.367214	1.422269	1.806676
C	-0.121945	2.818203	1.710009
C	-0.226452	3.449593	0.481196
C	-0.631832	2.765341	-0.711179
C	-0.944828	1.375939	-0.642284
C	-0.745178	0.652651	0.610332
C	-1.460859	0.739208	-1.785949
C	-1.634560	1.433441	-2.977117
C	-1.291568	2.793085	-3.054880
C	-0.801797	3.448349	-1.935525
H	-0.135212	1.206558	3.962216
H	0.135212	-1.206558	3.962216
C	0.296148	3.611737	2.927146
H	-0.014549	4.513677	0.410721
H	-1.493006	-0.118515	0.788636
C	-0.296148	-3.611737	2.927146
H	0.014549	-4.513677	0.410721
H	1.493006	0.118515	0.788636
H	-1.719172	-0.314238	-1.730634
H	-2.031840	0.922091	-3.848782
H	-0.558642	4.506890	-1.983192
H	-1.421506	3.333935	-3.987804
H	1.719172	0.314238	-1.730634
H	2.031840	-0.922091	-3.848782
H	0.558642	-4.506890	-1.983192
H	1.421506	-3.333935	-3.987804
H	-0.437968	-4.665418	2.674380
H	0.458116	-3.560586	3.721320
H	-1.234146	-3.237086	3.351995
H	0.437968	4.665418	2.674380
H	-0.458116	3.560586	3.721320
H	1.234146	3.237086	3.351995

Table S6: Calculated atomic coordinates for (P, R, R)-**2**

C	1.005967	-1.840643	-1.148322
C	1.505232	-0.699082	-0.514341
C	2.850059	-0.701146	-0.079681
C	3.618889	-1.871407	-0.211228
C	3.087783	-3.017085	-0.796516
C	1.780347	-2.995454	-1.284683
C	3.460735	0.520843	0.420528
C	2.826833	1.720782	0.458958
C	1.426331	1.808800	0.079494
C	0.665845	0.565389	-0.415692
C	0.704706	2.965822	0.152275
C	-0.704706	2.965822	-0.152274
C	-1.426331	1.808800	-0.079494
C	-0.665845	0.565389	0.415692
C	-1.505233	-0.699081	0.514340
C	-2.850059	-0.701145	0.079681
C	-3.460735	0.520843	-0.420529
C	-2.826833	1.720782	-0.458958
C	-3.618890	-1.871407	0.211228
C	-3.087783	-3.017084	0.796516
C	-1.780348	-2.995453	1.284683
C	-1.005967	-1.840643	1.148322
C	-3.548429	2.955987	-0.937330
C	3.548430	2.955986	0.937329
H	-1.182579	3.902096	-0.422457
H	1.182580	3.902095	0.422459
H	-0.352217	0.789637	1.450499
H	0.005390	-1.831138	1.540892
H	-1.362230	-3.870462	1.773243
H	-3.696047	-3.911611	0.893329
H	-4.650737	-1.862097	-0.130768
H	-4.499152	0.457568	-0.737904
H	-3.058606	3.393803	-1.815004
H	-4.581389	2.722967	-1.206484
H	-3.567210	3.732393	-0.163305
H	-0.005390	-1.831138	-1.540892
H	0.352218	0.789637	-1.450500
H	1.362230	-3.870463	-1.773243
H	3.696046	-3.911612	-0.893328
H	4.650736	-1.862098	0.130768
H	4.499153	0.457567	0.737902
H	4.581391	2.722968	1.206478
H	3.058610	3.393799	1.815008
H	3.567205	3.732396	0.163307

Table S7: Calculated atomic coordinates for TS connecting (P, R, R)-**2** and (M, R, R)-**3**

C	3.636237	-1.615935	-1.058065
C	2.832356	-0.571470	-0.564882
C	1.726866	-0.855252	0.279885
C	1.671773	-2.152204	0.801912
C	2.497777	-3.183637	0.346713
C	3.448970	-2.928009	-0.639366
C	3.294506	0.793419	-0.717346
C	2.737867	1.830613	-0.043427
C	1.437388	1.652966	0.579928
C	0.759902	0.271229	0.698146
C	-0.650248	0.343014	-0.039143
C	-1.389549	1.661121	0.257170
C	-0.742710	2.711807	0.836800
C	0.688692	2.748904	0.893356
C	-2.699621	1.833799	-0.358263
C	-3.334414	0.749557	-0.874659
C	-2.924265	-0.601153	-0.533789
C	-1.670751	-0.809692	0.106240
C	-3.846531	-1.651259	-0.652700
C	-3.601685	-2.890141	-0.064197
C	-2.440213	-3.057747	0.682025
C	-1.495790	-2.024557	0.759260
H	-1.276014	3.641070	1.008558
H	4.198375	0.951370	-1.300661
H	1.006905	-2.375382	1.624224
H	4.453675	-1.368308	-1.730364
H	4.078098	-3.724668	-1.024945
C	-3.322873	3.205029	-0.454491
H	-4.277375	0.873238	-1.402160
H	2.397209	-4.176878	0.773522
H	-0.621682	-2.193496	1.357377
C	3.381683	3.195296	-0.084019
H	-4.783021	-1.467897	-1.173376
H	-4.324358	-3.695419	-0.156582
H	-2.249607	-3.990704	1.204153
H	1.148166	3.730874	0.938246
H	0.528029	0.128403	1.764638
H	-0.438312	0.375601	-1.125829
H	4.364883	3.139843	-0.557162
H	2.777169	3.918246	-0.644216
H	3.516390	3.603165	0.924346
H	-4.266098	3.161169	-1.004638
H	-3.541777	3.614372	0.539201
H	-2.662842	3.921221	-0.956868

Table S8: Calculated atomic coordinates for (M,R,R)-**3**

C	1.206653	-1.844995	1.081071
C	1.647713	-0.758829	0.322200
C	3.031729	-0.652512	0.037617
C	3.914060	-1.652025	0.479393
C	3.453444	-2.734603	1.223965
C	2.097302	-2.823730	1.535551
C	3.537813	0.531771	-0.636851
C	2.804216	1.666654	-0.779444
C	1.403860	1.660201	-0.372515
C	0.720648	0.295351	-0.295984
C	0.692742	2.802872	-0.191244
C	-0.692737	2.802873	0.191243
C	-1.403858	1.660203	0.372513
C	-0.720647	0.295352	0.295982
C	-1.647715	-0.758827	-0.322201
C	-3.031730	-0.652509	-0.037617
C	-3.537812	0.531775	0.636851
C	-2.804213	1.666658	0.779443
C	-3.914062	-1.652021	-0.479391
C	-3.453449	-2.734600	-1.223963
C	-2.097308	-2.823730	-1.535550
C	-1.206657	-1.844995	-1.081072
C	-3.439286	2.935297	1.293385
C	3.439292	2.935293	-1.293383
H	-1.172504	3.764683	0.341335
H	1.172510	3.764681	-0.341335
H	-0.628837	0.006827	1.360121
H	-0.154884	-1.955889	-1.317465
H	-1.722997	-3.656368	-2.123590
H	-4.147278	-3.497872	-1.563748
H	-4.972172	-1.558395	-0.248396
H	-4.586108	0.531087	0.927178
H	-3.483481	3.705629	0.513987
H	-4.462212	2.747394	1.628628
H	-2.873815	3.360138	2.130307
H	0.154880	-1.955887	1.317464
H	0.628838	0.006826	-1.360124
H	1.722990	-3.656368	2.123592
H	4.147271	-3.497875	1.563752
H	4.972170	-1.558401	0.248399
H	4.586109	0.531081	-0.927177
H	4.462213	2.747386	-1.628638
H	3.483501	3.705618	-0.513977
H	2.873816	3.360147	-2.130294

Table S9: Calculated atomic coordinates for (*M*)-**4**

C	-1.073984	-1.845404	-1.014881
C	-1.551113	-0.712066	-0.306202
C	-2.947622	-0.664928	-0.014267
C	-3.765633	-1.787592	-0.294821
C	-3.252559	-2.902005	-0.924606
C	-1.898755	-2.911812	-1.316438
C	-3.527020	0.543931	0.479027
C	-2.814082	1.706240	0.548807
C	-1.397829	1.682497	0.252059
C	-0.725406	0.450736	0.007015
C	-0.661982	2.897717	0.171769
C	0.661967	2.897719	-0.171791
C	1.397823	1.682503	-0.252053
C	0.725405	0.450740	-0.007003
C	1.551117	-0.712061	0.306209
C	2.947623	-0.664922	0.014263
C	3.527017	0.543942	-0.479025
C	2.814078	1.706250	-0.548796
C	3.765637	-1.787586	0.294808
C	3.252568	-2.902000	0.924595
C	1.898768	-2.911809	1.316440
C	1.073994	-1.845400	1.014892
C	3.495580	2.996387	-0.936848
C	-3.495589	2.996375	0.936858
H	1.174168	3.840752	-0.323153
H	-1.174194	3.840750	0.323099
H	0.043614	-1.869672	1.344336
H	1.500406	-3.757608	1.868917
H	3.892341	-3.750572	1.147880
H	4.819677	-1.737978	0.034377
H	4.586479	0.542866	-0.723805
H	3.491543	3.721176	-0.113928
H	4.537541	2.810816	-1.208152
H	3.005789	3.477901	-1.790737
H	-0.043600	-1.869677	-1.344316
H	-1.500388	-3.757611	-1.868913
H	-3.892330	-3.750576	-1.147899
H	-4.819676	-1.737985	-0.034398
H	-4.586480	0.542851	0.723812
H	-4.537518	2.810782	1.208272
H	-3.491657	3.721117	0.113894
H	-3.005734	3.477954	1.790670

Table S10: Calculated atomic coordinates for TS for racemization of **4**

C	-2.070620	0.788362	-1.504377
C	-0.781325	0.225558	-1.651468
C	-0.580870	-0.479199	-2.885538
C	-1.679155	-0.831622	-3.704457
C	-2.956856	-0.391769	-3.423041
C	-3.131112	0.487727	-2.345494
C	0.373969	0.384672	-0.737570
C	1.650766	0.338899	-1.396685
C	1.817929	-0.162803	-2.747289
C	0.738653	-0.687784	-3.385834
C	2.830544	0.657761	-0.681178
C	2.830544	0.657761	0.681178
C	1.650766	0.338899	1.396685
C	0.373969	0.384672	0.737570
C	-0.781325	0.225558	1.651468
C	-0.580870	-0.479199	2.885538
C	0.738653	-0.687784	3.385834
C	1.817929	-0.162803	2.747289
C	-2.070620	0.788362	1.504377
C	-3.131112	0.487727	2.345494
C	-2.956856	-0.391769	3.423041
C	-1.679155	-0.831622	3.704457
H	3.761387	0.797214	-1.216598
H	0.853288	-1.183487	4.346392
H	-2.228959	1.562550	0.780386
H	-1.481960	-1.413654	4.600817
H	-3.790807	-0.658062	4.065226
C	3.181495	-0.222507	-3.394087
H	0.853288	-1.183487	-4.346392
H	-4.092553	0.963211	2.175875
H	-2.228959	1.562550	-0.780386
C	3.181495	-0.222507	3.394087
H	-1.481960	-1.413654	-4.600817
H	-3.790807	-0.658062	-4.065226
H	-4.092553	0.963211	-2.175875
H	3.761387	0.797214	1.216598
H	3.104752	-0.653160	-4.395285
H	3.626433	0.774199	-3.496715
H	3.887913	-0.832575	-2.819674
H	3.104752	-0.653160	4.395285
H	3.887913	-0.832575	2.819674
H	3.626433	0.774199	3.496715

Table S11: Calculated atomic coordinates for conformer **1b**

C	-4.451991	0.337172	-1.432142
C	-3.413732	-0.149352	-0.592022
C	-3.355125	0.292966	0.766670
C	-4.337804	1.205775	1.235236
C	-5.330791	1.662021	0.396982
C	-5.388741	1.223401	-0.949535
C	-2.322726	-0.212955	1.598385
C	-1.360761	-1.093352	1.145191
C	-1.403047	-1.513470	-0.230078
C	-2.427377	-1.060551	-1.045169
C	-0.436718	-2.486741	-0.803894
C	0.903915	-2.407065	-0.908499
C	1.802917	-1.291138	-0.560577
C	1.407838	0.027571	-0.724404
C	2.267068	1.115046	-0.430991
C	3.588122	0.837380	0.042750
C	3.980771	-0.518757	0.194241
C	3.137911	-1.570029	-0.096977
C	4.455086	1.922507	0.338081
C	4.035390	3.223861	0.170390
C	2.727906	3.499594	-0.301534
C	1.864387	2.468525	-0.596355
C	3.610684	-2.992911	0.085573
C	-0.319018	-1.621808	2.101391
H	1.397159	-3.278636	-1.335170
H	-0.896926	-3.381264	-1.224258
H	0.413802	0.241943	-1.102830
H	0.860285	2.675340	-0.957206
H	2.409802	4.530046	-0.429601
H	4.707401	4.045890	0.399057
H	5.458130	1.709248	0.698636
H	4.986026	-0.726465	0.554272
H	3.651977	-3.533636	-0.868093
H	4.613326	-3.015454	0.519611
H	2.943728	-3.561014	0.743285
H	-4.493189	-0.001305	-2.464317
H	-2.473639	-1.402113	-2.076928
H	-6.177404	1.590343	-1.599852
H	-6.075915	2.360952	0.765640
H	-4.292494	1.539620	2.268714
H	-2.301935	0.102378	2.639250
H	-0.584231	-1.374570	3.132667
H	0.669700	-1.198245	1.897609
H	-0.220590	-2.709212	2.023228

Table S12: Calculated atomic coordinates for TS connecting (*P*)-**1a** with **1b**

C	-3.751782	0.719636	-1.847042
C	-3.096436	0.000983	-0.810952
C	-3.499830	0.214875	0.544876
C	-4.543971	1.140639	0.813010
C	-5.160318	1.823001	-0.212473
C	-4.760710	1.610576	-1.555348
C	-2.839892	-0.509701	1.572648
C	-1.825960	-1.407433	1.309166
C	-1.417556	-1.614361	-0.052293
C	-2.052580	-0.926293	-1.068416
C	-0.370412	-2.623089	-0.380604
C	0.962714	-2.476432	-0.513058
C	1.836951	-1.299440	-0.357260
C	1.349500	-0.031861	-0.065851
C	2.195485	1.092462	0.091378
C	3.606735	0.918692	-0.056510
C	4.094004	-0.379756	-0.355849
C	3.263686	-1.470073	-0.507550
C	4.460336	2.042625	0.100257
C	3.939656	3.284801	0.390066
C	2.540389	3.458963	0.536140
C	1.688274	2.387520	0.389827
C	3.874191	-2.817245	-0.823622
C	-1.144943	-2.156735	2.428167
H	1.489325	-3.390680	-0.768736
H	-0.752049	-3.633701	-0.534804
H	0.283040	0.121070	0.043297
H	0.614810	2.516583	0.500446
H	2.143321	4.443704	0.764128
H	4.601256	4.137999	0.507951
H	5.532927	1.907020	-0.012359
H	5.167801	-0.511643	-0.467782
H	3.513979	-3.219323	-1.777643
H	4.961733	-2.735619	-0.890831
H	3.645400	-3.563067	-0.053748
H	-3.442168	0.554605	-2.875783
H	-1.743957	-1.091643	-2.097560
H	-5.254722	2.154800	-2.354833
H	-5.957240	2.528126	0.004840
H	-4.849306	1.301620	1.843716
H	-3.152220	-0.345674	2.601645
H	-1.578740	-1.893963	3.396365
H	-0.073097	-1.932957	2.461173
H	-1.235275	-3.241393	2.298277

Table S13: Calculated atomic coordinates for the TS for racemization of **1b**

C	2.722471	-2.262255	0.809113
C	2.640700	-0.890035	0.450039
C	3.536208	-0.377723	-0.539617
C	4.476042	-1.258121	-1.138721
C	4.529763	-2.585163	-0.772330
C	3.645287	-3.092072	0.211499
C	3.453564	0.999055	-0.878392
C	2.542196	1.850252	-0.291316
C	1.624363	1.329979	0.688931
C	1.703266	-0.003688	1.042712
C	0.672886	2.266410	1.335460
C	-0.672886	2.266410	1.335460
C	-1.624362	1.329980	0.688930
C	-2.542194	1.850253	-0.291318
C	-3.453563	0.999056	-0.878394
C	-3.536208	-0.377722	-0.539617
C	-2.640701	-0.890034	0.450039
C	-1.703266	-0.003686	1.042713
C	-4.476042	-1.258120	-1.138721
C	-4.529765	-2.585161	-0.772330
C	-3.645289	-3.092070	0.211501
C	-2.722473	-2.262253	0.809115
C	-2.496224	3.307414	-0.682908
C	2.496228	3.307413	-0.682905
H	1.143484	3.121710	1.822429
H	-1.143483	3.121711	1.822429
H	1.054812	-0.390590	1.820601
H	2.041683	-2.648415	1.563332
H	3.698108	-4.139812	0.492280
H	5.252743	-3.249143	-1.237046
H	5.154423	-0.866565	-1.892335
H	4.138293	1.387579	-1.628990
H	2.739953	3.961798	0.163208
H	3.209913	3.519302	-1.482957
H	1.498257	3.599770	-1.027346
H	-2.041686	-2.648413	1.563334
H	-1.054814	-0.390589	1.820603
H	-3.698111	-4.139810	0.492283
H	-5.252745	-3.249141	-1.237045
H	-5.154422	-0.866564	-1.892336
H	-4.138291	1.387580	-1.628993
H	-3.209910	3.519303	-1.482960
H	-1.498254	3.599769	-1.027350
H	-2.739948	3.961799	0.163204

Notes

[1]W. Kuhn, *Trans. Faraday Soc.*, 1930, **26**, 293–308.

[2]For some discussion, see: S. Logan, *J. Chem. Ed.*, 1997, **74**, 1303 and M. Hippler, *J. Chem. Ed.*, 2005, **82**, 37.

[3]Defined as the number of photons incident on a sample per unit time.

[4]For an elegant example of the application of flow chemistry to synthetic photochemistry, see: K. G. Maskill, J. P. Knowles, L. D. Eliot, R. W. Alder and K. I. Booker-Milburn, *Angew. Chem. Int. Ed.*, 2013, **52**, 1499–1502.

[5]For examples, see: B. Feringa and R. van Delden, *Angew. Chem. Int. Ed.*, 1999, **38**, 3418–3438; Y, Inoue, *Chem. Rev.*, 1992, **92**, 741–770.

[6]This result is general to any PSS.

[7]At many wavelengths, the total rate is the sum of the rates at all wavelengths. For broadband light, the sum is replaced by an integral over the entire spectrum where the photon flux in the rate equations is replaced by the emission standard of the light source and the extinction coefficients and quantum yields are replaced by the relevant functions of wavelength.

[8]It can be shown, by differentiating this expression, that, if A does not absorb the second wavelength, the change in PSS *ee* with power should be smooth and not pass through a maximum.

[9](a) K. A. Muszkat, *Topp. Curr. Chem.*, 1980, **88**, 89–143; (b) K. A. Muszkat, A. Jakob, N. Castel and E. Fischer, *J. Photochem. Photobiol. A: Chem.*, 1991, **60**, 193–205; (c) K. A. Muszkat, M. Eisenstein, E. Fischer, A. Wagner, Y. Ittah and W. Lüttke, *J. Am. Chem. Soc.*, 1997, **119**, 9351–9360.

[10]For the lamp output standards, see http://www.luzchem.com/handbook/LESUVA011.pdf.

[11]*trans*-**1** rapidly isomerizes to *cis*-**1** under the reaction conditions.

[12]Gaussian 09, Revision C.01, M. J. Frisch, G. W. Trucks, H. B Schlegel, G. E. Scuseria, M. A. Robb, J. R. Cheeseman, G. Scalmani, V. Barone, B. Mennucci, G. A. Petersson, H. Nakatsuji, M. Caricato, X. Li, H. P. Hratchian, A. F. Izmaylov, J. Bloino, G. Zheng, J. L. Sonnenberg, M. Hada, M. Ehara, K. Toyota, R. Fukuda, J. Hasegawa, M. Ishida, T. Nakajima, Y. Honda, O. Kitao, H. Nakai, T. Vreven, J. A. Montgomery Jr., J. E. Peralta, F. Ogliaro, M. Bearpark, J. J. Heyd, E. Brothers, K. N. Kudin, V. N. Staroverov, R. Kobayashi, J. Normand, K. Raghavachari, A. Rendell, J. C. Burant, S. S. Iyengar, J. Tomasi, M. Cossi, N. Rega, M. J. Millam, M. Klene, J. E. Knox, J. B. Cross, V. Bakken, C. Adamo, J. Jaramillo, R. Gomperts, R. E. Stratmann, O. Yazyev, A. J. Austin, R. Cammi, C. Pomelli, J. W. Ochterski, R. L. Martin, K. Morokuma, V. G. Zakrzewski, G. A. Voth, P. Salvador, J. J. Dannenberg, S. Dapprich, A. D. Daniels, O. Farkas, J. B. Foresman, J. V. Ortiz, J. Cioslowski and D. J. Fox, Gaussian, Inc., Wallingford CT, 2009.

[13]For the B3 functional, see: A. D. Becke, *J.Chem.Phys.*, 1993, **98**, 5648–5652. For the LYP functional, see: C. Lee, W. Yang and R. G. Parr, *Phys. Rev. B*, 1988, **37**, 785–789.

[14]Y. Zhao and D. G. Truhlar, *Theor. Chem. Acc.*, 2006, **120**, 215–241.

[15]J.-D. Chai and M. Head-Gordon, *Phys. Chem. Chem. Phys.*, 2008, **10**, 6615–6620.

[16]S. Grimme, *J. Comp. Chem.*, 2006, **27**, 1787–1799.

[17]M. Cossi, N. Rega, G. Scalmani, and V. Barone, *J. Comp. Chem.*, 2003, **24**, 669–681.

[18]T. Yanai, D. P. Tew, N. C. Handy, *Chem. Phys. Lett*, 2004, **393**, 51–57.

[19]GaussView, Version 5.09, R. Dennington, T. Keith and J. Millam, Semichem Inc., Shawnee Mission, KS, 2009.

[20]Y. Nakai, T. Mori and Y. Inoue, *J. Phys. Chem. A*, 2012, **116**, 7372-7385.

[21]W. J. Bernstein, M. Calvin and O. Buchardt, *J. Am. Chem. Soc.*, 1972, **94**, 494–498.

[22]A. Moradpour, J. F. Nicoud, G. Balavoine and H. Kagan, *J. Am. Chem. Soc.*, 1971, **93**, 2353–2354.

[23]For the effect of helicene length on racemization rate, see: R. H. Martin and M. J. Marchant, *Tetrahedron*, 1974, **30**, 347–349.

[24]D. K. Judge, P. Haycock, R. D. Richardson, and M. J. Fuchter, *Synlett*, 2013, **24**, 2365–2369.

[25] C. Goedicke, H. Stedemeyer, *Tetrahedron*, 1970, **11**, 937–940.
